# Influence of Tacticity on the Self-Assembly of Poly(ethylene glycol)-*b*-poly(lactic acid) Block Copolymers

**DOI:** 10.1021/acsmacrolett.4c00758

**Published:** 2025-01-06

**Authors:** Sjoerd J. Rijpkema, B. Jelle Toebes, Jules van Vlaenderen, Liban van Haren, Daniela A. Wilson

**Affiliations:** † Institute for Molecules and Materials, Radboud University, Heyendaalseweg 135 6525 AJ, Nijmegen, The Netherlands

## Abstract

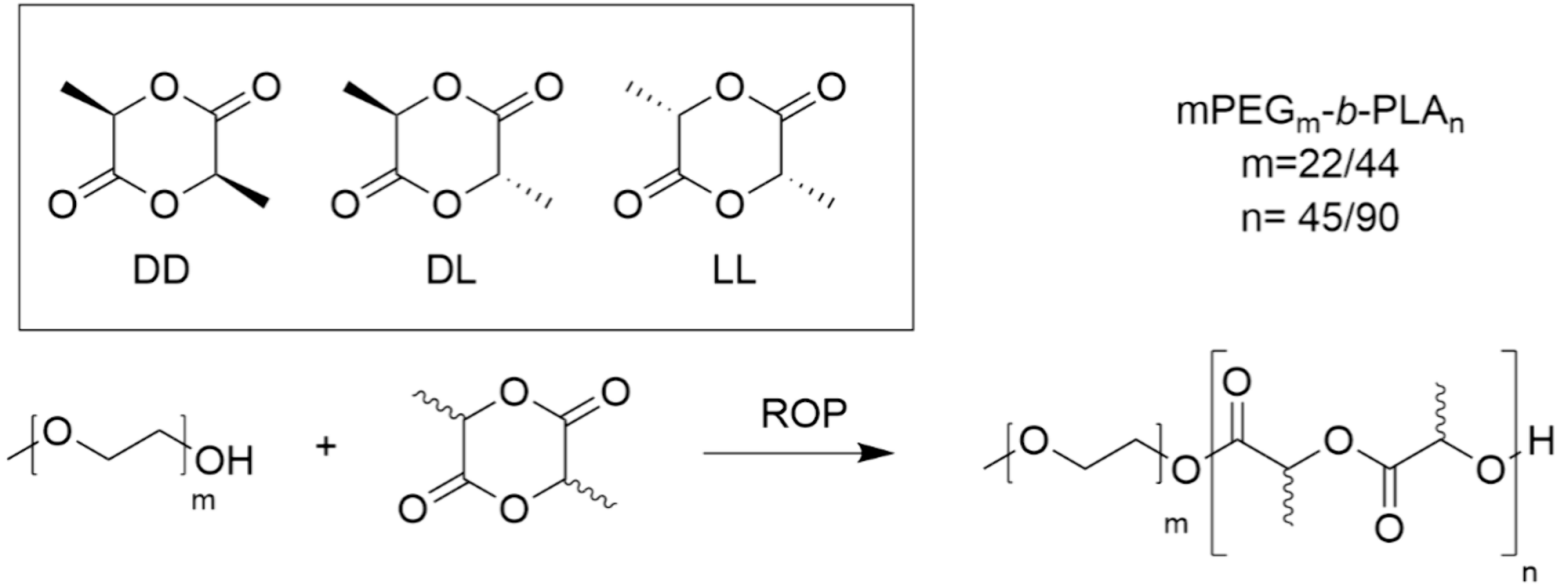

Lactide, possessing two stereocenters and thus three distinct configurations (DD, DL, and LL), serves as a captivating building block for polymers and self-assembly. Notably, polylactide (PLA) exhibits stereocomplexation, displaying heightened interactions between different configurations compared with interactions within the same configuration. This characteristic renders PLA an intriguing subject for investigating self-assembly behavior. In this study, 22 PEG-*b*-PLA polymers were synthesized and self-assembled, with analysis conducted through NMR and cryo-TEM techniques. A range of morphologies, including vesicles, diamond-shaped lamellae, and branched networks, were achieved by manipulating the tacticities. Enhanced comprehension of self-assembly interactions holds promise for advancing molecular recognition, self-replication, and asymmetric catalysis.

Spontaneous molecular self-assembly, orchestrated by noncovalent interactions, can cause the formation of intricate nanostructures, demonstrating diverse shapes and sizes.^1,2^ Nature serves as a prime example of this phenomenon, showcasing the spontaneous organization of the membranes and proteins. Particularly noteworthy is the significance of tacticity in the self-assembly of macromolecules, where the disposition of chiral centers plays a pivotal role in determining symmetry and supramolecular interactions, consequently giving rise to templating effects.^3^ Consequently, polymers with iso- or syndiotactic structures crystallize far more easily compared to their atactic counterparts.^4^

The effects of chirality on supramolecular self-assembly are therefore crucial for specific applications across scientific disciplines,^5^ ranging from molecular recognition^6^ to self-replication^7^ and asymmetric catalysis.^8^ Investigations into the impact of chiral structures on self-assembly behavior, including the influence of trace amounts of chiral monomers on larger structures, are prevalent.^9,10^ This can be seen in many biological materials, which often self-assemble into helical or twisted aggregates, emphasizing the role of orientation and chirality in shaping their self-assembled structures.^11^ Chiral amphiphiles and peptides can assemble into helical structures and form nanotubes.^12^ Similarly, the self-assembly of block copolymers, analogous to crystallization, is dictated by ordered syndiotactic or isotactic regions, yielding diverse supramolecular chiral nanostructures.^13−15^ As tactic polymers are more easily able to template while atactic polymers are not, combining these can lead to a variety of nanostructures.^3,16,17^ Beyond chirality, parameters such as concentration, temperature, and solvents modulate supramolecular self-assembly, altering the polarity and miscibility of the building blocks.^16,18^ Even minute amounts of water in organic solvents have been identified as influential factors.^19^

In addition to the structural diversity afforded by chiral nanostructures, their utility extends to serving as nanoreactors, wherein chirality influences product formation. Commonly, these include the use of chiral ligands or metal centers,^20^ showcasing high enantioselectivities.^21,22^ While several instances of nanostructures self-assembled from achiral building blocks that include chiral components are known, there are few examples of nanoreactors made from chiral building blocks. Of those, there are chiral micelles that can selectively polymerize an achiral monomer^23^ and an α-helical peptide vesicle able to selectively encapsulate a single enantiomer from a racemic mixture solution.^24^ Recently, there has been interest in crystalsomes, crystalline capsules that are formed by controlling polymer crystallization to break translational symmetry.^25−27^ However, there is still a lack of understanding of how the tacticity of the polymer gives rise to various structures.

This study focuses on polylactide (PLA) as a promising candidate for the formation of a broad spectrum of nanostructures. It is synthesized from lactide, a cyclic molecule with two chiral centers. PLA is an excellent option to study the effect of tacticity on self-assembly, as lactide allows for three different possible conformations (DD, DL, and LL) to be present in the polymer. PLA, with its atactic, isotactic, and syndiotactic forms, represents therefore a versatile platform.^28,29^ Previous investigations into polystyrene-*b*-polylactic acid (PS-*b*-PLA) have only focused on a singular tacticity,^27,30,31^ while it is known that a combination of tacticities can induce stereocomplexation.^32^ Our research addresses this gap by exploring a library of PEG-*b*-PLA polymers with diverse compositions and tacticities. Using these different building blocks, various versatile polymer compositions can be made. In our group, nanostructures have already successfully been made from PEG-*b*-PDLLA, but the influence of tacticity had not yet been investigated.^33,34^ This endeavor aims to unravel the self-assembly capabilities of these polymers, investigating the influence of their chirality on their nanostructure. In this study, we aim to discover the effect of tacticity on the self-assembly. To achieve this goal, a library of 22 PEG-*b*-PLA polymers with a systematic variation of lengths, ratios and tacticities was made, as outlined in Scheme 1.

**Scheme 1:**
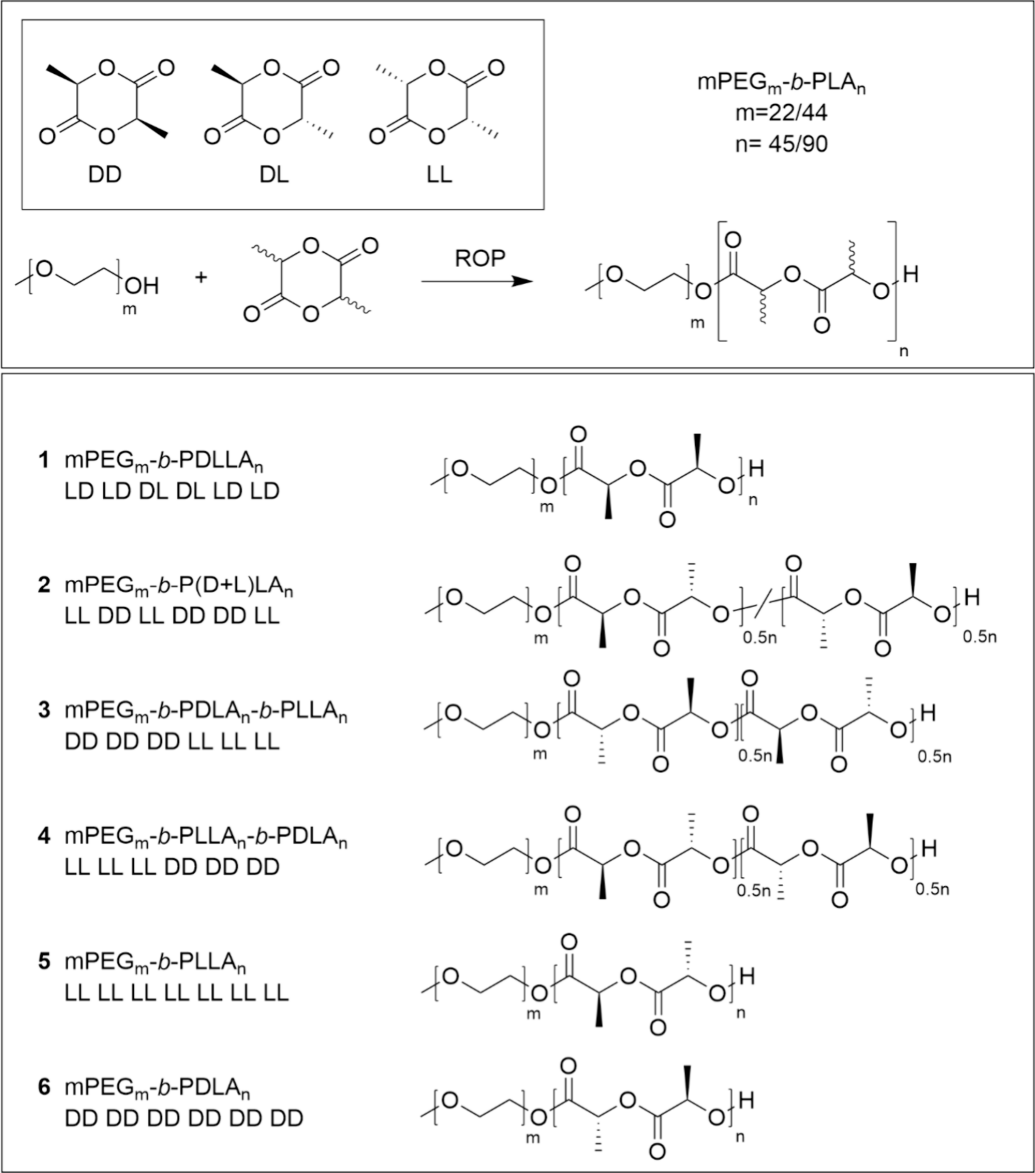
Formation of Stereoselective PEG–PLA Polymers for Self-Assembly Top: Formation of amphiphilic poly(ethylene glycol)-*b*-polylactide (PEG-*b*-PLA) block copolymers with various compositions of lactide monomers (DD, DL, and/or LL). Bottom: Overview of the PEG-*b*-PLA polymers synthesized: PEG_22_-*b*-PDLLA_45_**1a**, PEG_44_-*b*-PDLLA_45_**1b**, PEG_22_-*b*-PDLLA_90_**1c**, PEG_44_-*b*-PDLLA_90_**1d**, PEG_22_-*b*-P(D+L)LA_45_**2a**, PEG_44_-*b*-P(D+L)LA_45_**2b**, PEG_22_-*b*-P(D+L)LA_90_**2c**, PEG_44_-*b*-P(D+L)LA_90_**2d**, PEG_22_-*b*-PDLA_22_-*b*-PLLA_22_**3a**, PEG_44_-*b*-PDLA_22_-*b*-PLLA_22_**3b**, PEG_22_-*b*-PDLA_45_-*b*-PLLA_45_**3c**, PEG_44_-*b*-PDLA_45_-*b*-PLLA_45_**3d**, PEG_22_-*b*-PLLA_45_-*b*-PDLA_45_**4a**, PEG_44_-*b*-PLLA_45_-*b*-PDLA_45_**4b**, PEG_22_-*b*-PLLA_45_-*b*-PDLA_45_**4c**, PEG_44_-*b*-PLLA_45_-*b*-PDLA_45_**4d**, PEG_22_-*b*-PLLA_45_**5a**, PEG_22_-*b*-PLLA_90_**5b**, PEG_44_-*b*-PLLA_90_**5c**, PEG_22_-*b*-PDLA_45_**6a**, PEG_22_-*b*-PDLA_90_**6b**, PEG_44_-*b*-PDLA_90_**6c**.

Several enantiomeric methoxypoly(ethylene glycol)-*b*-polylactide (mPEG-*b*-PLA) block copolymers were synthesized using Ring Opening Polymerization (ROP). As the morphology of the self-assembled structures is dependent on the length of the polymers, several lengths were created. Two different lengths of PEG (22 and 44 units, respectively) were used, and the PLA sections were aimed to be either 45 or 90 lactide units in length, forming four different compositions (labeled a-d). For the PLA segment, different enantiomers were created. Using only the d-lactic acid (DLA), l-lactic acid (LLA), or dl-lactic acid (DLLA) yielded diblock copolymers (**1**, **5**, and **6**). By mixing DLA and LLA, we made random copolymer **2** was made. Finally, triblock copolymers were made by sequentially polymerizing DLA and LLA (**3** and **4**), creating in total 22 different polymers (Scheme 1). For the polymerization of DLLA, the organic catalyst DBt was used, while we found that the polymerizations of LLA and DLA proceeded only when Sn(oct)_2_ was used as a catalyst. These reactions progressed smoothly, providing the desired polymers in high yields. The product compositions were calculated from their respective ^1^H NMR and GPC spectra (Table S1 and Figures S1–S3).

Polymerization was monitored by ^1^H NMR, specifically via the CH backbone. The methanetriyl hydrogen of each lactide monomer shows a quartet signal at 5.04 ppm, but since polymerization results in the deshielding of this group, the signal of the polymer shifts upward to 5.19 ppm. The different polymer compositions can be distinguished by their respective changes in multiplicity and chemical shift (Figure 1). Polymer **1** consists of PEG-*b*-PDLLA, which is atactic, thus making the polymer amorphous. The NMR reflects this, as it shows a multiplet at 5.2 ppm, indicating the alternating, nearly random orientation of the d- and l-lactide monomers. Despite this, a quartet can be seen at the same position as the multiplet, showing that there is some order in the chain. This can be explained by the structure of the monomer. Due to the cyclic d,l monomer, no more than two repeats can appear in the chain. This polymer can be considered atactic but with a syndiotactic character. Polymer **2** consist of PEG linked to a mixture of PDLA and PLLA. This will also create an atactic polymer, as the ratio of d and l conformations is the same as for polymer **1**. However, due to the monomers used, longer repeats are now possible in the chain, making this polymer slightly more isotactic. This can also be seen with NMR, as there are still multiple peaks visible at 5.2 ppm, yet a clearer overlap of two regions is visible. Polymers **3** and **4** contain two crystalline regions, as the PEG is linked to two blocks of PLLA and PDLA. The NMR shows a quartet at 5.19 ppm, with a small signal upfield, most likely originating from the ends between the two PLA blocks. Finally, polymers **5** and **6** are crystalline and contain either PEG-*b*-PLLA or PEG-*b*-PDLA, respectively. The NMR spectrum shows a neat quartet for these polymers at 5.19 ppm, indicating successful isotactic polymerization. Additionally, all polymers show a multiplet with a low intensity at 4.3 ppm. Analysis using 2D NMR techniques indicate that this peak is situated between the bulk PLA and PEG parts of the polymer (Figure S4). We consider this multiplet to be caused by the initial one or two repeating units of the PLA part of the block polymer.

**Figure 1:**
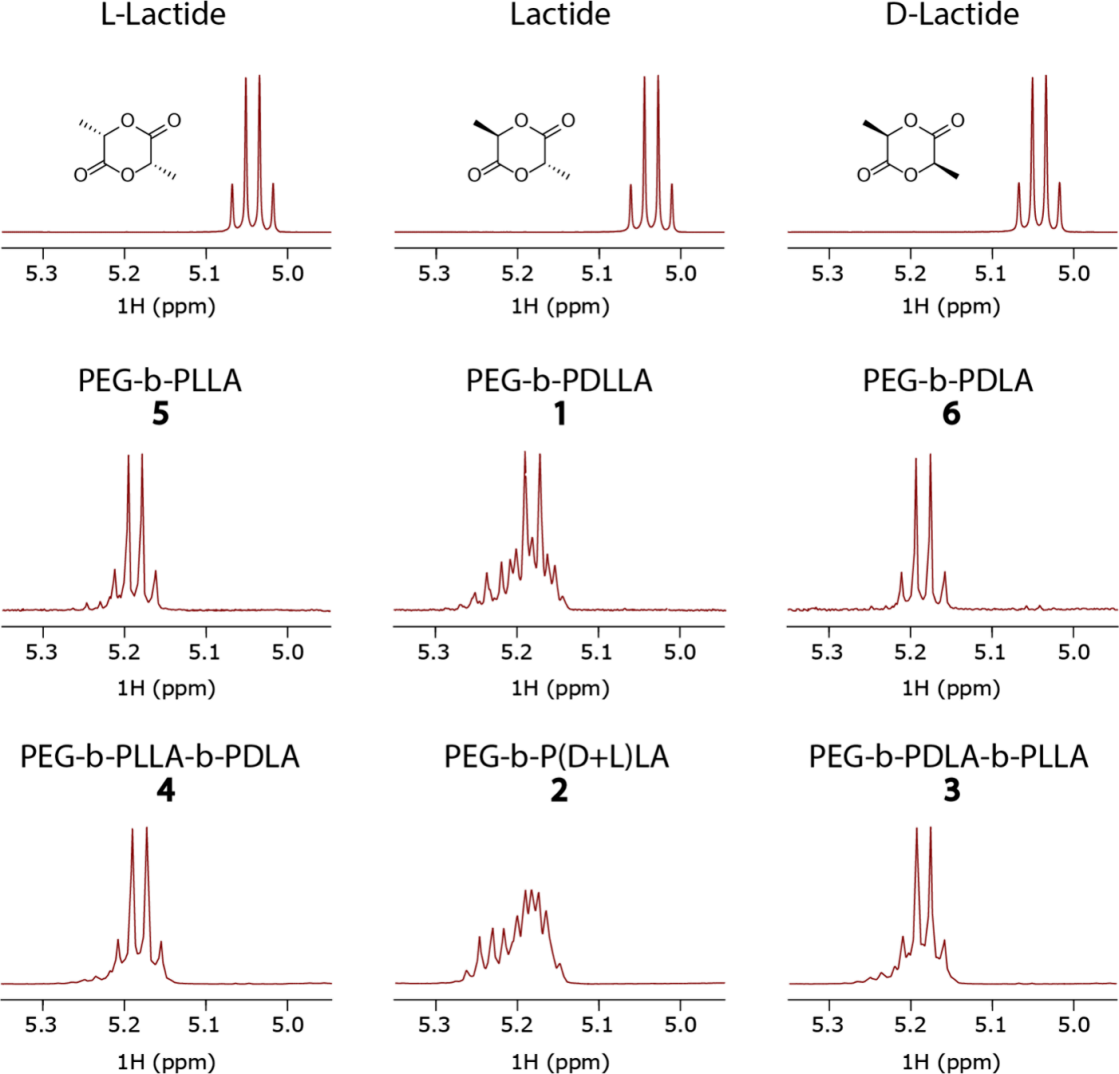
NMR signal of the methanetriyl group of all lactide monomers and polymers. The lactide monomers show a quartet signal at 5.04 ppm, but polymerization shifted the signal upfield to 5.19 ppm with a change in multiplicity and chemical shift, depending on the polymer composition.

All polymers were formed in a relatively high yield. The triblock copolymers **3** and **4** had a more difficult synthesis, as the solubility of the polymer formed became decreasingly lower in the second phase of the polymerization. To combat this, the solvent used in the reaction mixture was doubled. This impacted the polymerization somewhat as the observed lengths on NMR differ more from the desired lengths than in the other polymers formed. The reason for this is that stereocomplexation between PLLA and PDLA can occur in solution during polymerization, crashing the polymer out of solution.^31^ Despite this, we managed to obtain these polymers and were also able to determine their PDI with GPC by measuring at lower concentrations. It must be noted, however, that solubility issues may make the measured PDIs less reliable compared to the other polymers. PDIs for every polymer made were found within the expected range of the respective catalyst used.

To investigate the effect of different tacticities on self-assembly, each polymer (mixture) was first dissolved in THF and 1,4-dioxane (1 mL, 4:1 v/v). We chose this solvent mixture for the self-assembly of the block copolymers because this is a commonly used solvent for a variety of polymers, including PEG-*b*-PS and PEG-*b*-PLA. Although there are examples in literature where different solvents such as toluene are used, these proceed via a different mechanism, namely emulsion-solution crystallization, which is beyond the scope of this research.^27^

After solvation, ultrapure Milli-Q water was slowly added to induce self-assembly. We found that adding Milli-Q at a rate of 1 mL/h resulted in the least number of defects in the structure. Afterward, the samples were dialyzed against Milli-Q to remove the organic solvent and kinetically rigidify their morphology. The samples were then visualized by cryo-Transmission Electron Microscopy (cryo-TEM). In Figure 2 the self-assembly of all the PEG_22_-*b*-PLA_45_ polymers is shown. From **1**, spherical polymersomes of around 450 nm were formed with small PDI (∼0.1), as were previously known.^35^ For **2** similar morphologies were expected and also obtained, though slightly smaller at 250 nm and with a higher PDI (∼0.2). The triblock copolymers **3** and **4** gave large branched networks, while **5** and **6** formed diamond shaped structures, sometimes stacking on top of each other.

**Figure 2:**
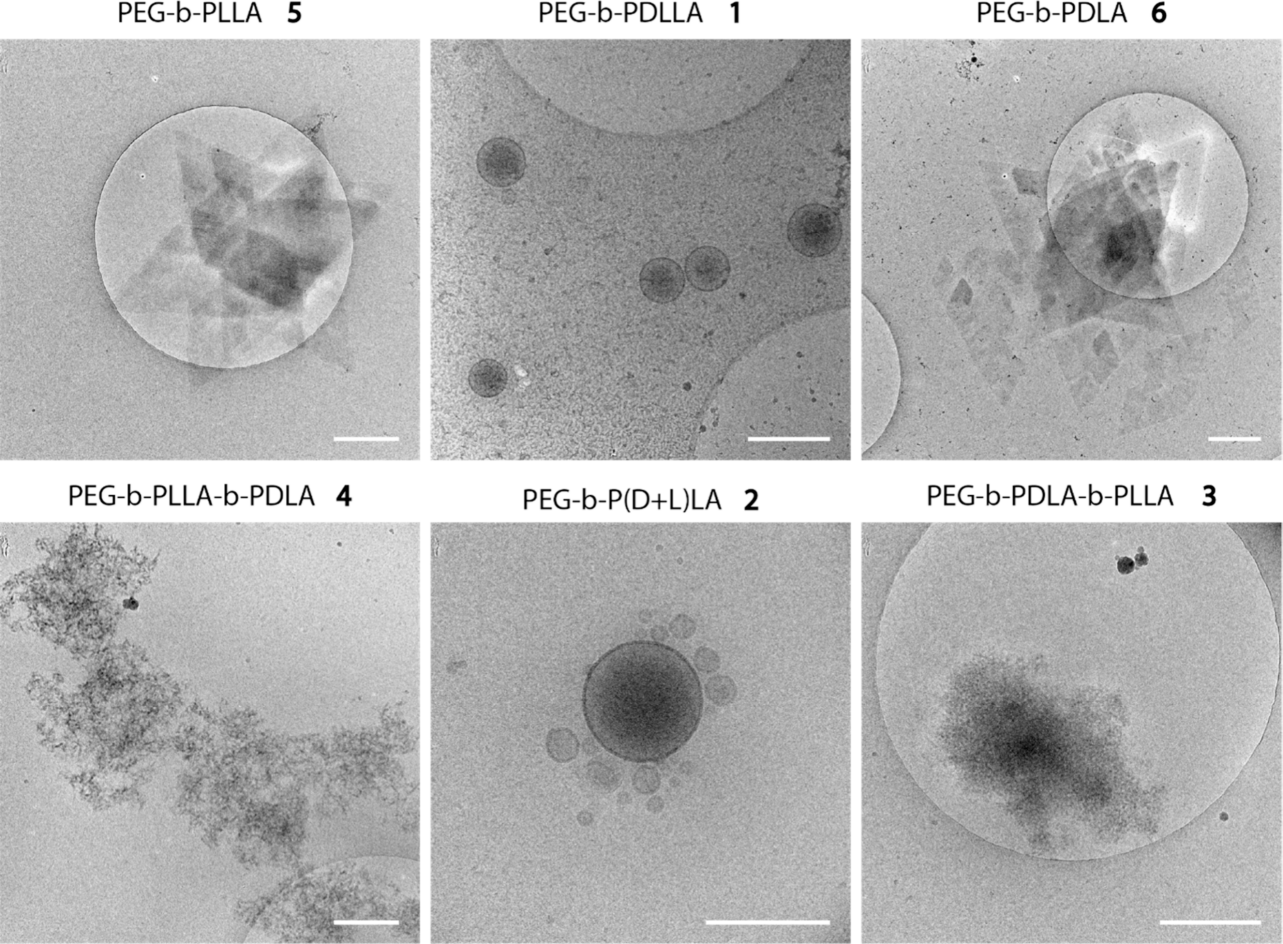
Cryo-TEM pictures of all of the PEG_22_-*b*-PLA_45_ polymer self-assemblies. Top left: diamond shaped lamellar structures from PEG-*b*-PLLA **5**. Top middle: polymersomes from PEG-*b*-PDLLA **1**. Top right: diamond shaped lamellar structures from PEG-*b*-PDLA **6**. Bottom left: stereocomplex crystallites from PEG-*b*-PLLA-*b*-PDLA **4**. Bottom middle: polymersomes from PEG-*b*-P(D+L)LA **2**. Bottom right: stereocomplex crystallites from PEG-*b*-PDLA-*b*-PLLA **3**. Scale bar: 500 nm.

For polymersomes composed of PEG-*b*-PDLLA, the morphology is tunable by controlling various factors. With the four different lengths of **1**, various morphologies could be created, varying from micelles, worms, spherical polymersomes, and Large Compound Vesicles (LCVs) (Figure S5). An overview of the obtained morphologies of this polymer is given in Table S2. Similar results were obtained with each length of **2**, although small differences were observed (Figure S6). A difference in the size distribution was noticed between the vesicles obtained from **1a** and **2a**. Micelles were formed for both **1b** and **2b**, although the size was larger for **2b**. Two types of LCVs were obtained for both **1c** and **2c** respectively. Finally, short worm-like structures were obtained with **1d**, whereas **2d** showed long worms.

As described during the synthesis part, **3** and **4** were difficult to dissolve in organic solvent, resulting in aggregated structures even before the addition of Milli-Q. As these polymers comprise both PLLA and PDLA, stereocomplexation occurs in solution when the polymer concentration exceeds a critical level, forming stereocomplex crystallites. The formed PLA crystallites have higher stability and are formed at a much lower concentration compared with the homocrystallites of either PLLA or PDLA, making them insoluble even in good solvents. For all lengths, large branched networks could be seen on cryo-TEM (Figures S7 and S8). In general, smaller structures were observed with longer polymers. Interestingly, **3** formed a slightly denser structure compared to **4**.

Every length of **5** and **6** formed diamond shaped lamellar structures due to the isotactic structure of PLLA and PDLA (Figure S9). The lack of any other structures obtained can be explained by the speed of the shape transformation of these polymers. In recent literature, the ROP-induced crystallization-driven self-assembly of PEG-*b*-PLLA in toluene was investigated.^36^ The morphological evolution of PEG-*b*-PLLA structures immediately after polymerization was followed, showing shifts from 0D spheres to 1D rods and fibers to 2D lamellae. PEG-*b*-PLLA polymers quickly reach this lamellar state, which appears to be the most favorable structure. For **6c**, some rod-like structures can still be seen in the larger lamellar structures.

Finally, we mixed a selection of PEG-*b*-PLA polymers to investigate the effect on self-assembly (Figures S10–S12). Mixing **5** and **6** showed the expected stereocomplexation, where the increased affinity between the different configurations of PLA gave rise to branched networks. However, smaller branched spheres were observed compared to **3** and **4**, which might be due to the better solubility of the individual polymers. Another interesting structure was obtained with the combination of polymers **2b** and **4b**, where vesicles with branched structures on the membrane can be seen, indicating phase separation between the polymers. Besides these examples, the other combination gave similar structures to those seen with a single type of polymer. We found that changing the ratio of polymers shifts the formed structures toward the morphologies created by the pure polymer that is majorly present. For example, when polymers **5** and **6** were mixed in a 25/75 ratio, we found that half of the sample would give branched networks (25/25), while the other half would form crystal-like structures (0/50).

The differences in morphologies obtained can be explained by the design of the polymer used. Various parameters are important for the morphology during the self-assembly of block copolymers. These include the total length of the amphiphilic polymer, the ratio of the hydrophilic block vs the hydrophobic block, the repulsion between the hydrophilic chains, and the stretching of the hydrophobic chains.^37,38^ The total length and ratio of the blocks in the copolymer determine the size and morphology of the self-assembled structure. These can be tuned easily during synthesis, allowing for a myriad of structures using the same base polymer, only changing the amount of monomers used. The repulsion and stretching forces are mainly affected by environmental interactions, such as solvents and additives. The environmental conditions that affect self-assembly of PEG-*b*-PDLLA have been studied in detail elsewhere.^35^

With these polymers, we have demonstrated the critical role of tacticity in the self-assembly of amphiphilic diblock copolymers. Polymers with syndiotactic or isotactic configurations form crystalline regions, while atactic polymers create more fluidic domains due to the increased interstitial space, giving greater flexibility to the self-assembled structures (Figure 3). This phenomenon is evident in our observations: polymers **1** and **2**, which exhibit a higher degree of atacticity, form flexible polymersomes. Conversely, polymers **5** and **6**, with predominantly isotactic configurations, pack more efficiently, resulting in rigid, crystal-like assemblies. Furthermore, when these polymers are combined—either through cosolvation or by incorporating distinct regions within a single polymer, as seen with polymers **3** and **4**, stereocomplexation occurs rapidly, preempting the self-assembly process. These findings underscore the influence of polymer design on the mechanics of self-assembly, where higher isotactic content promotes rigidity in the resulting nanostructures.

**Figure 3:**
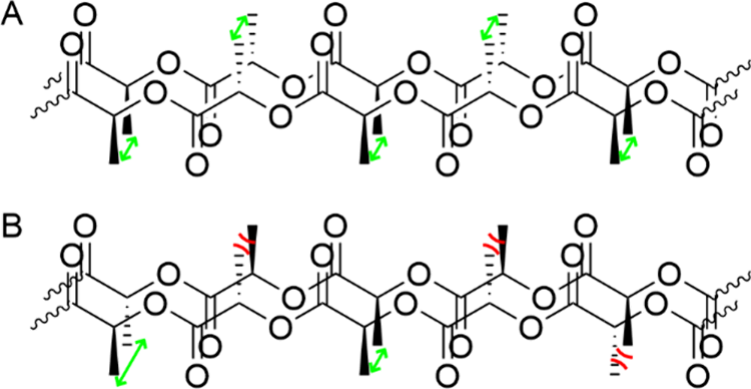
Schematic representation of PLA stacking. Isotactic poly-l-lactide is able to due to the equal distance between all methyl groups (A). Racemic poly-d,l-lactide cannot stack, as it is not possible for the methyl groups to order without steric repulsion.

In this comprehensive investigation of PEG-*b*-PLA polymers, a fundamental understanding of how polymer length, composition, and blending intricately influence nanostructures is established. The combination of atactic and tactic polymers of diverse sizes enables the assembly of a diverse spectrum of nanostructures. Through precise tailoring of tacticity and the strategic amalgamation of different chiralities, a myriad of materials ranging from polymersomes to advanced crystal-like assemblies can be crafted. We believe this knowledge is significant for the strategic design and advancement of bespoke nanoreactors, finding applications across diverse domains such as nanomedicine and materials science. The intricate control over nanostructure properties of polymers as revealed in this study stands to advance the design of nanotechnology from the molecular level.

In conclusion, we have successfully synthesized 22 different PEG-*b*-PLA polymers with varying tacticities. These include polymers made using only dd-, ll-, and dl-lactide, respectively, as well as combinations of these monomers to create di- and triblock copolymers. Various lengths of each block where made. We were able to use NMR analysis to show the difference in tacticities between these polymers. Furthermore, we have shown the effect of tacticity on the self-assembly of these PEG-*b*-PLA enantiomers. A library of well-controlled morphologies is obtained using different tacticities while the self-assembly conditions are kept constant. Atactic polymers resulted in the formation of polymersomes, while isotactic polymers consisting of pure d- and l-lactide formed diamond shaped lamellar structures. Due to stereocomplexation, the triblock copolymers with both d- and l-lactide formed larger networks. By combination of these polymers, different sizes of these nanostructures were obtained. We believe this leads to more insight into the role of chirality in self-assembly as well as greater control over the nanostructure.

## Supporting Information Available

Additional experimental details, including materials, methods, supporting figures, cryo-TEM, NMR, and GPC spectra (PDF)
