# Supplementary material for: Influence of Tacticity on the Self-Assembly of Poly(ethylene glycol)-b-poly(lactic acid) Block Copolymers
Source: ACS Macro Lett. 2025 Jan 6;14(1):101–6. doi: 10.1021/acsmacrolett.4c00758 (PMC11755779; doi:10.1021/acsmacrolett.4c00758)
Supplement: Supplementary file 1 — mz4c00758_si_001.pdf [file mz4c00758_si_001.pdf]

Supporting Information

Influence of Tacticity on Self-Assembly of Poly(Ethylene Glycol)-b-Poly(Lactic Acid) Block-Copolymers

Sjoerd J. Rijpkema‡, B. Jelle Toebes‡, Jules van Vlaenderen, Liban van Haren and Daniela A. Wilson*

Institute for Molecules and Materials, Radboud University, Heyendaalseweg 135, 6525 AJ, Nijmegen, The Netherlands

‡ These authors contributed equally

**e-mail:* d.wilson@science.ru.nl

Table of contents

1. Materials, Instrumentation and Methods 3

2. Synthesis of block co-polymers 4

3. Supporting Figures 6

4. NMR and GPC spectra of synthesized polymers 19

5. References 56

1. Materials, instrumentation and methods

All PEG polymers were obtained from Rapp Polymere. All lactide monomers were provided free of charge by Corbion. All other reagents were obtained from commercial sources and were used without purification unless otherwise stated. Solvents were dried by passing over activated alumina columns in a MBraun MB SPS800 under a nitrogen atmosphere and stored under argon. Reactions were carried without the need for an inert atmosphere unless stated otherwise, in which case the reaction was performed under a dry atmosphere of argon. Standard syringe techniques were applied for the transfer of dry solvents and air- or moisture sensitive reagents. Ultrapure water was obtained from a QPOD MilliQ system. Dialysis membranes of MWCO 12-14000 Dalton Spectra/Por were used to remove organic solvent.

Nuclear Magnetic Resonance (NMR) characterization was carried out on a Bruker AVANCE HD nanobay console with a 9.4 T Ascend magnet (400 MHz), in chloroform ($CDCl_3$). NMR spectra were recorded at 298 K unless otherwise specified. Chemical shifts are given in parts per million (ppm) with respect to tetramethylsilane (TMS, δ 0.00 ppm) as internal standard for ^1H NMR. Coupling constants are reported as J-values in Hz. Peak assignment is based on 2D gDQCOSY, ^1H-^{13}C gHSQCED, and ^1H-^{13}C gHMBC spectra. Gel permeation chromatography (GPC) equipped with PL gel 5 µm mixed D column calibrated for polystyrene (580 to 377,400 g/mol) was carried out on a Shimadzu instrument with THF as eluent using differential refractive index and UV (254 nm) detectors. Cryogenic TEM was carried out with a JEOL TEM 2100. EM Science TEM grids were glow discharged with a 208 carbon coater (Cressington). On each grid 3 µL of sample was added, blotted and immediately vitrified through freeze plunging into liquid ethane at 100% humidity using an automatic vitrification robot, FEI Vitrobot™ Mark IV (blot time 1 s, blot force 3). Samples were loaded in a 914 High tilt cryoholder (Gatan, Munich, Germany) and inserted into a JEOL Transmission Electron Microscope 2100 (Japan) at 200 kV. Images were taken with a 4096 x 4096 pixel CCD camera (Gatan). The average dimensions and membrane thickness of each sample were obtained from different regions (images) and analyzed with plot profile tools of Fiji. Malvern Zetasizer nano S was used for dynamic light scattering (DLS) measurements equipped with He-Ne laser of wavelength 633 nm. All images analysis was carried out using ImageJ, available in a public domain http://fiji.sc/ [1].

2. Synthesis of polymers

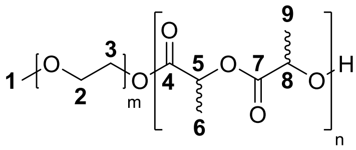

2.1 α-methoxy poly(ethylene glycol)-b-polylactide block copolymers using LLA and DLA.

Poly(ethylene glycol)-*b*-polylactide (PEG-*b*-PLA) was synthesized by ring opening polymerization (ROP). Methoxy-PEG-OH macroinitiator (194 mg for 1K or 397 mg for 2K, 0.2 mmol, 1 eq.) was mixed with lactide (1.30-2.60 g, 9.0-18.0 mmol, 45-90 eq.). For each polymer the amount of lactide was adjusted to obtain a PLA chain length of 45 or 90. First, the reagents were dried by addition of dry toluene and removing the solvent under reduced pressure. Then, dry toluene (15 mL) was added to the dried material under argon. Subsequently, $Sn(oct)_2$ (4.3 µL, 0.013 mmol, 0.065 eq.) was added and the mixture was degassed for 30 minutes with argon. The reaction then refluxed for 16 h at 111 °C. Afterwards, the mixture was concentrated under reduced pressure and subsequently precipitated in ice cold diethyl ether (2x). The polymer was then dissolved in 1,4-dioxane (5 ml) and lyophilized to yield a white powder (21-97% yield). **^{1}H NMR** (400 MHz, $CDCl_3$) δ 5.19 (q, *J* = 7.11 Hz, 2nH, 5-C*H* + 8-C*H*), 3.64 (br s, 4mH, 2-CH_2 + 3-CH_2), 3.38 (s, 3H, 1-CH_3), 1.58 (d, *J* = 7.11 Hz, 6nH, 6-CH_3 + 9-CH_3). **^{13}C NMR** (101 MHz, $CDCl_3$) δ 169.6 (4-*C* + 7-*C*), 71.1 (2-*C*H_2 + 3-*C*H_2), 68.8 (5-*C*H + 8-*C*H), 59.0 (1-*C*H_3), 16.8 (6-*C*H_3 + 9-*C*H_3)).

2.2 α-methoxy poly(ethylene glycol)-b-polylactide block copolymers using DLLA

Poly(ethylene glycol)-*b*-polylactide (PEG-*b*-PLA) was synthesized by ring opening polymerization (ROP). Methoxy-PEG-OH macroinitiator (194 mg 1K or 397 mg 2K, 0.2 mmol, 1 eq.) was mixed with lactide (1.30-2.60 g, 9.0-18.0 mmol, 45-90 eq.). For each polymer the amount of lactide was adjusted to obtain a PLA chain length of 45 or 90. First, the reagents were dried by addition of dry toluene and removing the solvent under reduced pressure. Then, dry DCM (15 mL) was added to the dried material under argon. Subsequently, DBU (15 µL, 0.1 mmol, 0.5 eq.) was added to the mixture. The reaction then stirred for 4 h at 30 °C. Afterwards, the mixture was washed with 1M $KHSO_4$ (3x), concentrated under reduced pressure and subsequently precipitated in ice cold diethyl ether (2x). The polymer was then dissolved in 1,4-dioxane (5 ml) and lyophilized to yield a white powder (47-87% yield). **^{1}H NMR** (400 MHz, $CDCl_3$) δ 5.30−5.13 (m, 2nH, 5-C*H* + 8-C*H*), 3.64 (br s, 4mH, 2-CH_2 + 3-CH_2), 3.38 (s, 3H, 1-CH_3), 1.66−1.50 (m, 6nH, 6-CH_3 + 9-CH_3). **^{13}C NMR** (101 MHz, $CDCl_3$) δ 169.6 (4-*C* + 7-*C*), 71.1 (2-*C*H_2 + 3-*C*H_2), 68.6 (5-*C*H + 8-*C*H), 59.0 (1-*C*H_3), 16.3 (6-*C*H_3 + 9-*C*H_3)).

2.3 Self-assembly of PEG-*b*-PLA diblock-copolymers

In total, 10 mg PEG-*b*-PLA polymer was dissolved in a mixture of THF and 1,4-dioxane (1 mL, 4:1 v/v) in a glass vial with stirring bar. In case of a mixture, 5 mg of each polymer was used. After dissolving the polymer for 0.5 h at 21 °C while stirring, a syringe pump equipped with a syringe and a needle was used to deliver ultrapure water with a rate of 1 mL/h for 0.5 h via a rubber septum, while vigorously stirring the mixture (900 rpm). Upon finishing the water addition, the suspension was transferred to a pre-hydrated membrane (Spectra/Por, molecular weight cut-off: 12-14 kDa) and dialyzed against 1L of MilliQ water for 24 hours in a fridge at 4 °C, with a solution change after 1 h. Samples were stored in the fridge at 4 °C.

3. Supporting Figures

Table S1: Overview of PEG-*b*-PLA block copolymer compositions.

	Polymer	Polymer composition (NMR)	PDI (GPC)	Yield (%)
**1a**	PEG_{22}-*b*-$PDLLA_{45}$	PEG_{22}-*b*-$PDLLA_{47}$	1.22	73
**1b**	PEG_{44}-*b*-$PDLLA_{45}$	PEG_{22}-*b*-$PDLLA_{43}$	1.05	87
**1c**	PEG_{22}-*b*-$PDLLA_{90}$	PEG_{22}-*b*-$PDLLA_{91}$	1.12	47
**1d**	PEG_{44}-*b*-$PDLLA_{90}$	PEG_{44}-*b*-$PDLLA_{98}$	1.09	76
**2a**	PEG_{22}-*b*-$P(D+L)LA_{45}$	PEG_{22}-*b*-$P(D+L)LA_{45}$	1.10	69
**2b**	PEG_{44}-*b*-$P(D+L)LA_{45}$	PEG_{44}-*b*-$P(D+L)LA_{35}$	1.09	70
**2c**	PEG_{22}-*b*-$P(D+L)LA_{90}$	PEG_{22}-*b*-$P(D+L)LA_{94}$	1.27	61
**2d**	PEG_{44}-*b*-$P(D+L)LA_{90}$	PEG_{44}-*b*-$P(D+L)LA_{90}$	1.16	21
**3a**	PEG_{22}-*b*-$PDLA_{22}$-*b*-$PLLA_{22}$	PEG_{22}-*b*-$PDLA_{26}$-*b*-$PLLA_{23}$	1.06	71
**3b**	PEG_{44}-*b*-$PDLA_{22}$-*b*-$PLLA_{22}$	PEG_{44}-*b*-$PDLA_{18}$-*b*-$PLLA_{24}$	1.08	34
**3c**	PEG_{22}-*b*-$PDLA_{45}$-*b*-$PLLA_{45}$	PEG_{22}-*b*-$PDLA_{52}$-*b*-$PLLA_{52}$	1.11	75
**3d**	PEG_{44}-*b*-$PDLA_{45}$-*b*-$PLLA_{45}$	PEG_{44}-*b*-$PDLA_{45}$-*b*-$PLLA_{33}$	1.12	81
**4a**	PEG_{22}-*b*-$PLLA_{22}$-*b*-$PDLA_{22}$	PEG_{22}-*b*-$PLLA_{26}$-*b*-$PDLA_{22}$	1.09	97
**4b**	PEG_{44}-*b*-$PLLA_{22}$-*b*-$PDLA_{22}$	PEG_{44}-*b*-$PLLA_{18}$-*b*-$PDLA_{17}$	1.09	52
**4c**	PEG_{22}-*b*-$PLLA_{45}$-*b*-$PDLA_{45}$	PEG_{22}-*b*-$PLLA_{45}$-*b*-$PDLA_{41}$	1.19	73
**4d**	PEG_{44}-*b*-$PLLA_{45}$-*b*-$PDLA_{45}$	PEG_{44}-*b*-$PLLA_{47}$-*b*-$PDLA_{30}$	1.16	81
**5a**	PEG_{22}-*b*-$PLLA_{45}$	PEG_{22}-*b*-$PLLA_{54}$	1.24	87
**5b**	PEG_{22}-*b*-$PLLA_{90}$	PEG_{22}-*b*-$PLLA_{92}$	1.35	93
**5c**	PEG_{44}-*b*-$PLLA_{90}$	PEG_{44}-*b*-$PLLA_{85}$	1.11	96
**6a**	PEG_{22}-*b*-$PDLA_{45}$	PEG_{22}-*b*-$PDLA_{48}$	1.51	85
**6b**	PEG_{22}-*b*-$PDLA_{90}$	PEG_{22}-*b*-$PDLA_{95}$	1.26	92
**6c**	PEG_{44}-*b*-$PDLA_{90}$	PEG_{44}-*b*-$PDLA_{91}$	1.14	91

Note: GPC of polymers 3 and 4 were measured at a lower concentration of 3 mg/mL to avoid stereocomplexation.

Table S2: Overview self-assembly PEG-PDLLA (Polymer 1).

Polymer	Morphology	Size (nm)	PDI
22-45	Spherical PS	344	0.09
44-45	Micelles	18	0.37
22-90	Aggregates + LCVs	457	0.43
44-90	Micelles	65	0.1
22-45 + 44-45	Micelles	24	0.24
22-45 + 22-90	Irregular PS	~1524	1.0
22-45 + 44-90	Spherical PS	85	0.04
22-90 + 44-90	Spherical PS	304	0.11

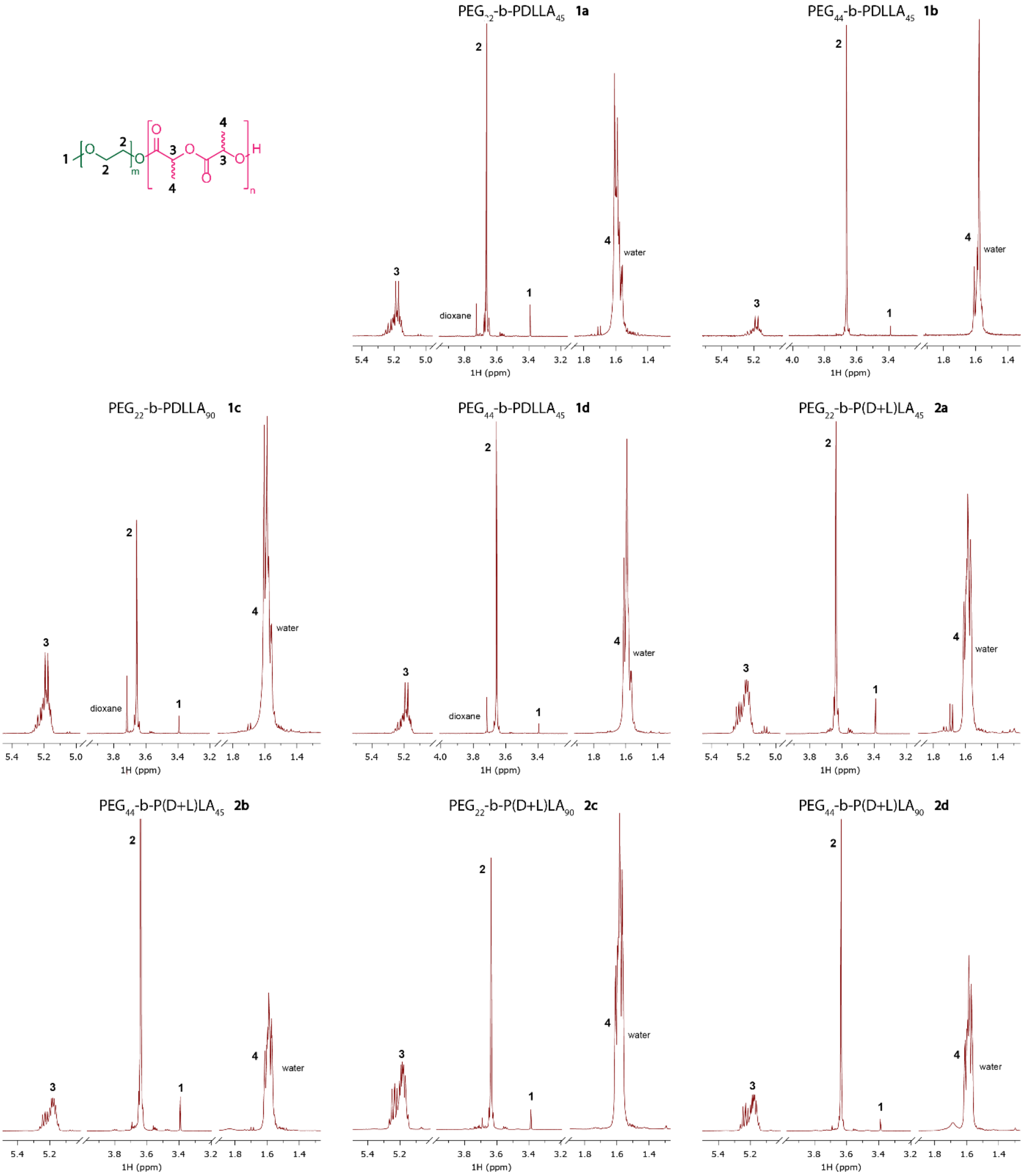

Figure S1. ^{1}H NMR spectra of PEG-*b*-PDLLA **1** and PEG-*b*-P(D+L)LA **2** polymers. Compositions are calculated by integrating peaks 2 and 3 relatively to peak 1.

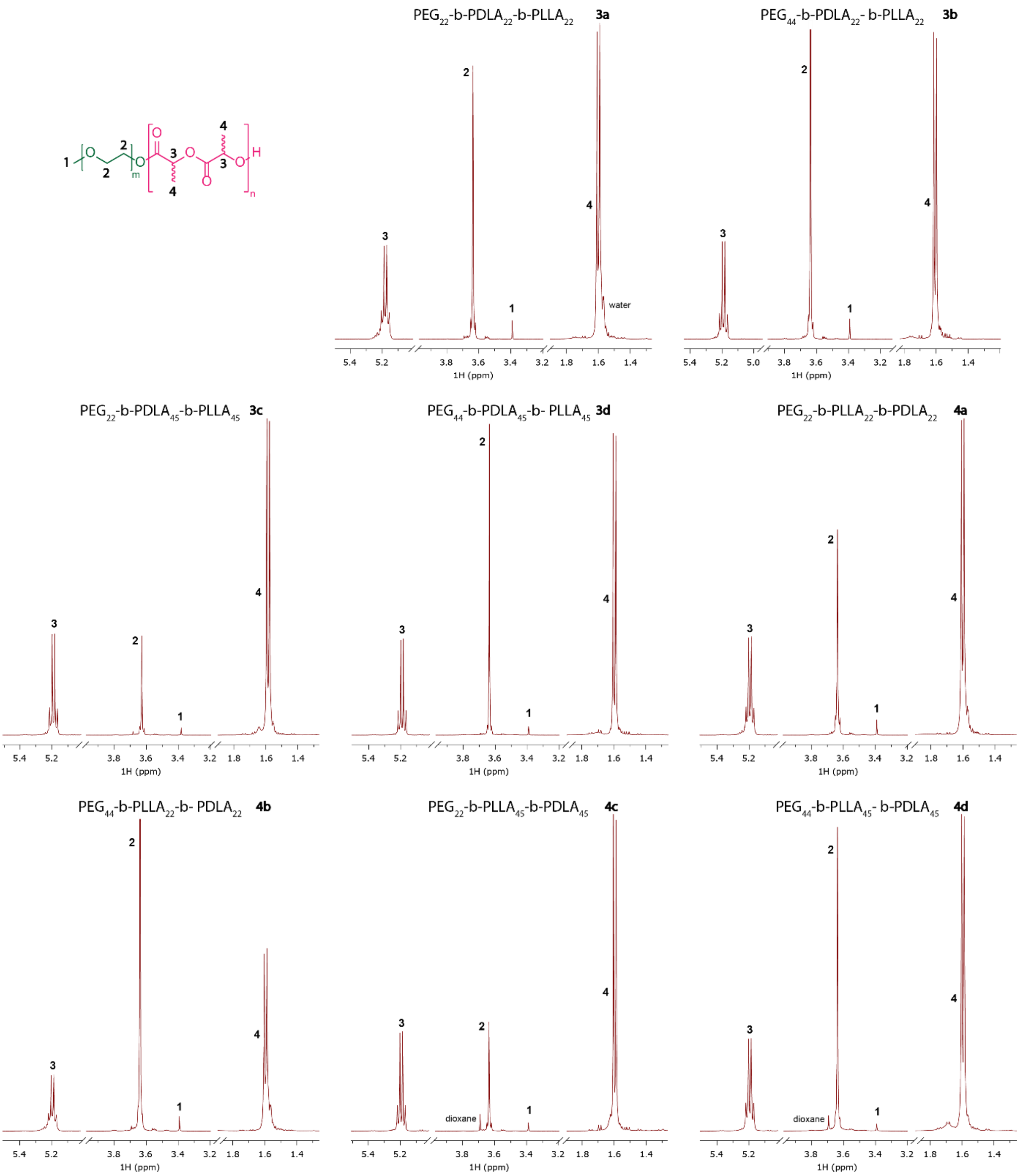

Figure S2. ^{1}H NMR spectra of PEG-*b*-PDLA-*b*-PLLA **3** and PEG-*b*-PLLA-*b*-PDLA **4** polymers. Compositions are calculated by integrating peaks 2 and 3 relatively to peak 1.

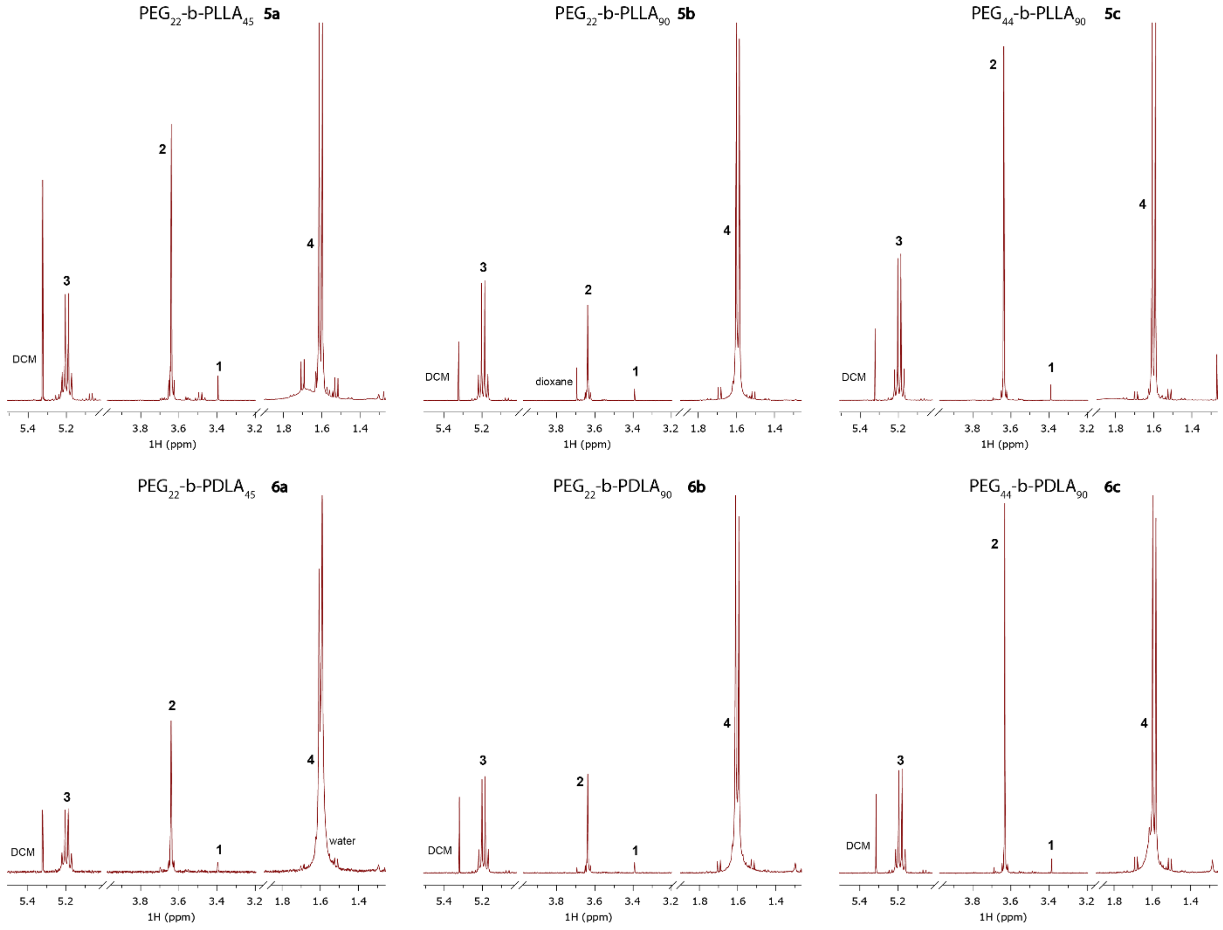

Figure S3. ^{1}H NMR spectra of PEG-*b*-PLLA **5** and PEG-*b*-PDLA **6** polymers. Compositions are calculated by integrating peaks 2 and 3 relatively to peak 1.

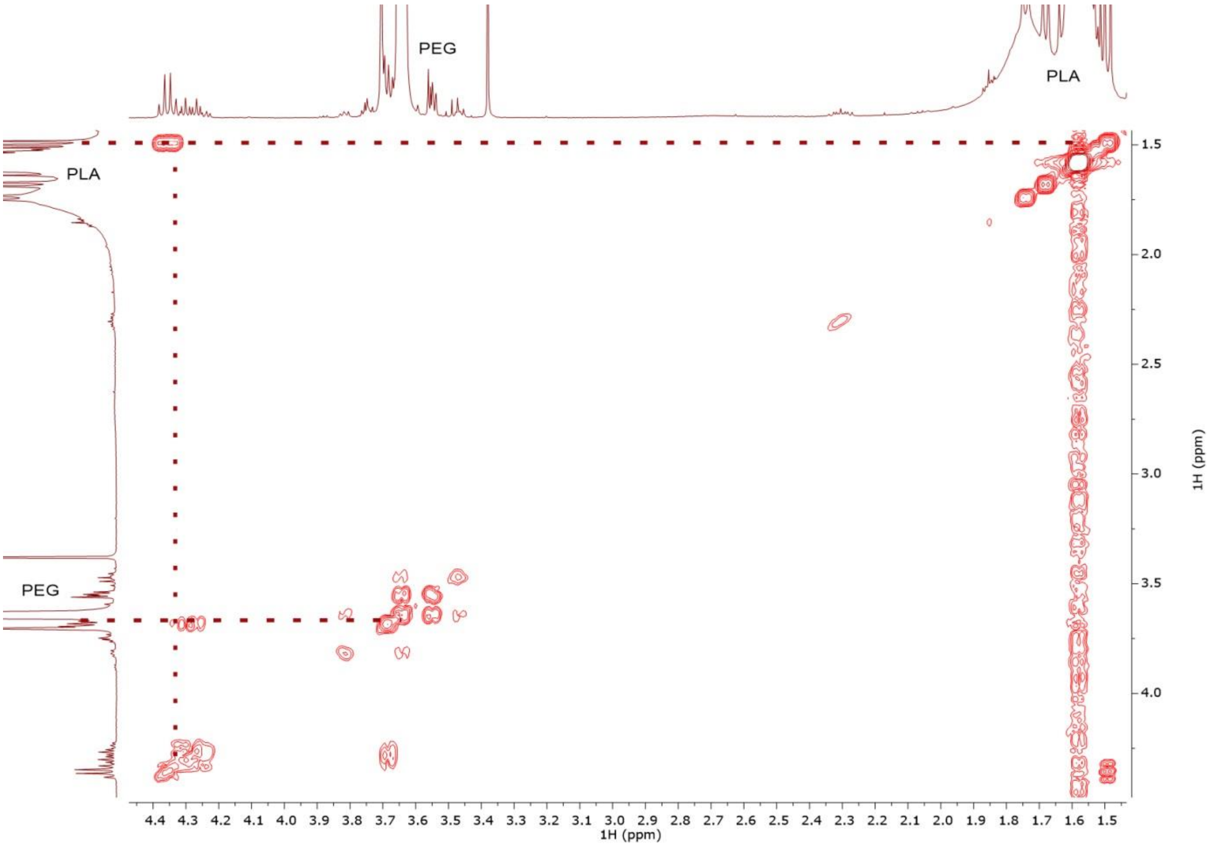

Figure S4. ^{1}H-^{1}H COSY NMR spectra of PEG$_{22}$-*b*-PLLA$_{45}$ **5a** polymers. The red dotted lines indicating the cross-peaks between the multiplet and the PEG and PLA bulk signals.

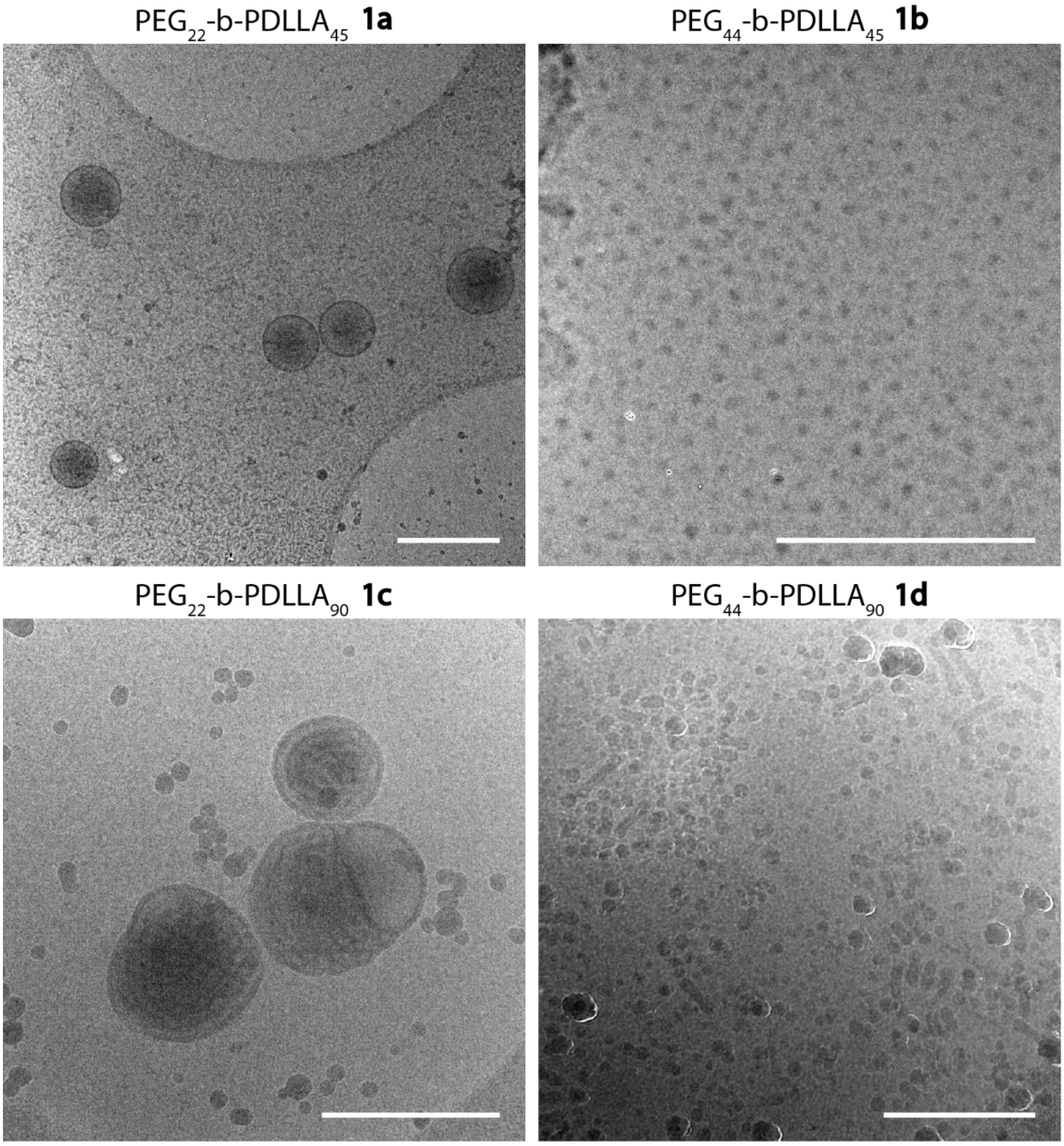

Figure S5. Cryo-TEM images of PEG-*b*-PDLLA **1** polymer self-assemblies. Scale bar: 500 nm.

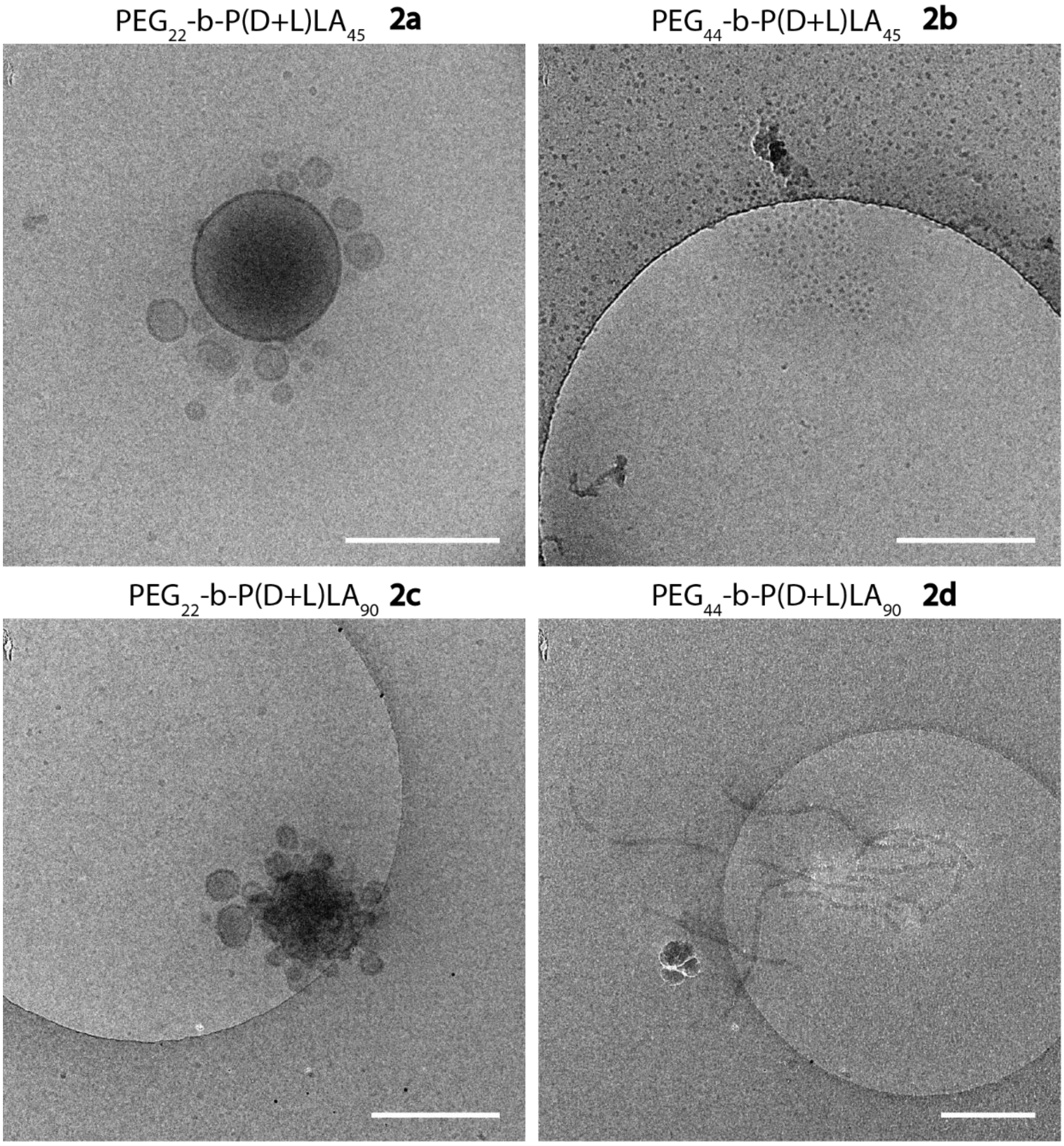

Figure S6. Cryo-TEM images of PEG-*b*-P(D+L)LA **2** polymer self-assemblies. Scale bar: 500 nm.

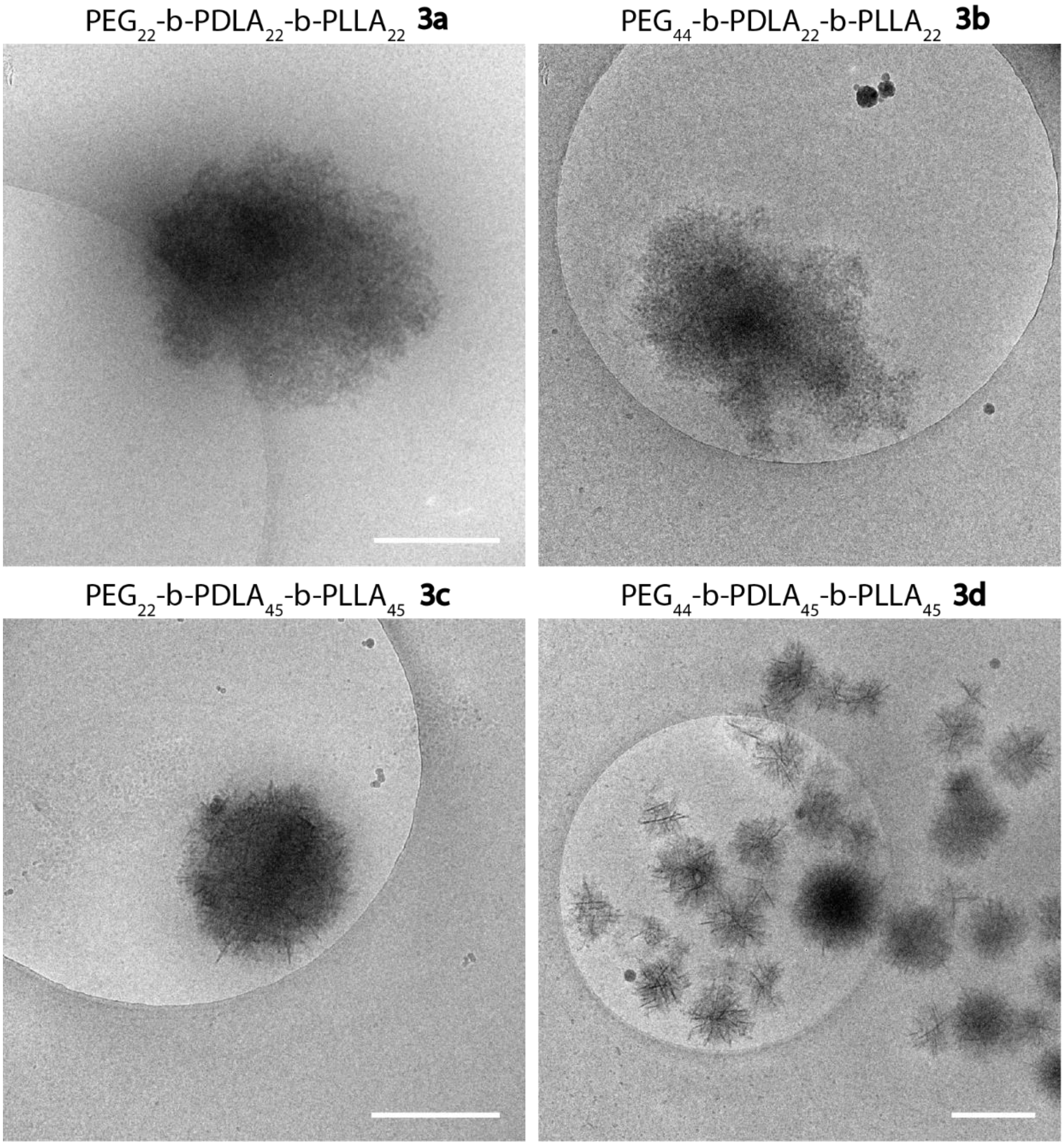

Figure S7. Cryo-TEM images of PEG-*b*-PDLA-*b*-PLLA **3** polymer self-assemblies. Scale bar: 500 nm.

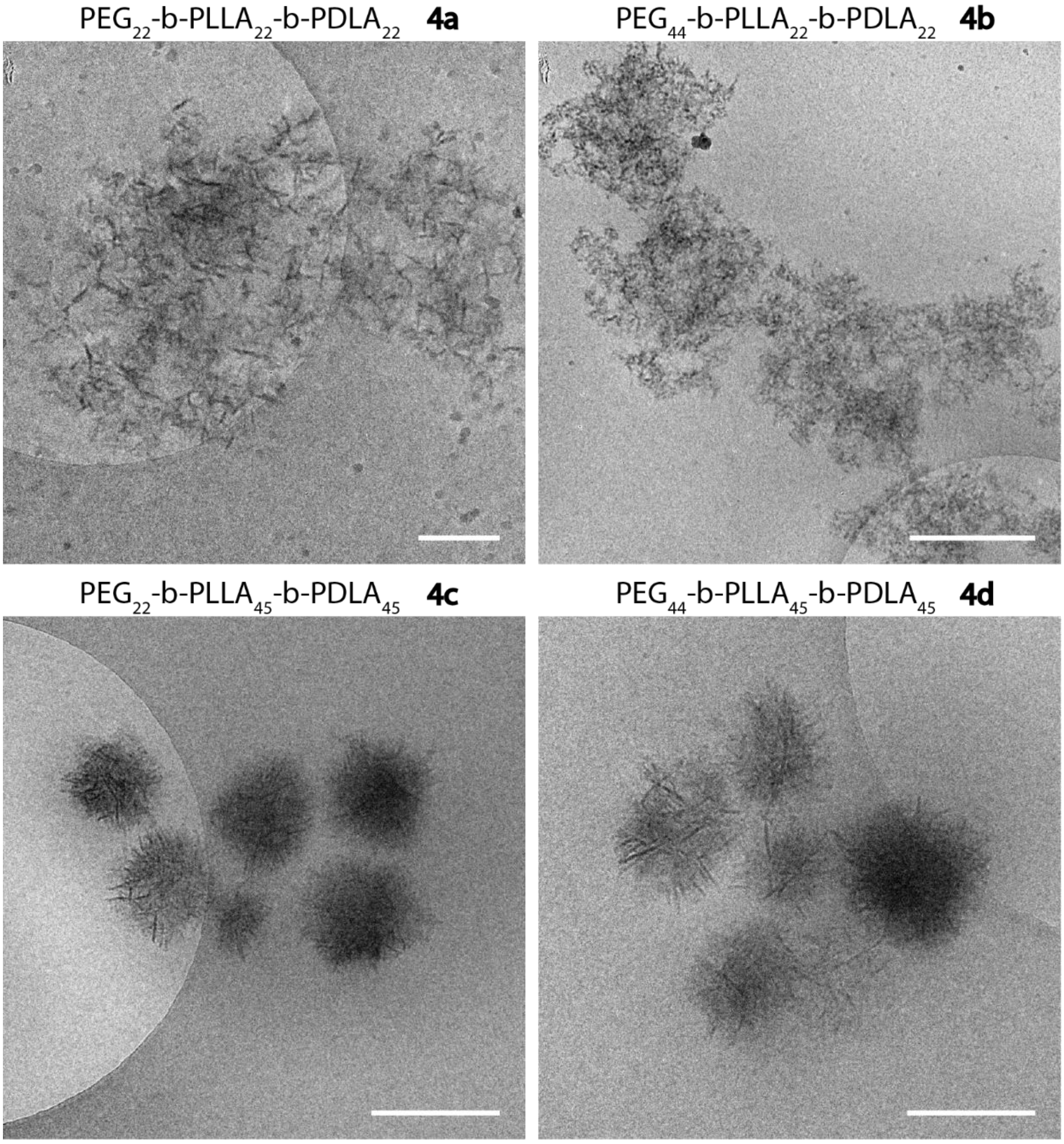

Figure S8. Cryo-TEM images of PEG-*b*-PLLA-*b*-PDLA **4** polymer self-assemblies. Scale bar: 500 nm.

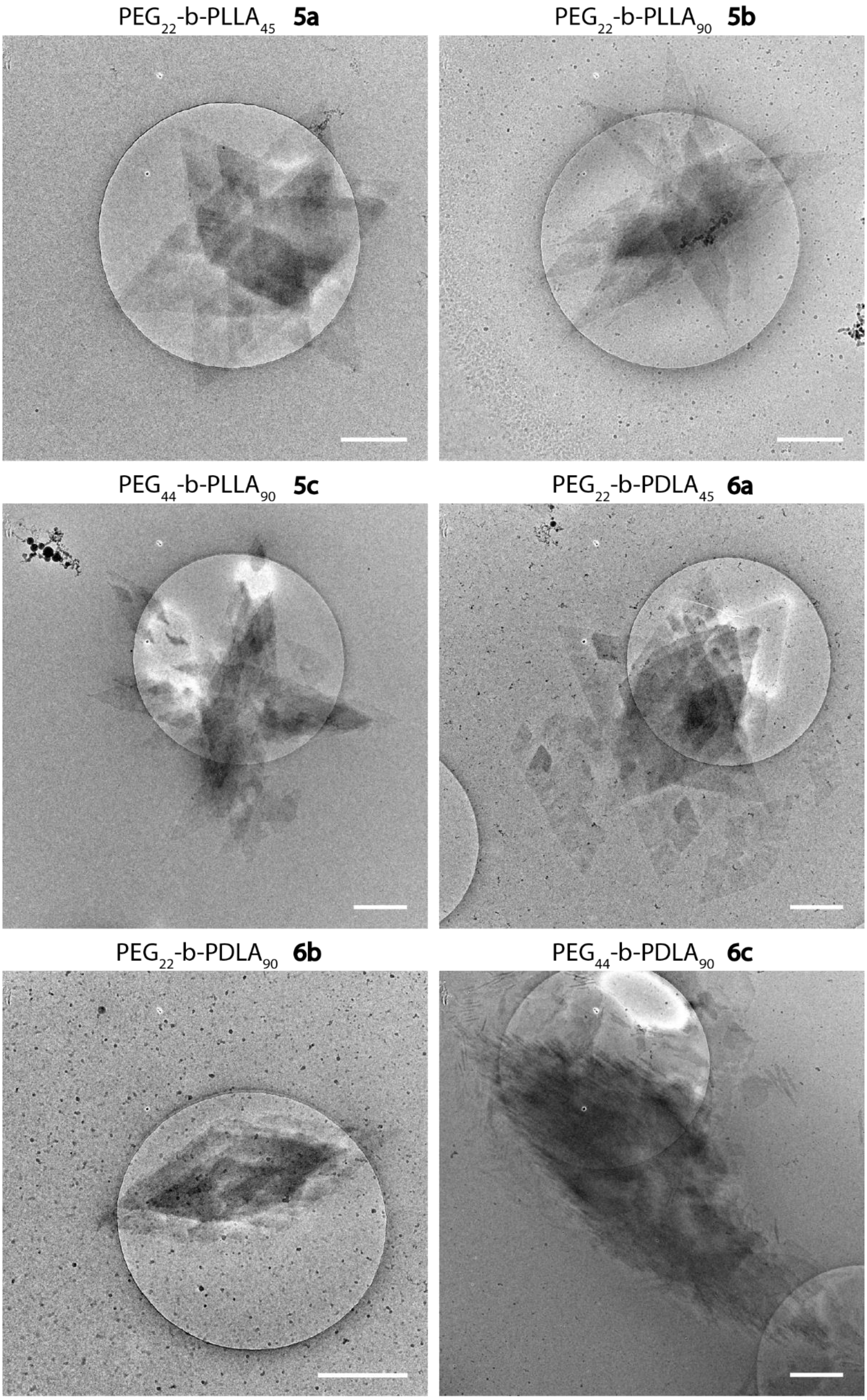

Figure S9. Cryo-TEM images of PEG-*b*-PLLA **5** and PEG-*b*-PDLA **6** polymer self-assemblies. Scale bar: 500 nm.

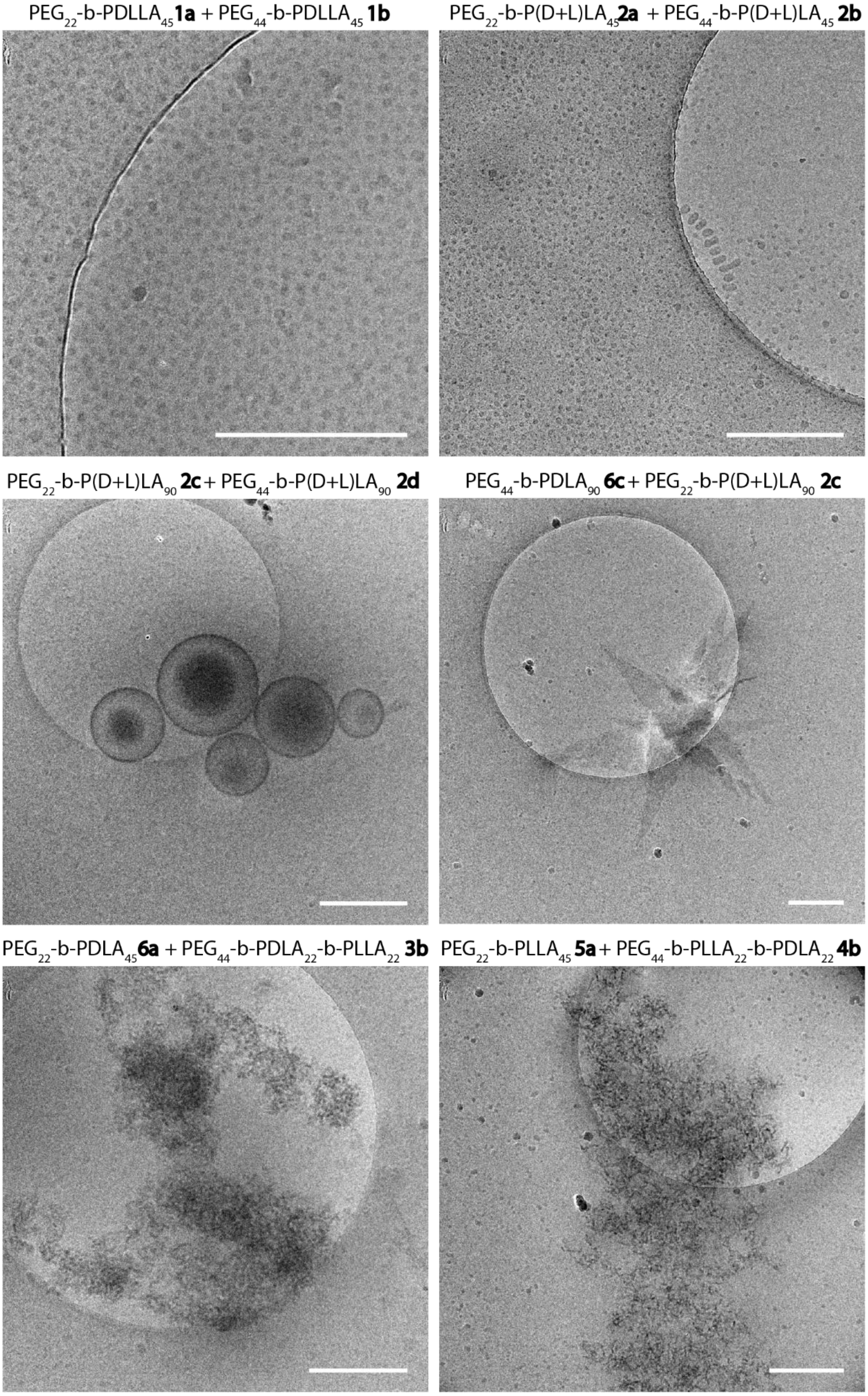

Figure S10. Cryo-TEM images of mixtures of PEG-*b*-PLA polymer self-assemblies. Scale bar: 500 nm.

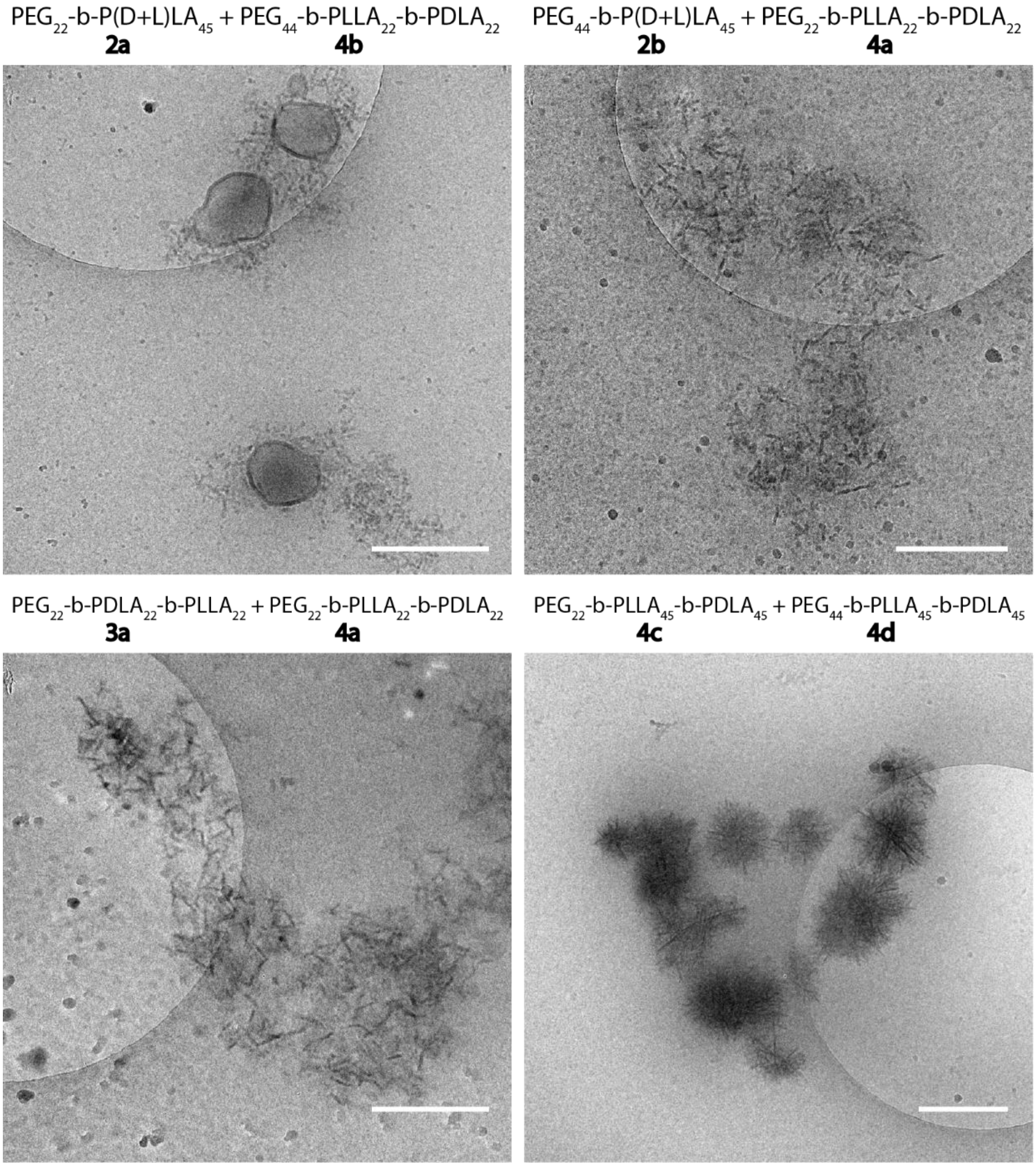

Figure S11. Cryo-TEM images of mixtures of PEG-*b*-PLA polymer self-assemblies. Scale bar: 500 nm.

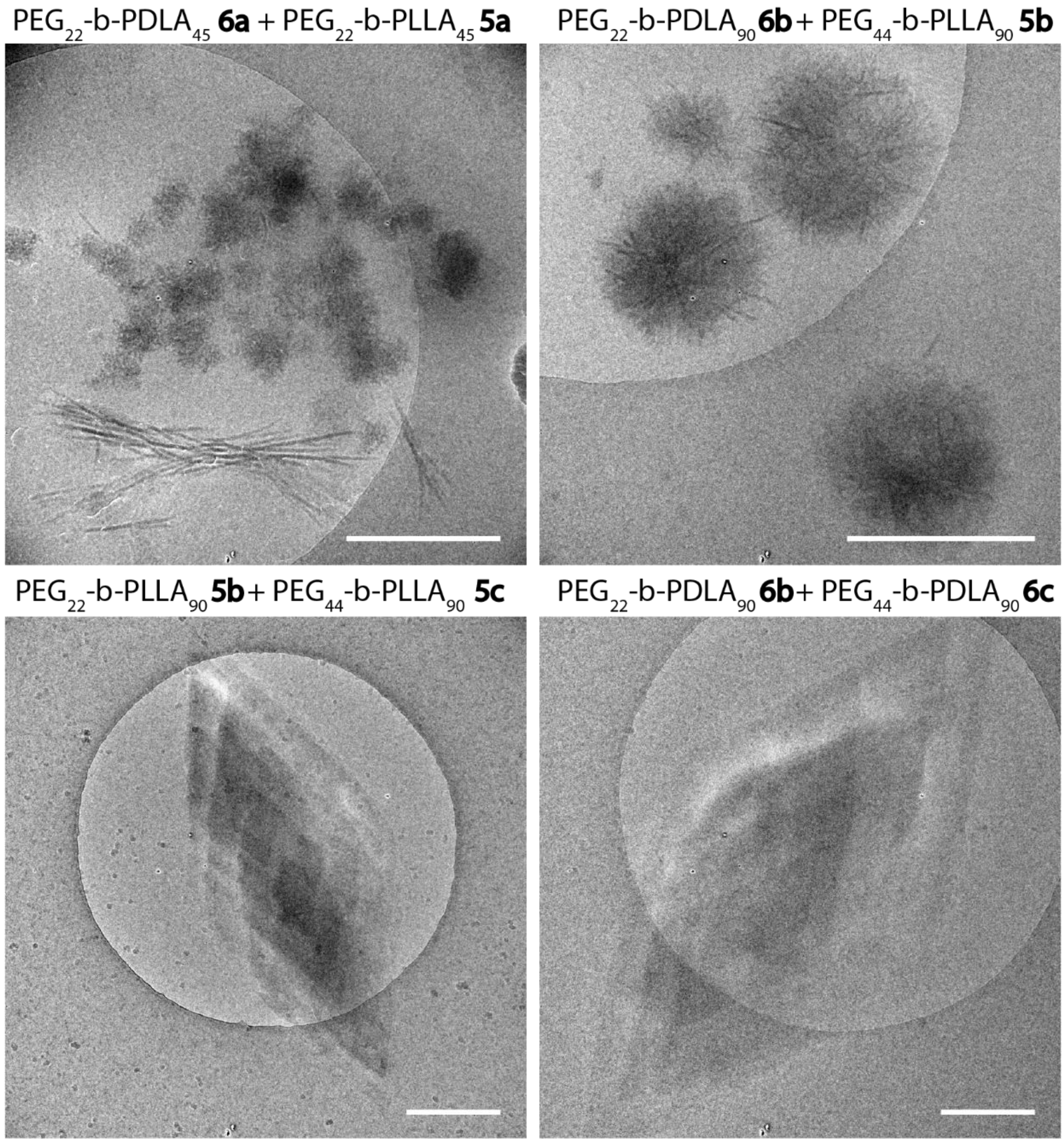

Figure S12. Cryo-TEM images of mixtures of PEG-*b*-PLA polymer self-assemblies. Scale bar: 500 nm.

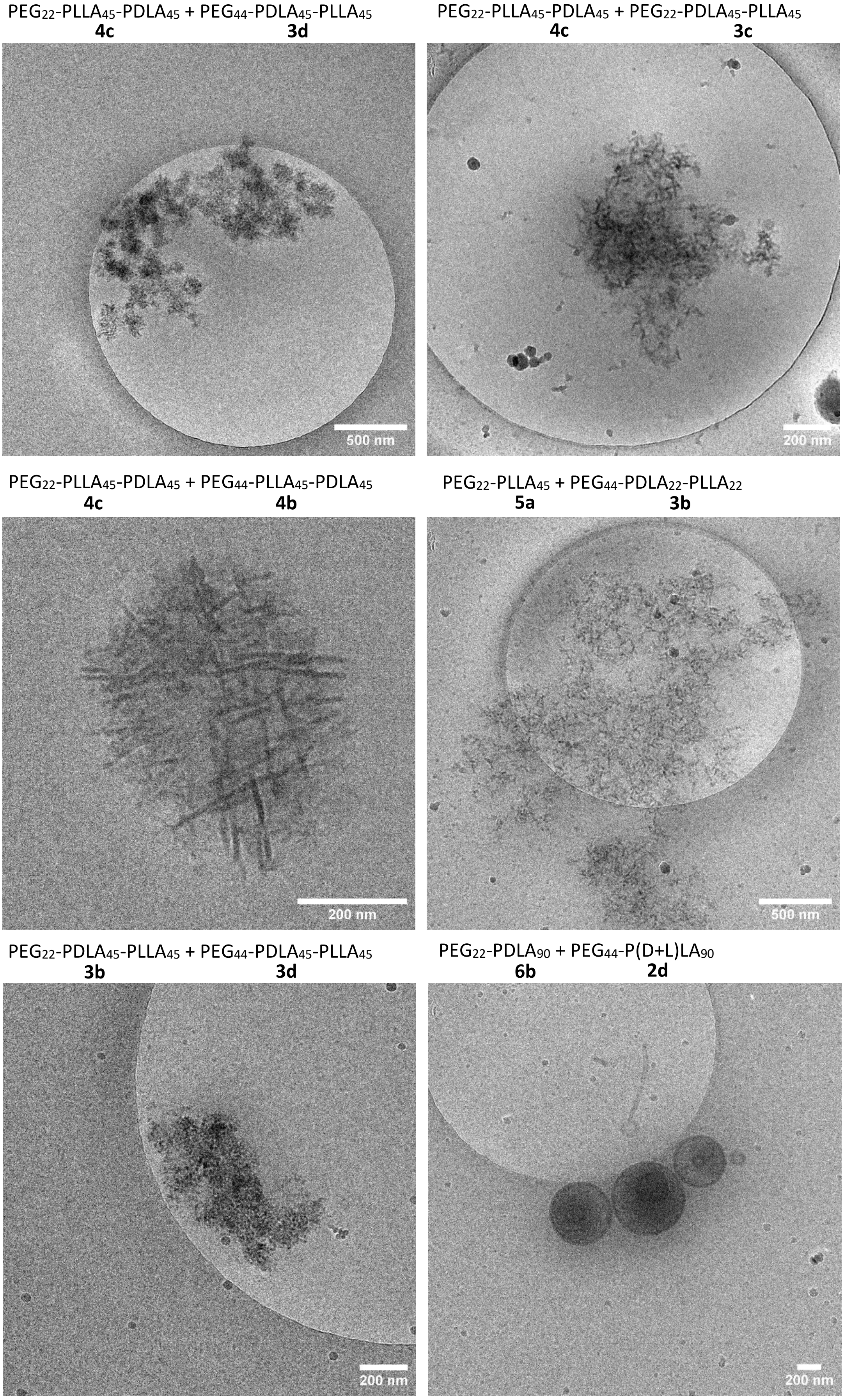

Figure S13. Cryo-TEM images of mixtures of PEG-*b*-PLA polymer self-assemblies.

4. NMR and GPC spectra of polymers

PEG$_{22}$-*b*-PDLLA$_{45}$ **1a**

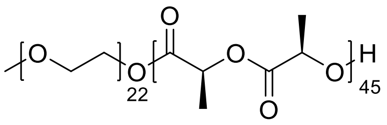

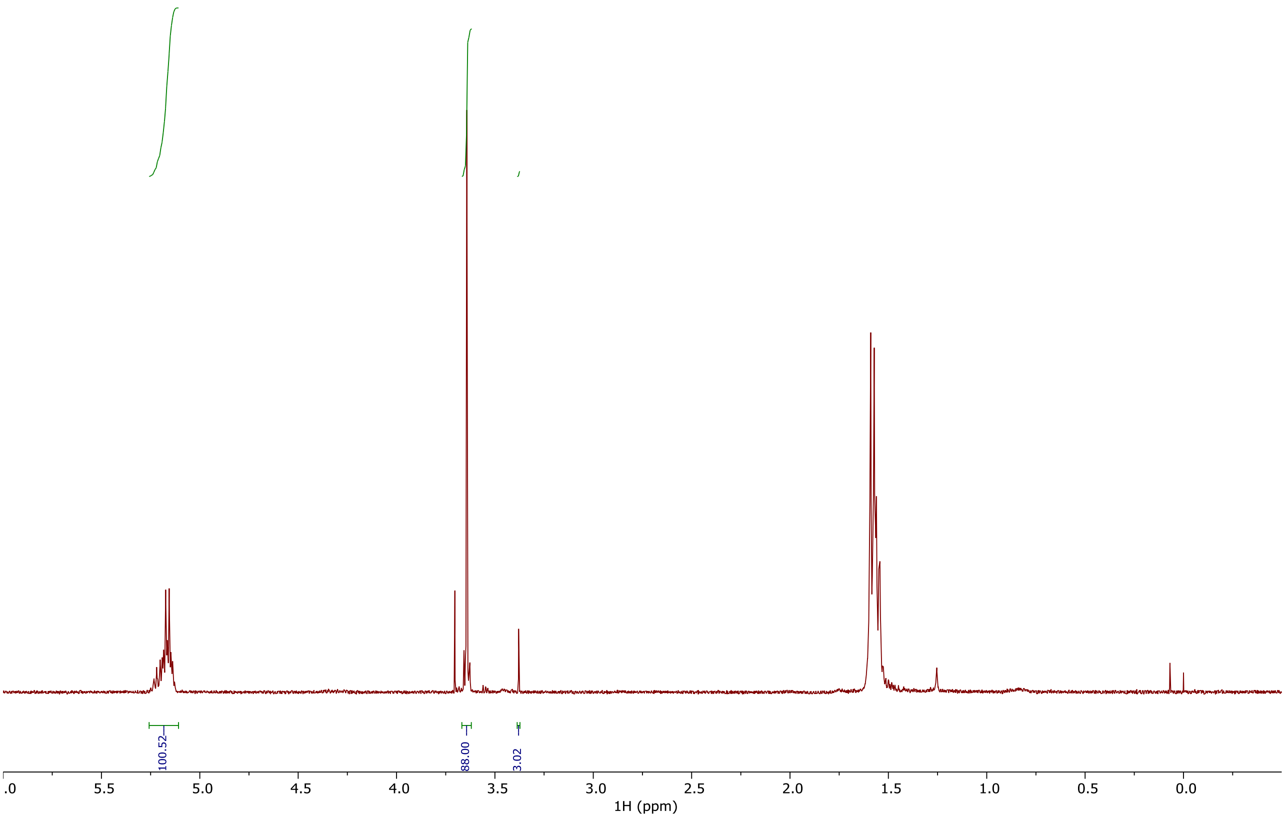

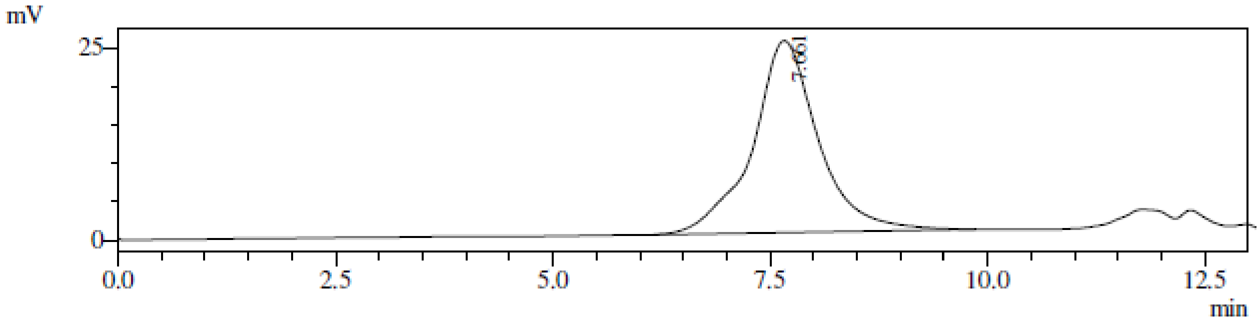

Mw/Mn	1.22095

PEG$_{44}$-*b*-PDLLA$_{45}$ **1b**

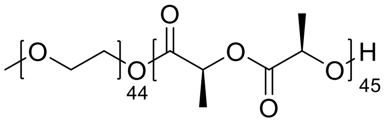

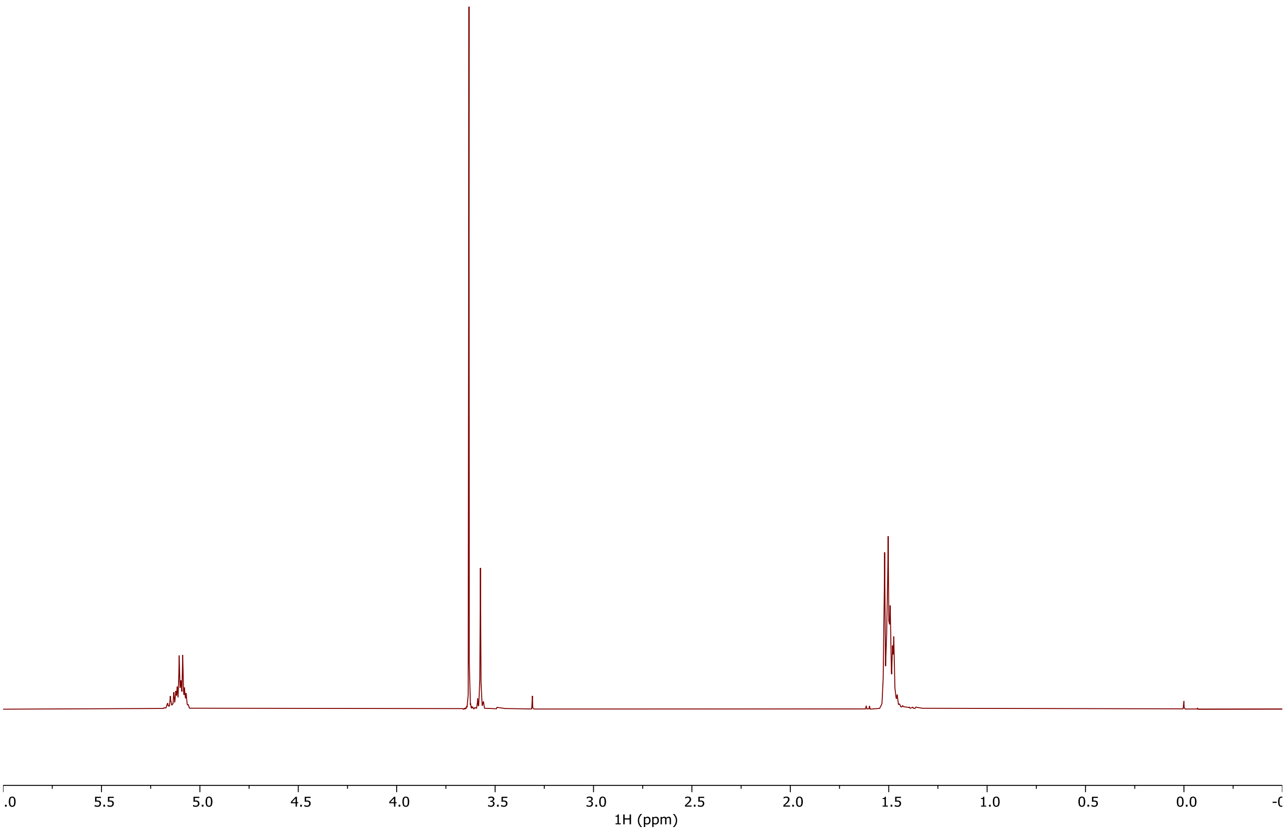

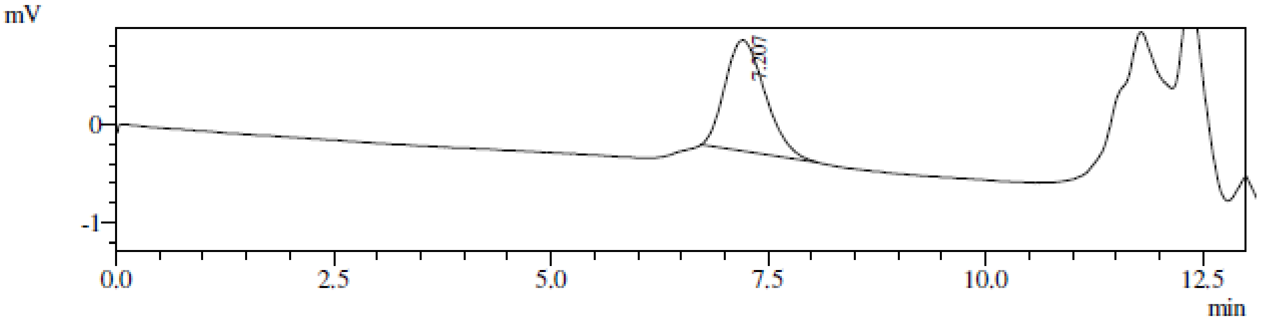

Mw/Mn 1.04850

PEG$_{22}$-*b*-PDLLA$_{90}$ **1c**

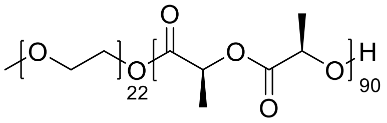

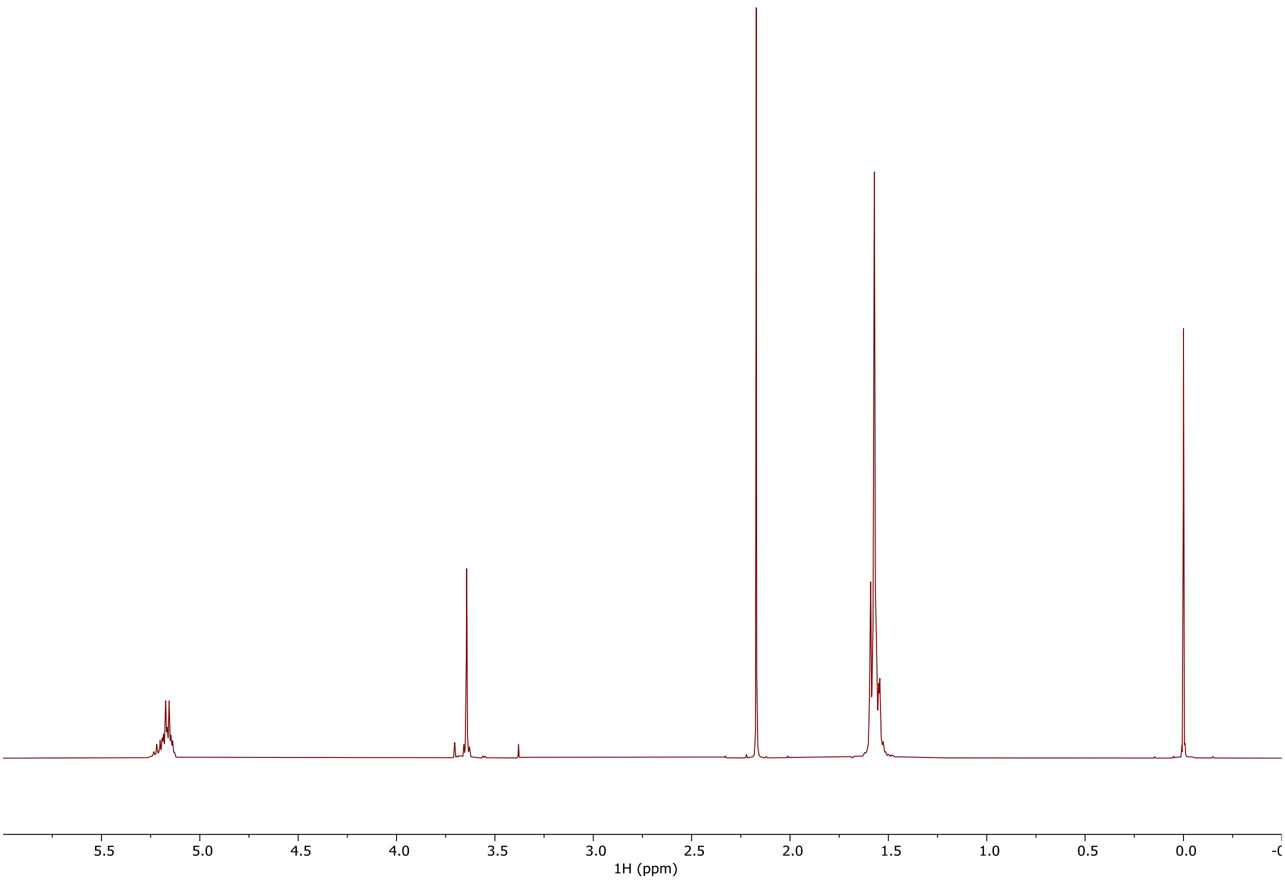

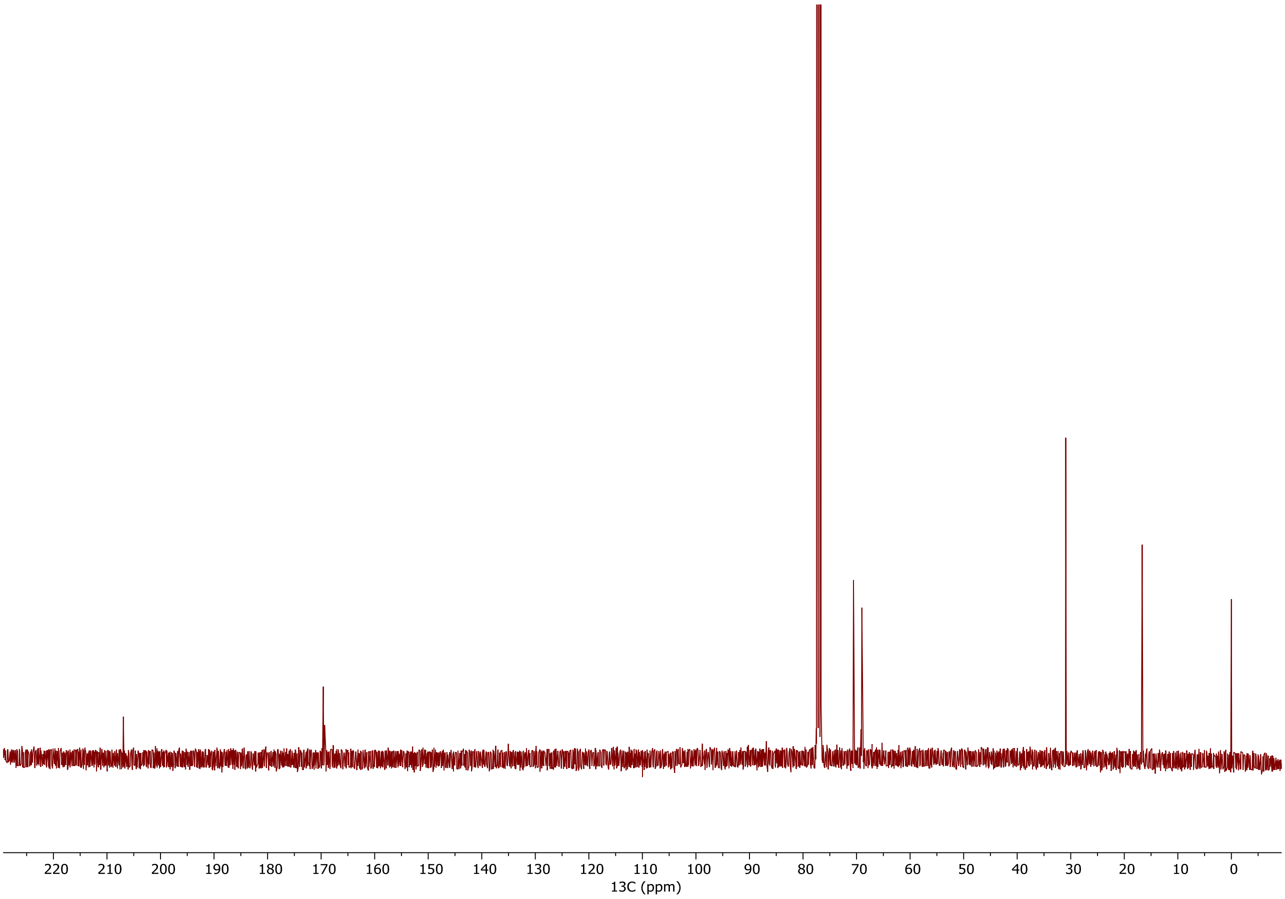
220
210
200
190
180
170
160
150
140
130
120
110
100
90
80
70
60
50
40
30
20
10
0
13C (ppm)

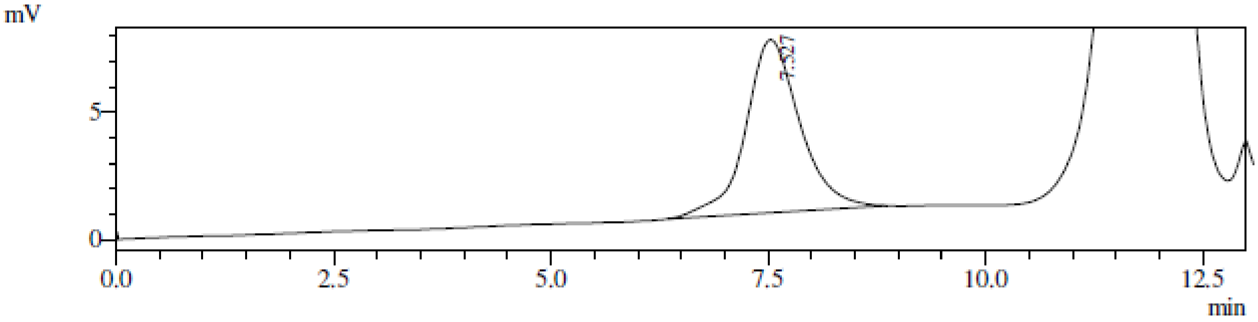
mV
5
0
7.527
0.0
2.5
5.0
7.5
10.0
12.5
min

Mw/Mn 1.11554

PEG$_{44}$-*b*-PDLLA$_{90}$ **1d**

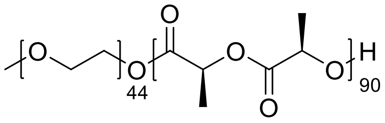

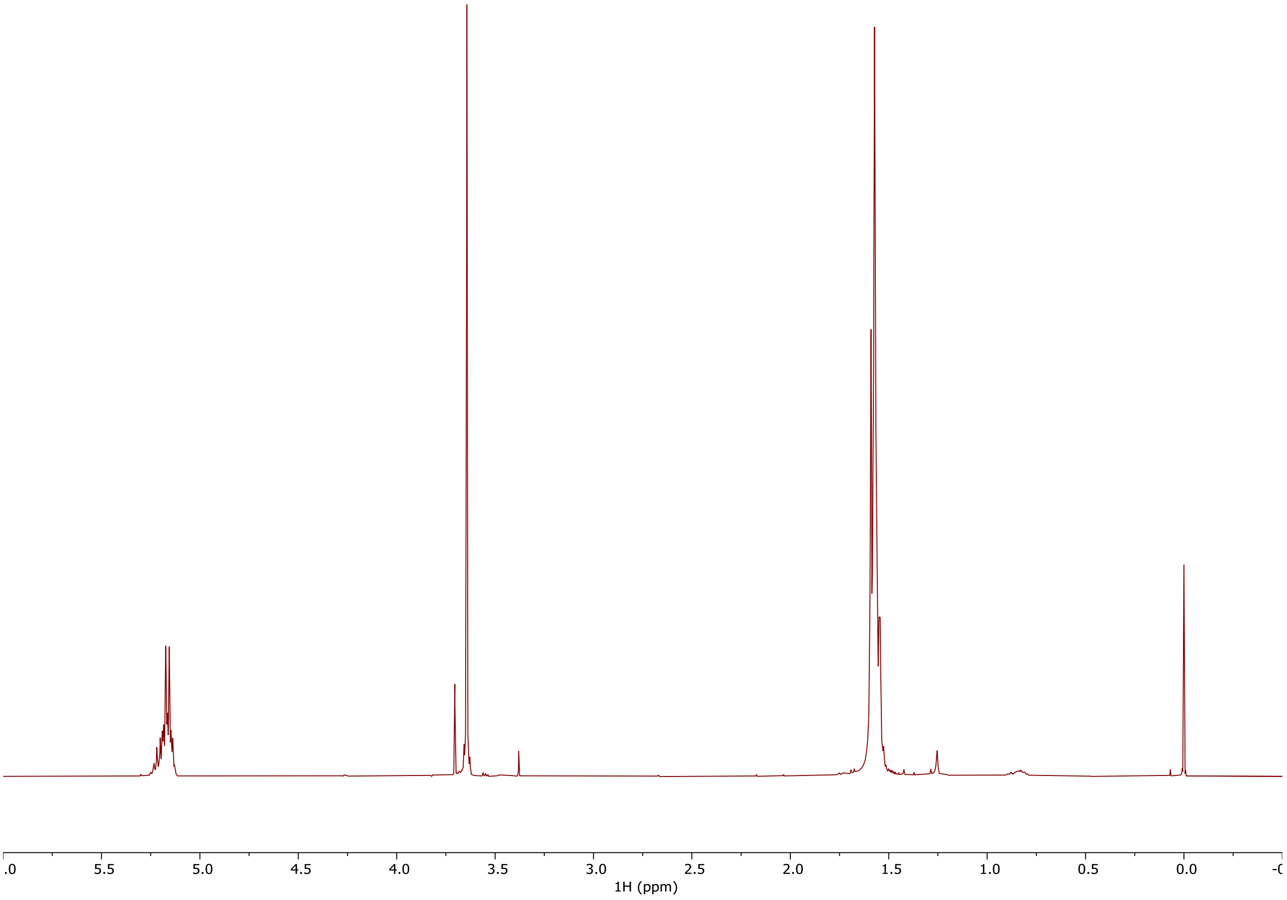

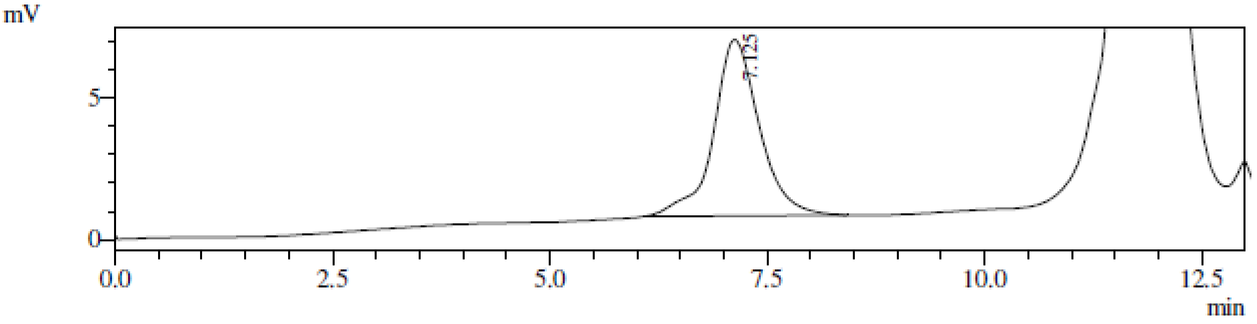

Mw/Mn 1.08972

PEG$_{22}$-*b*-P(D+L)LA$_{45}$ **2a**

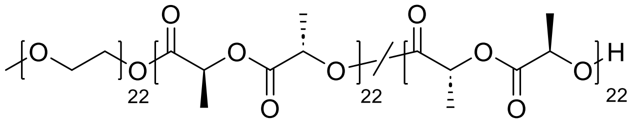

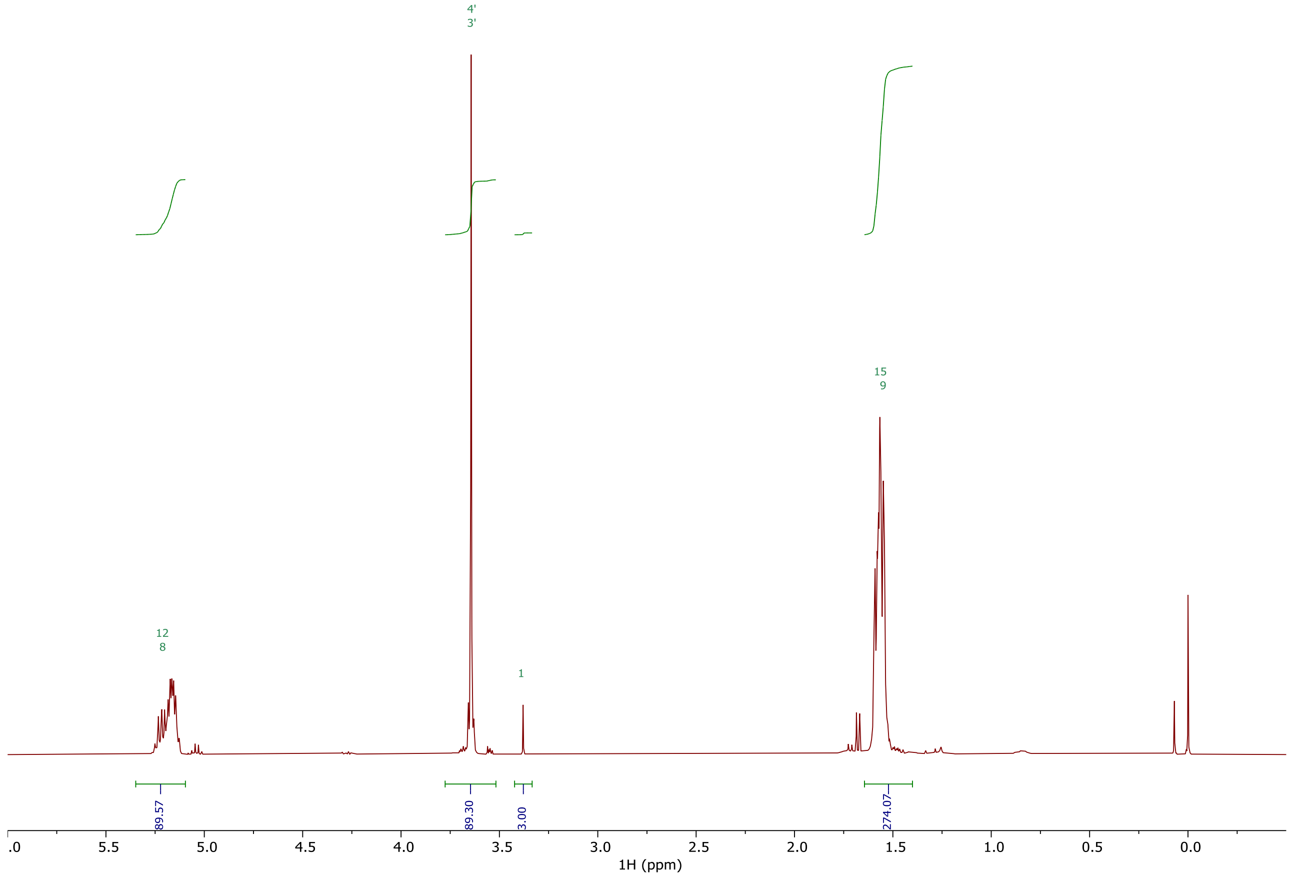

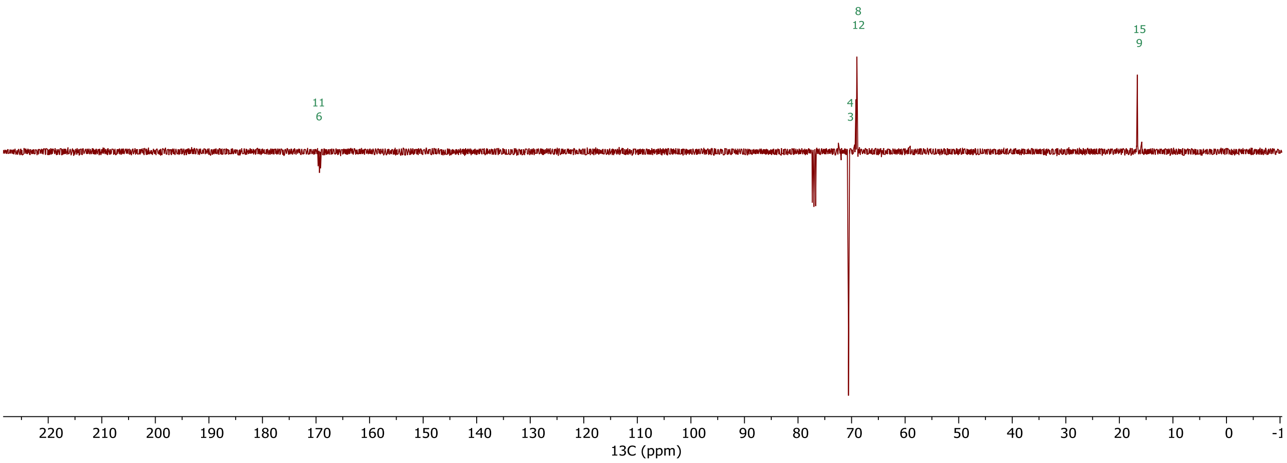

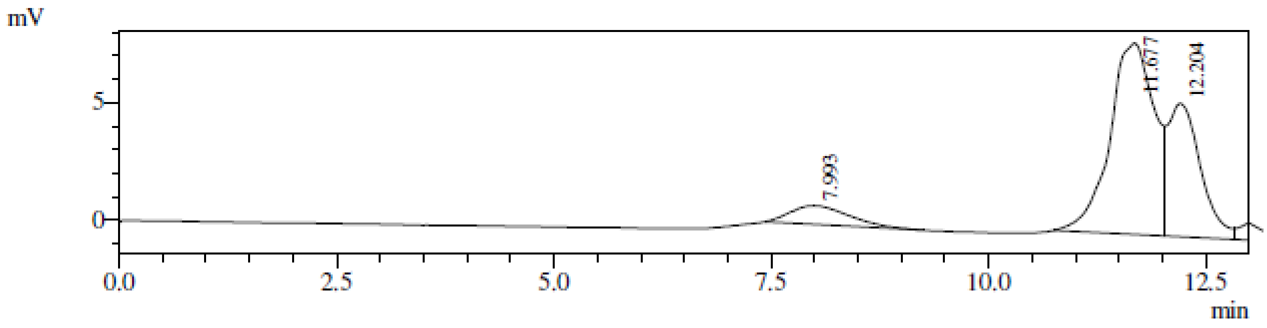

Mw/Mn 1.10381

PEG$_{44}$-*b*-P(D+L)LA$_{45}$ **2b**

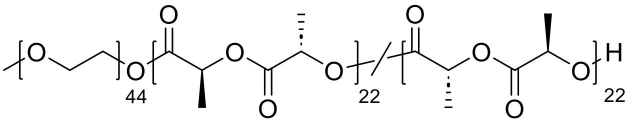

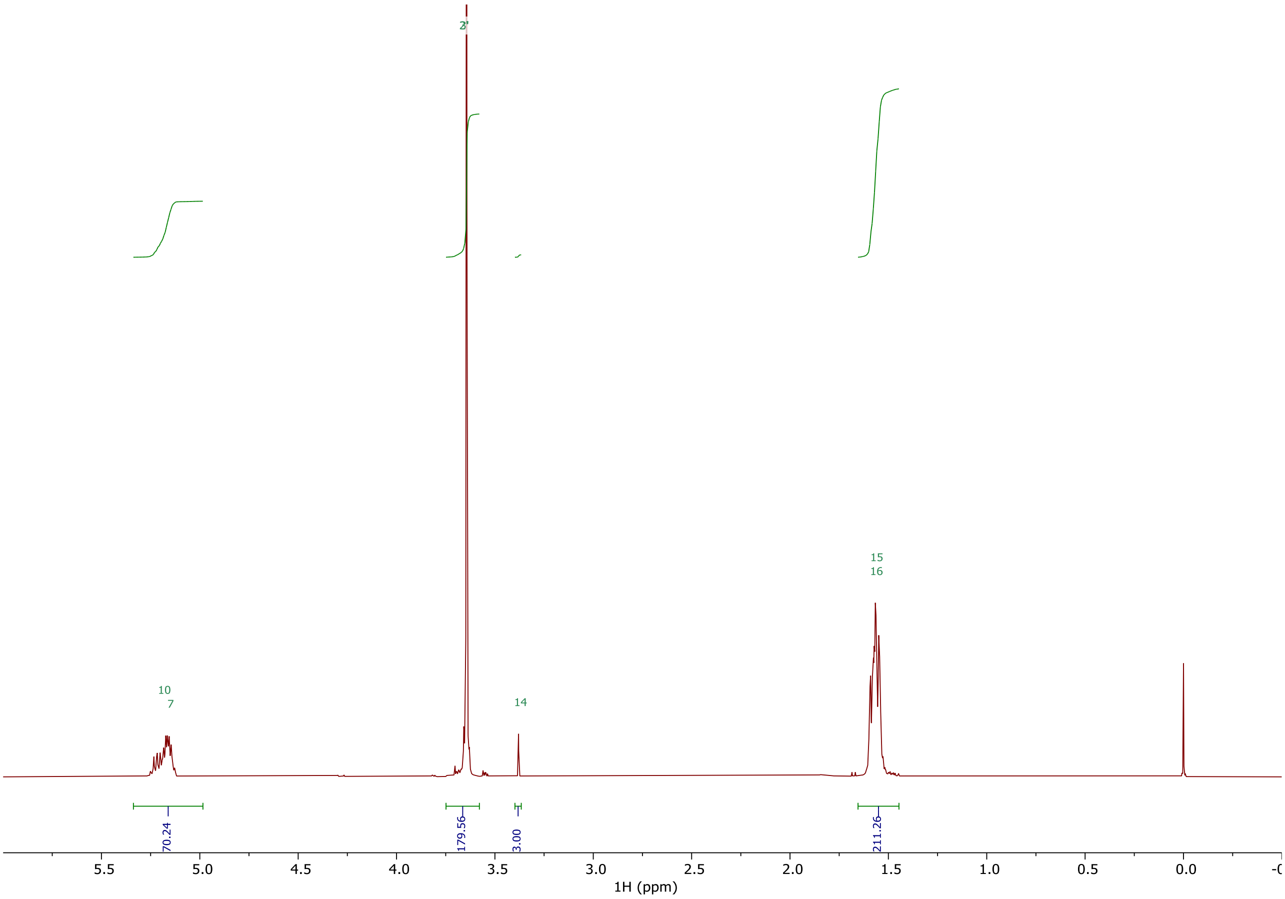

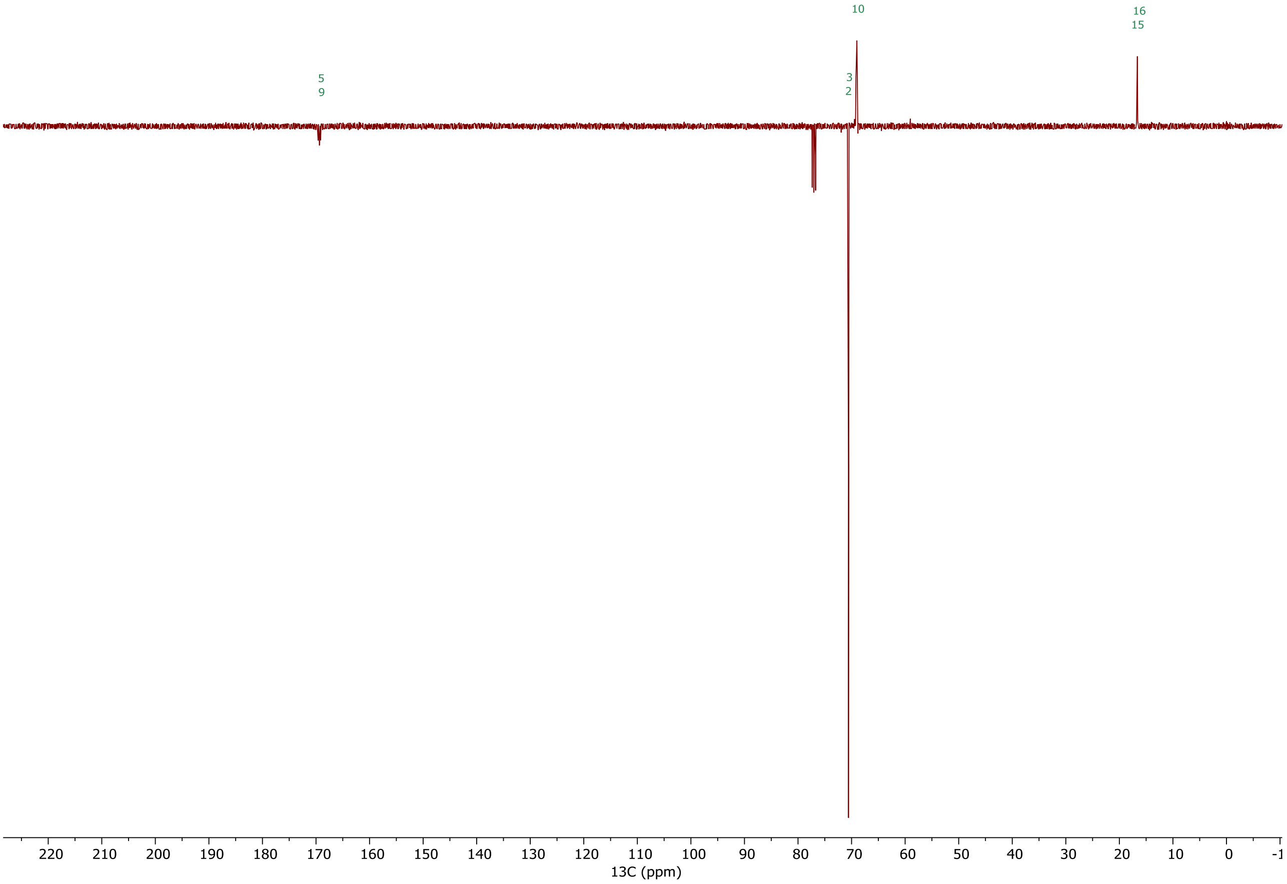
10
16
15
5
9
3
2
220 210 200 190 180 170 160 150 140 130 120 110 100 90 80 70 60 50 40 30 20 10 0
13C (ppm)

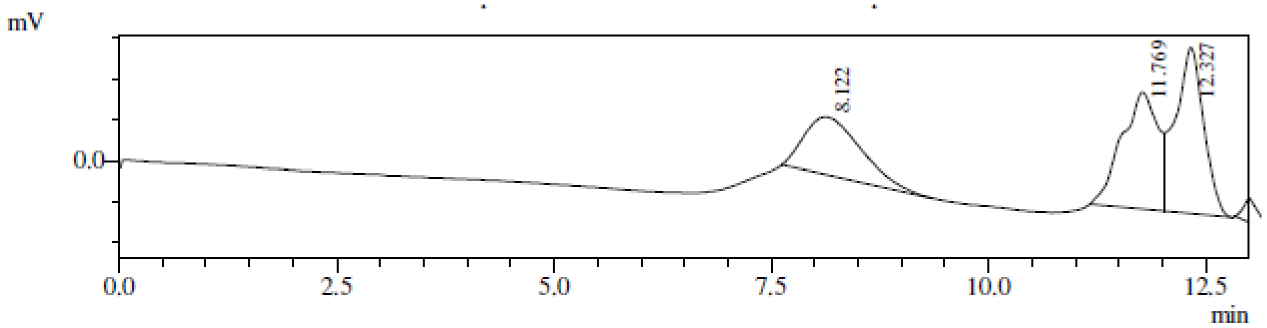
mV
0.0
8.122
11.769
12.327
0.0 2.5 5.0 7.5 10.0 12.5
min

Mw/Mn 1.09496

PEG$_{22}$-*b*-P(D+L)LA$_{90}$ **2c**

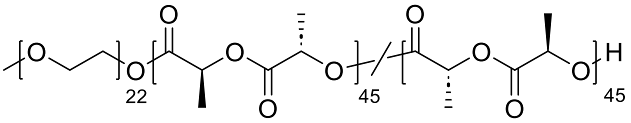

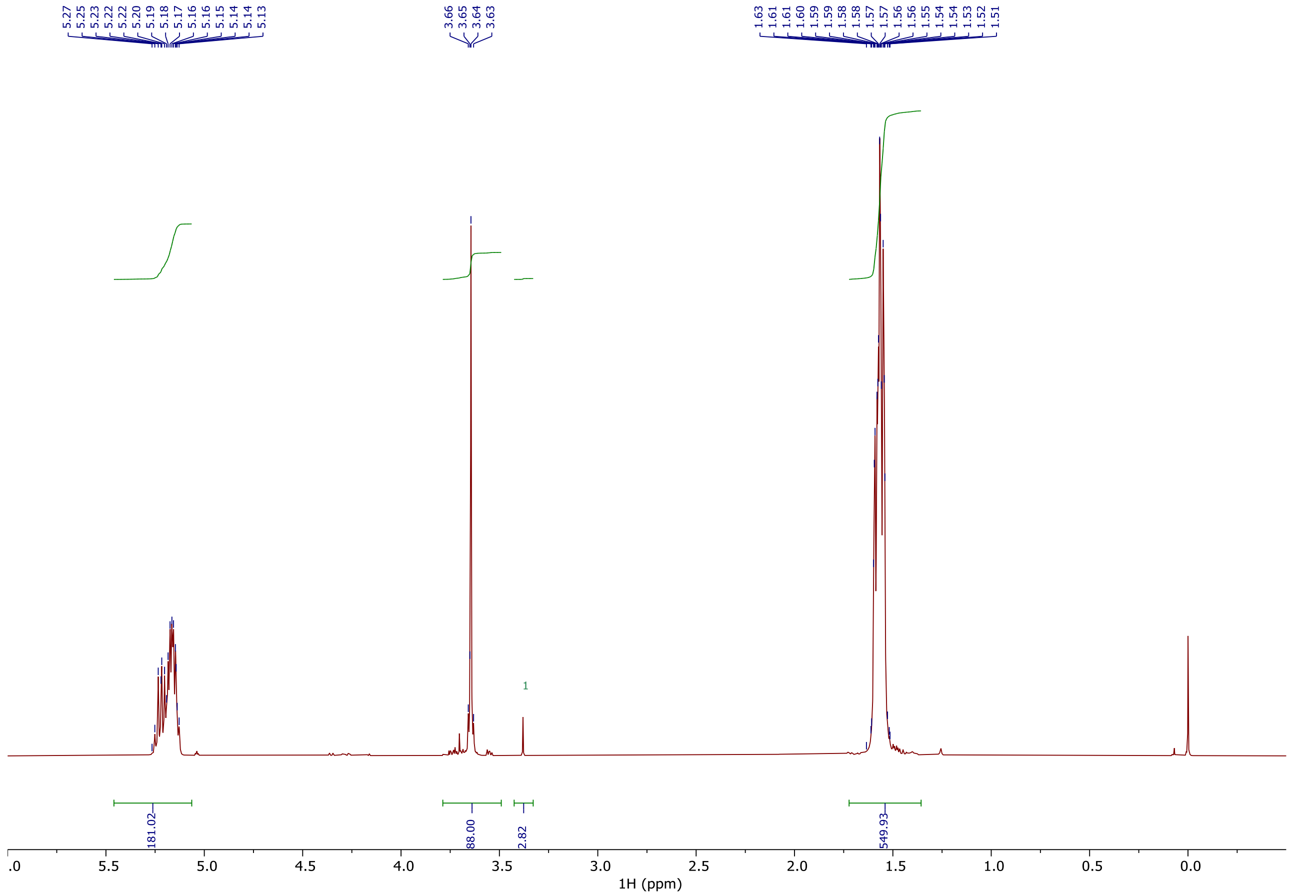

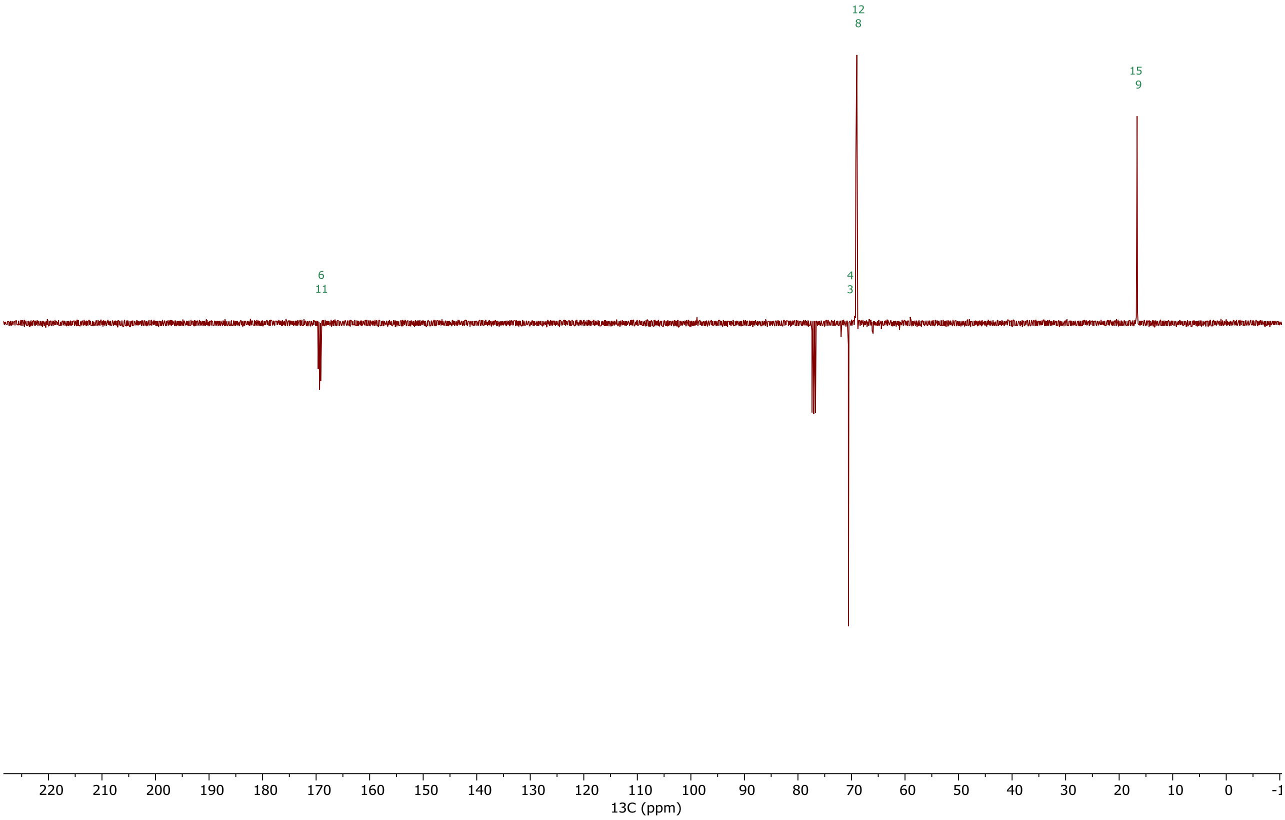

12
8
15
9
6
11
4
3
220 210 200 190 180 170 160 150 140 130 120 110 100 90 80 70 60 50 40 30 20 10 0
13C (ppm)

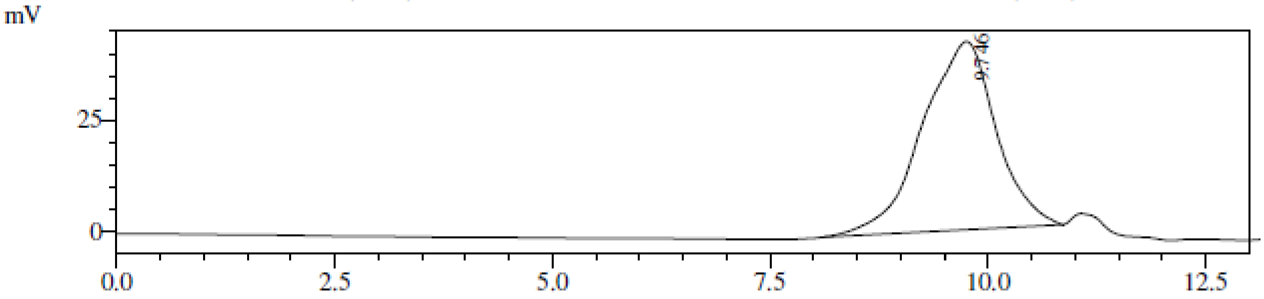

mV
25
0
9.746
0.0 2.5 5.0 7.5 10.0 12.5

Mw/Mn 1.27249

PEG$_{44}$-*b*-P(D+L)LA$_{90}$ **2d**

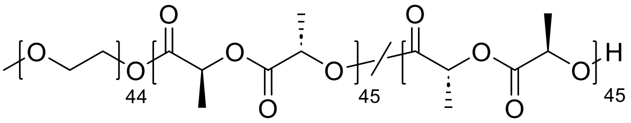

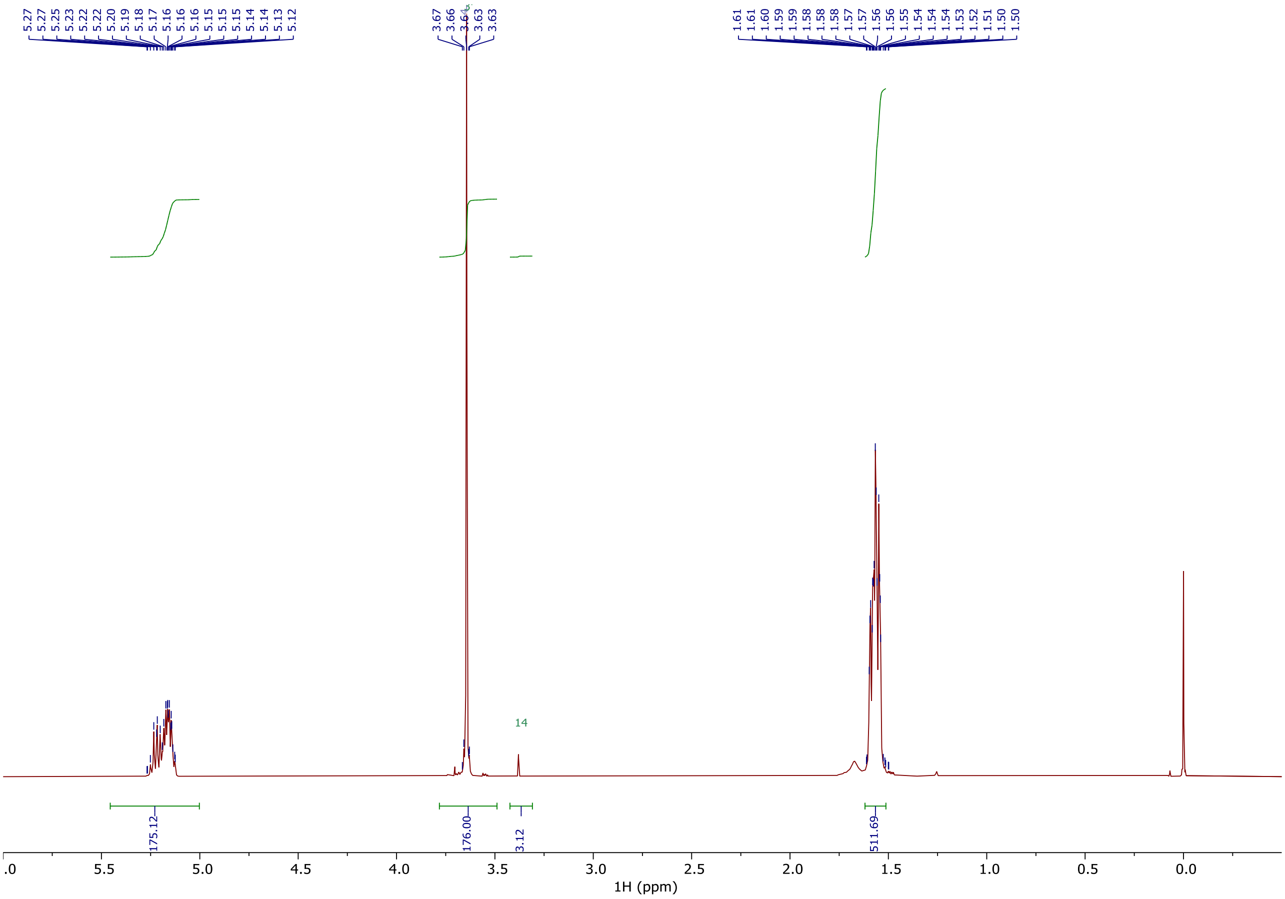

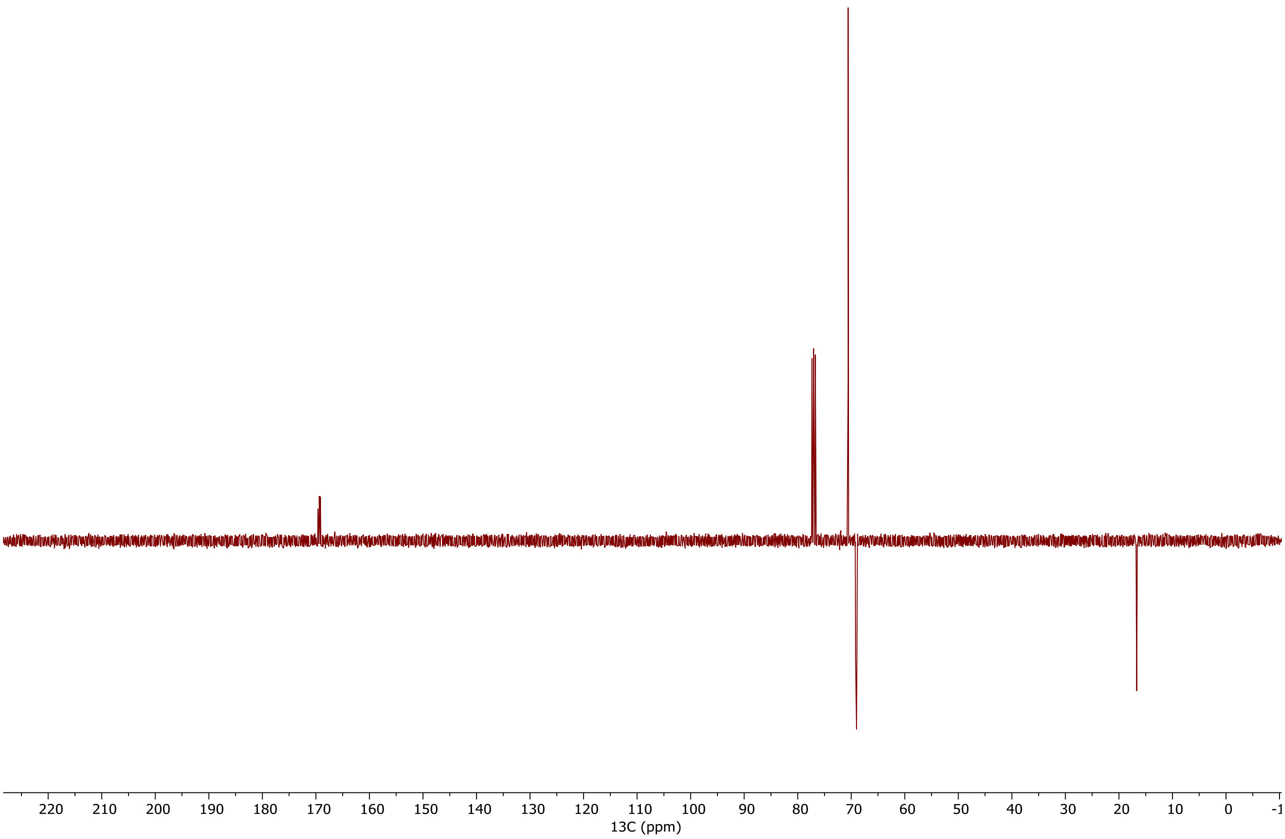
220
210
200
190
180
170
160
150
140
130
120
110
100
90
80
70
60
50
40
30
20
10
0
13C (ppm)

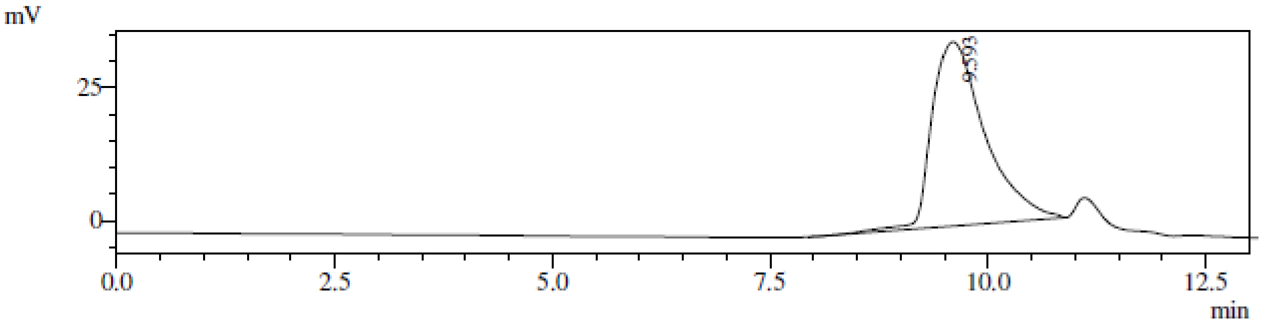
mV
25
0
9.593
0.0
2.5
5.0
7.5
10.0
12.5
min

Mw/Mn 1.16300

PEG$_{22}$-*b*-PDLA$_{22}$-*b*-PLLA$_{22}$ **3a**

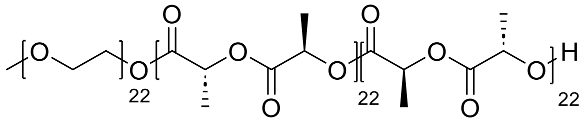

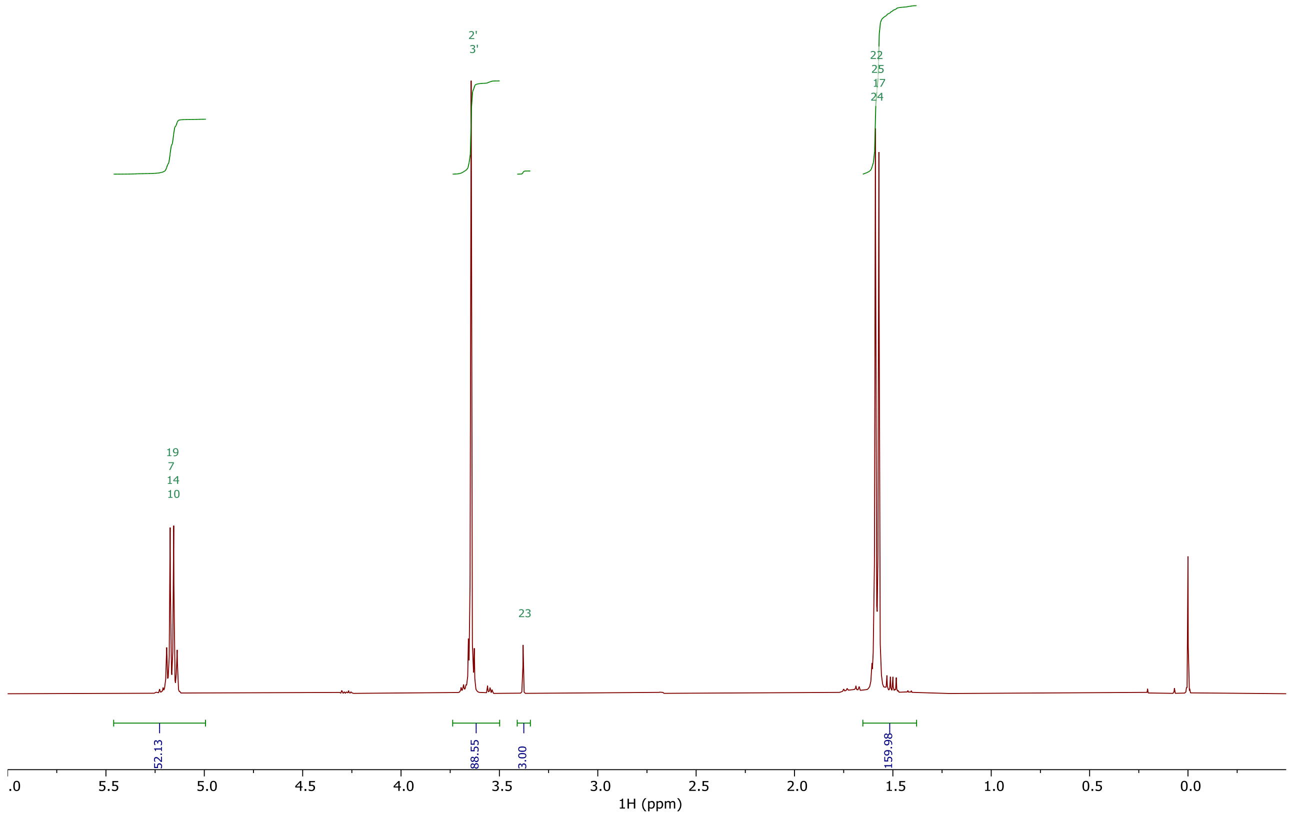

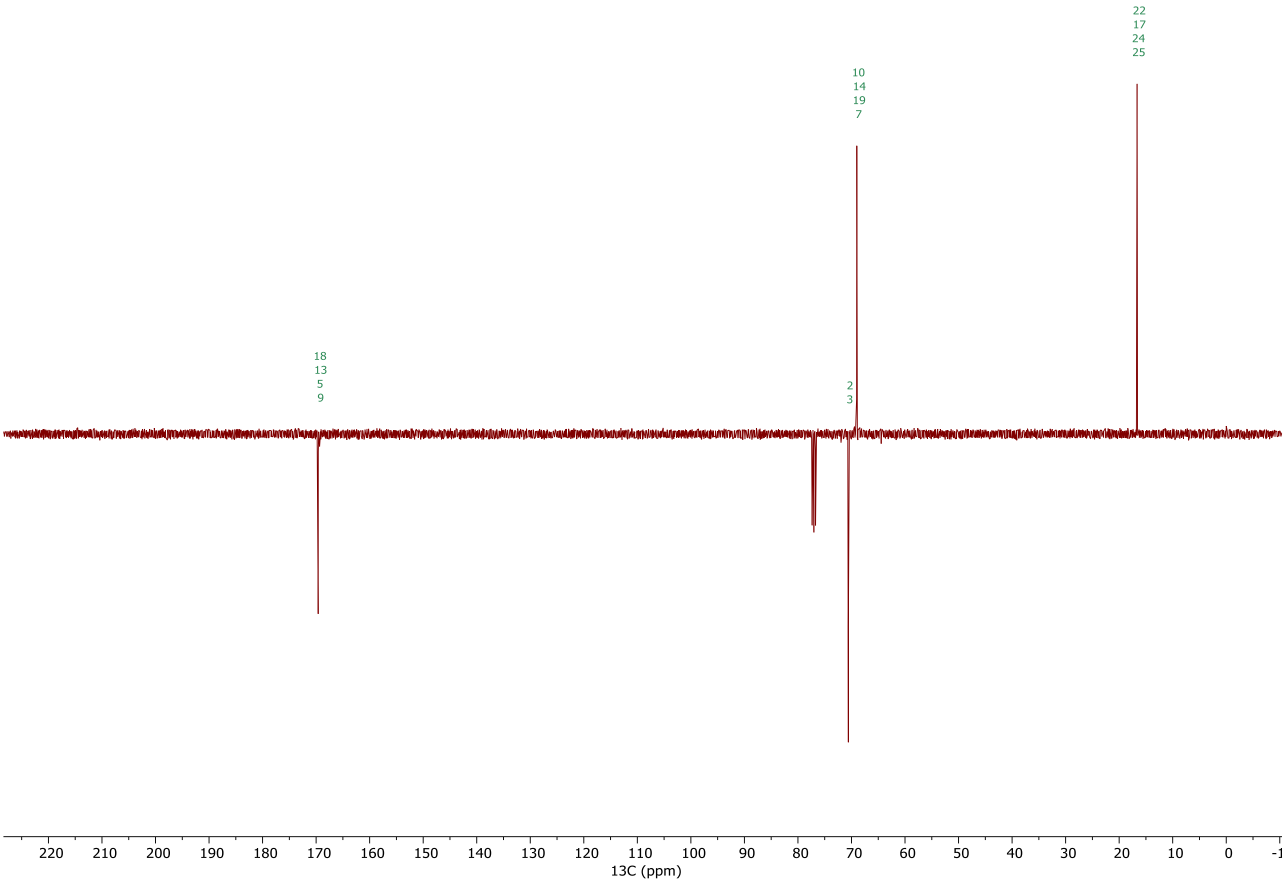
22
17
24
25
10
14
19
7
18
13
5
9
2
3
220 210 200 190 180 170 160 150 140 130 120 110 100 90 80 70 60 50 40 30 20 10 0
13C (ppm)

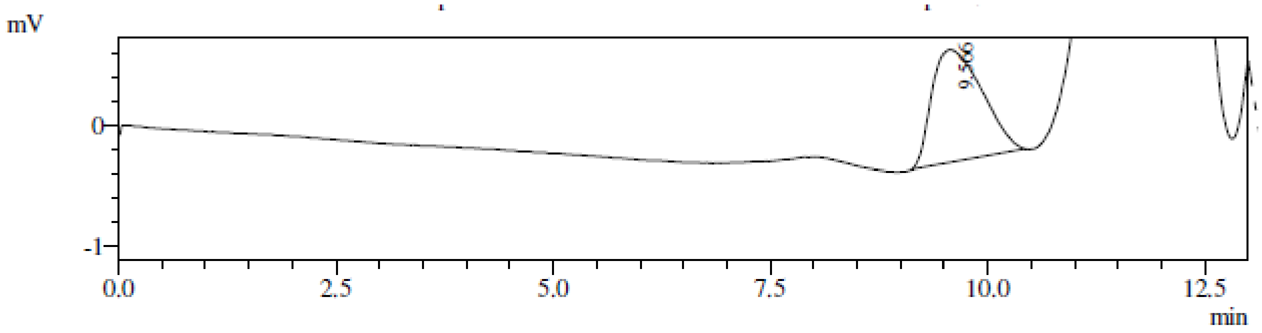
mV
0
-1
9.566
0.0 2.5 5.0 7.5 10.0 12.5
min

Mw/Mn 1.06294

PEG$_{44}$-*b*-PDLA$_{22}$-*b*-PLLA$_{22}$ **3b**

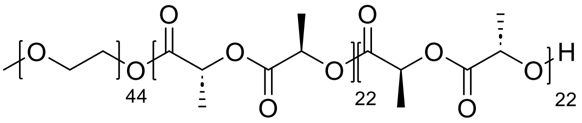

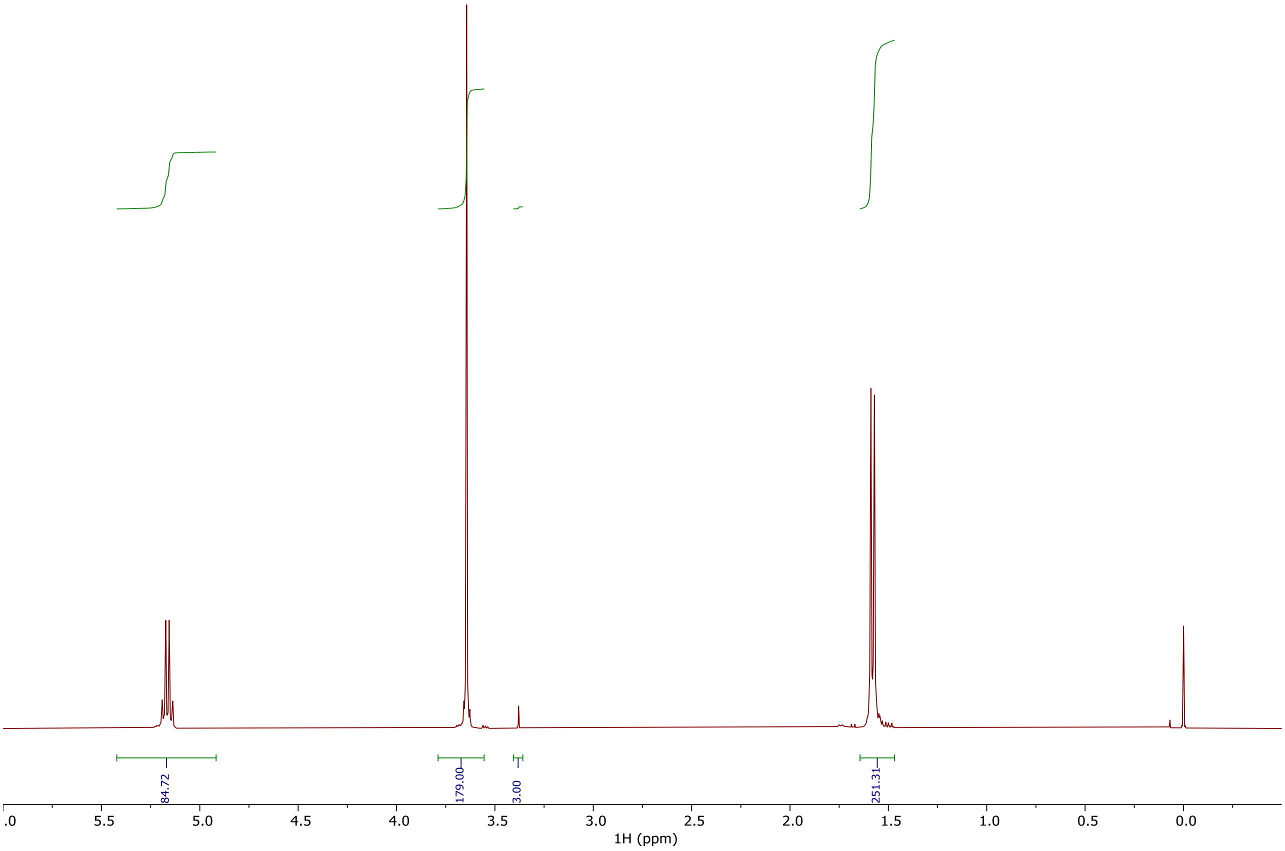

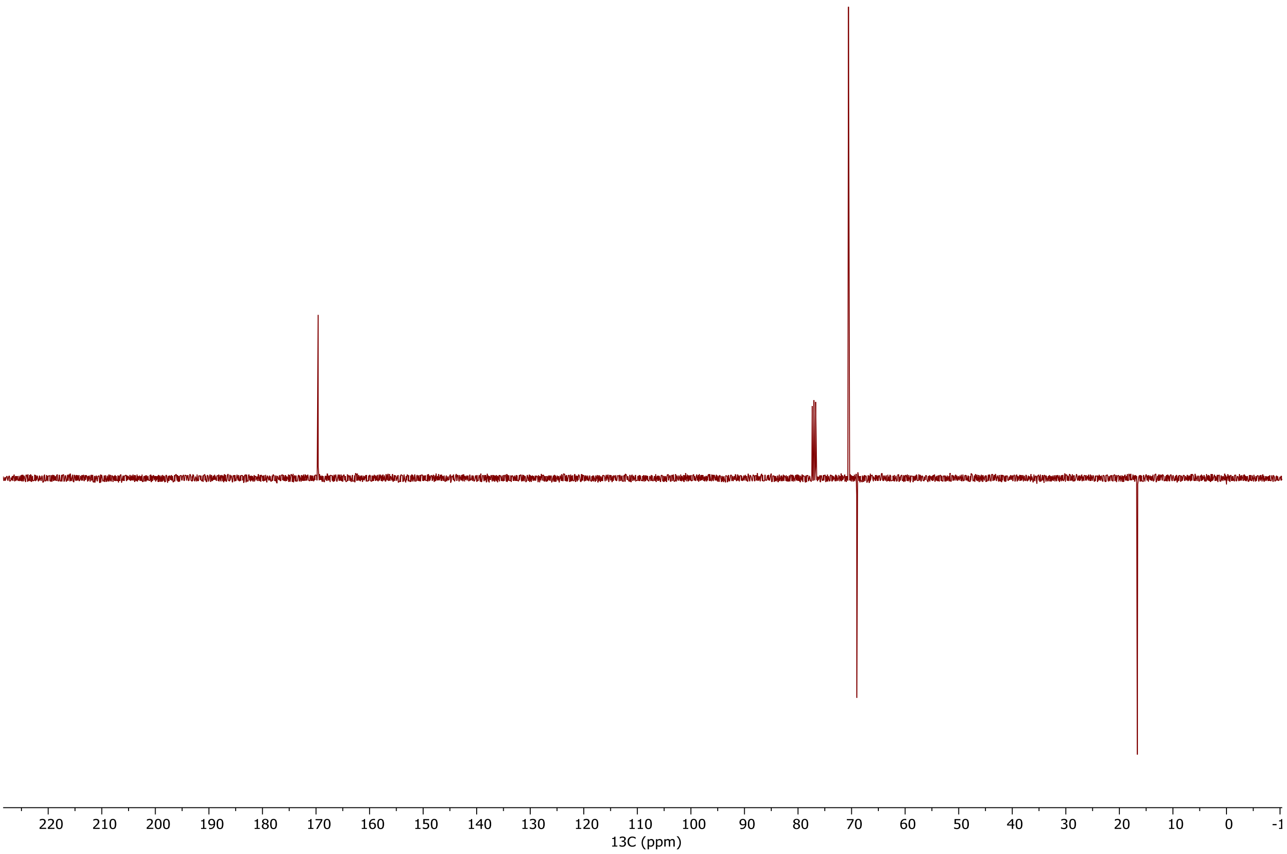
220 210 200 190 180 170 160 150 140 130 120 110 100 90 80 70 60 50 40 30 20 10 0
13C (ppm)

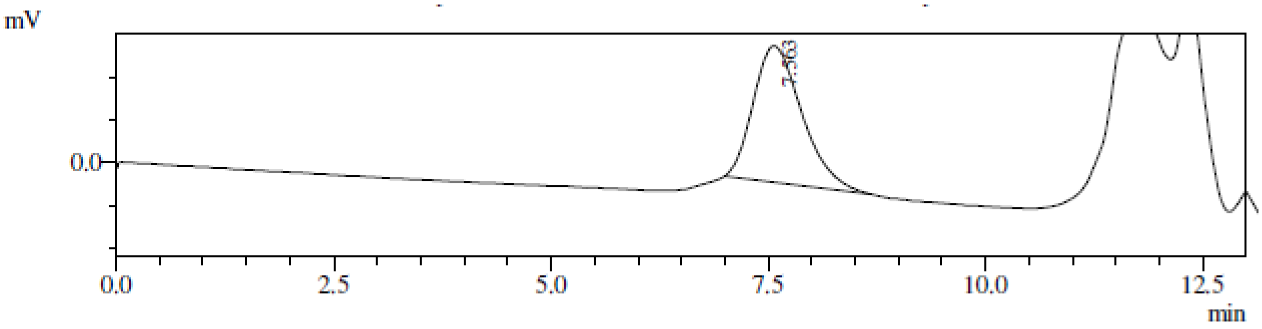
mV
0.0
0.0 2.5 5.0 7.5 10.0 12.5
min

Mw/Mn 1.07596

PEG$_{22}$-*b*-PDLA$_{45}$-*b*-PLLA$_{45}$ **3c**

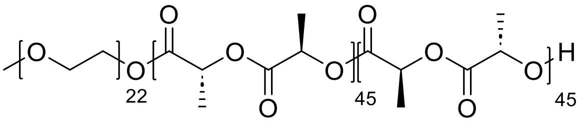

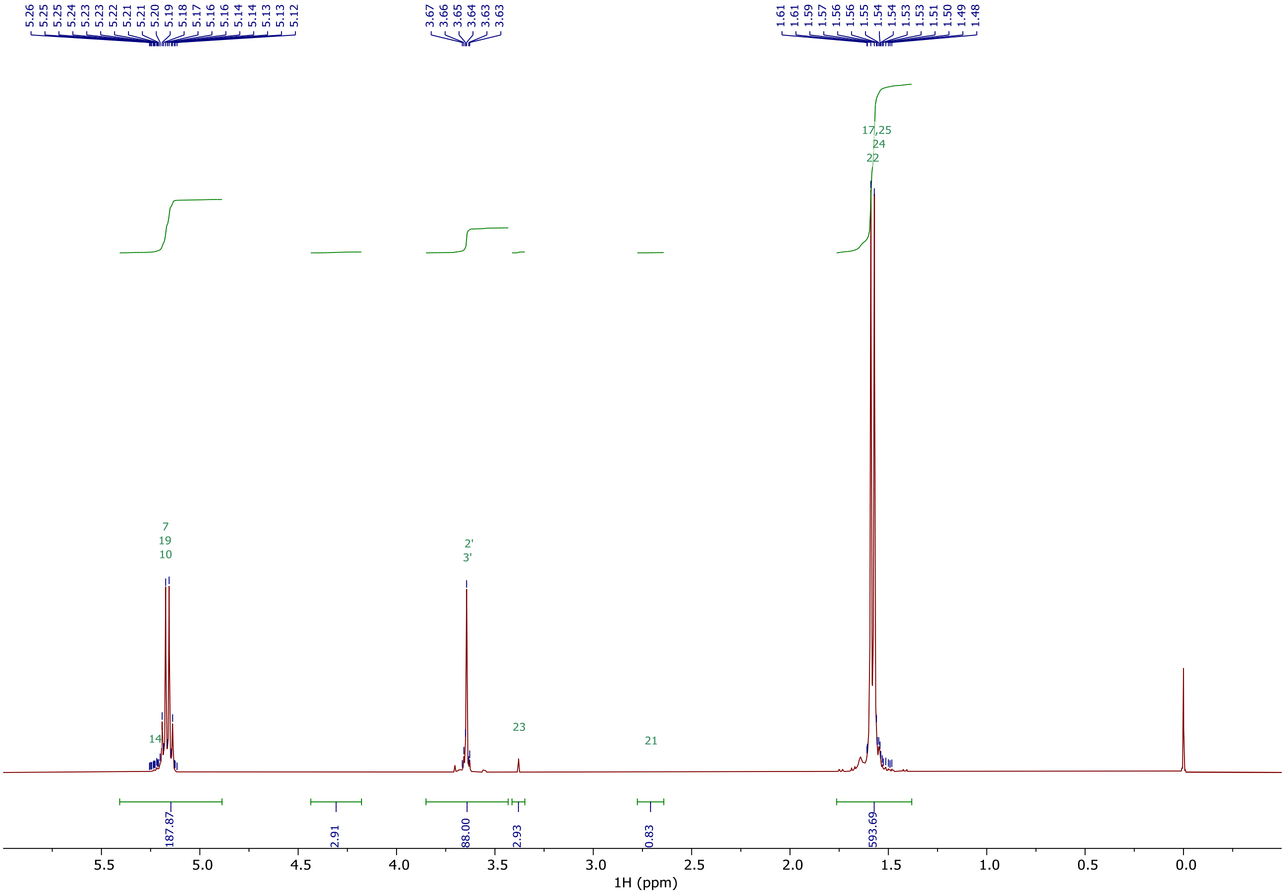

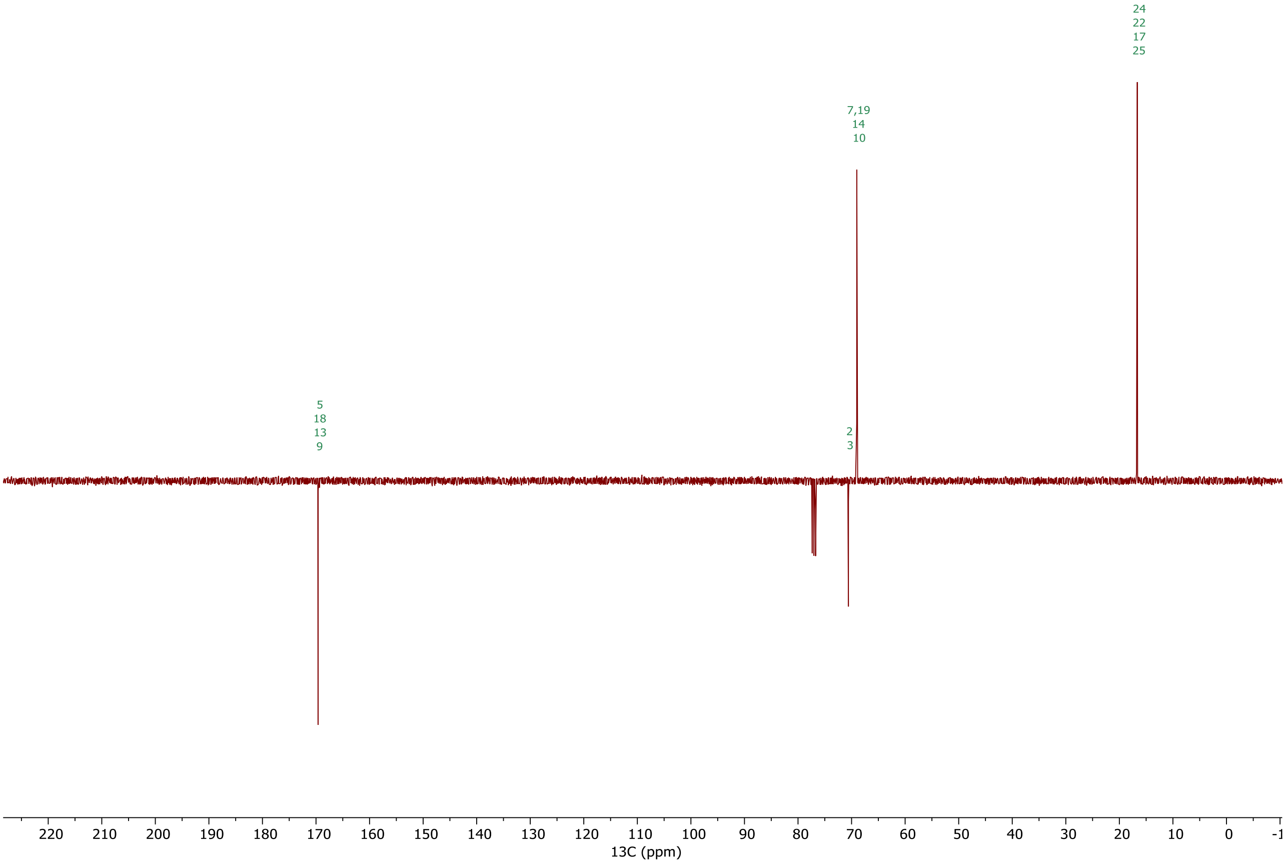
24
22
17
25
7,19
14
10
5
18
13
9
2
3
220 210 200 190 180 170 160 150 140 130 120 110 100 90 80 70 60 50 40 30 20 10 0
13C (ppm)

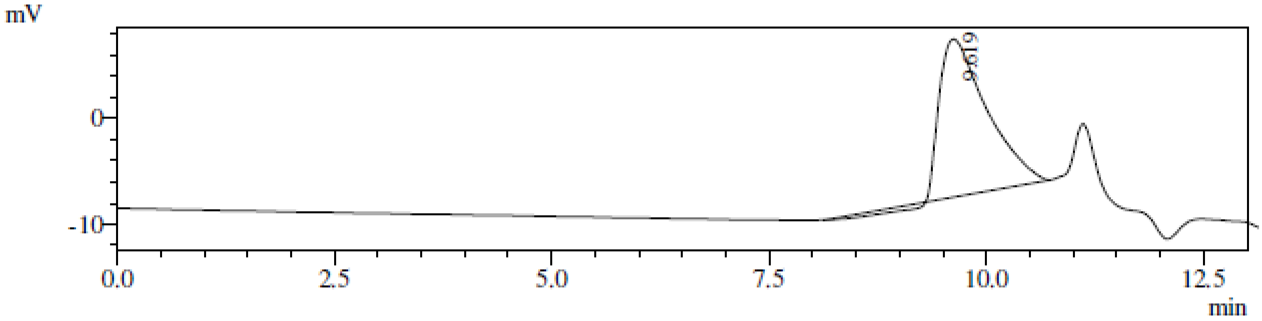
mV
0
-10
9.619
0.0 2.5 5.0 7.5 10.0 12.5
min

Mw/Mn 1.11281

PEG$_{44}$-*b*-PDLA$_{45}$-*b*-PLLA$_{45}$ **3d**

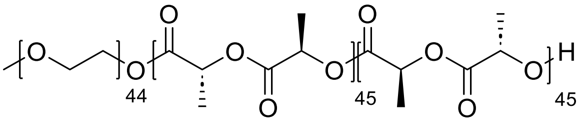

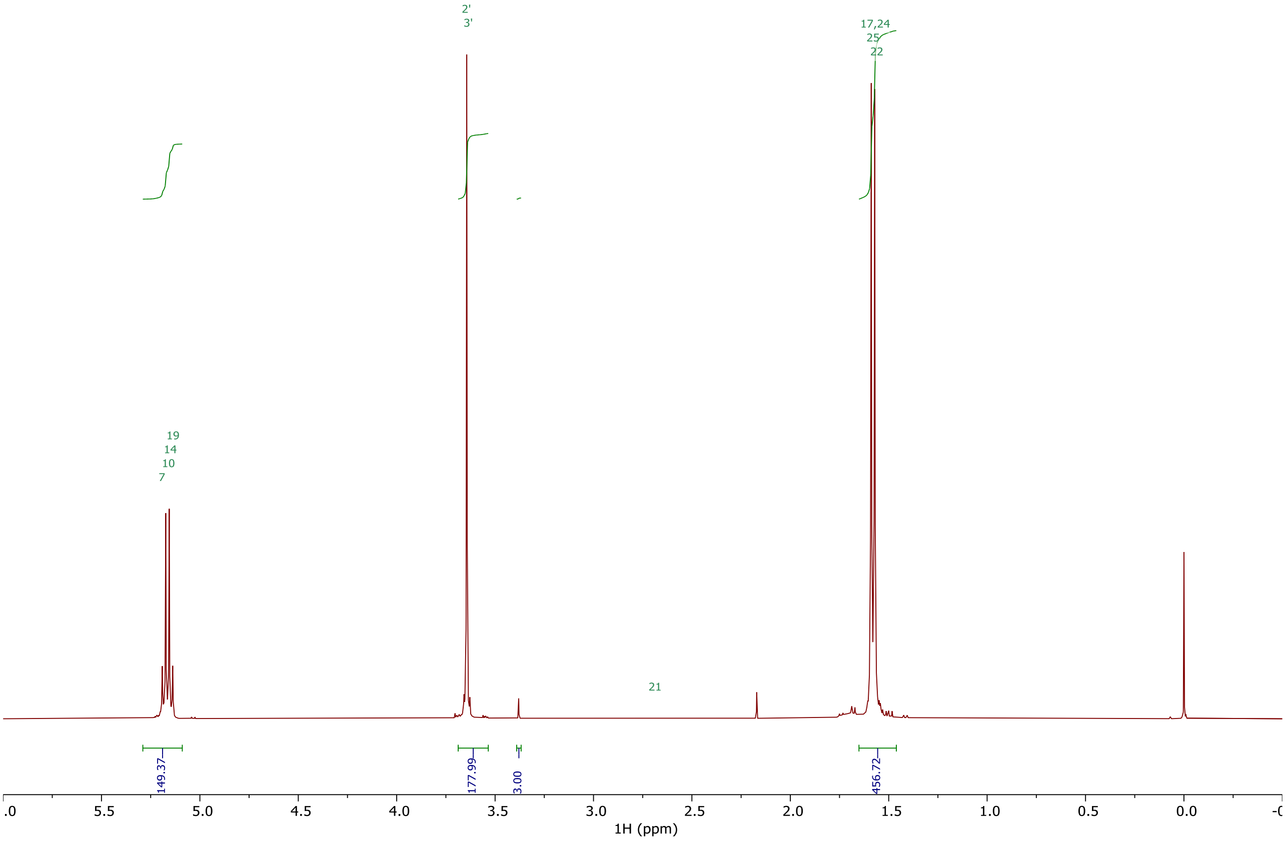

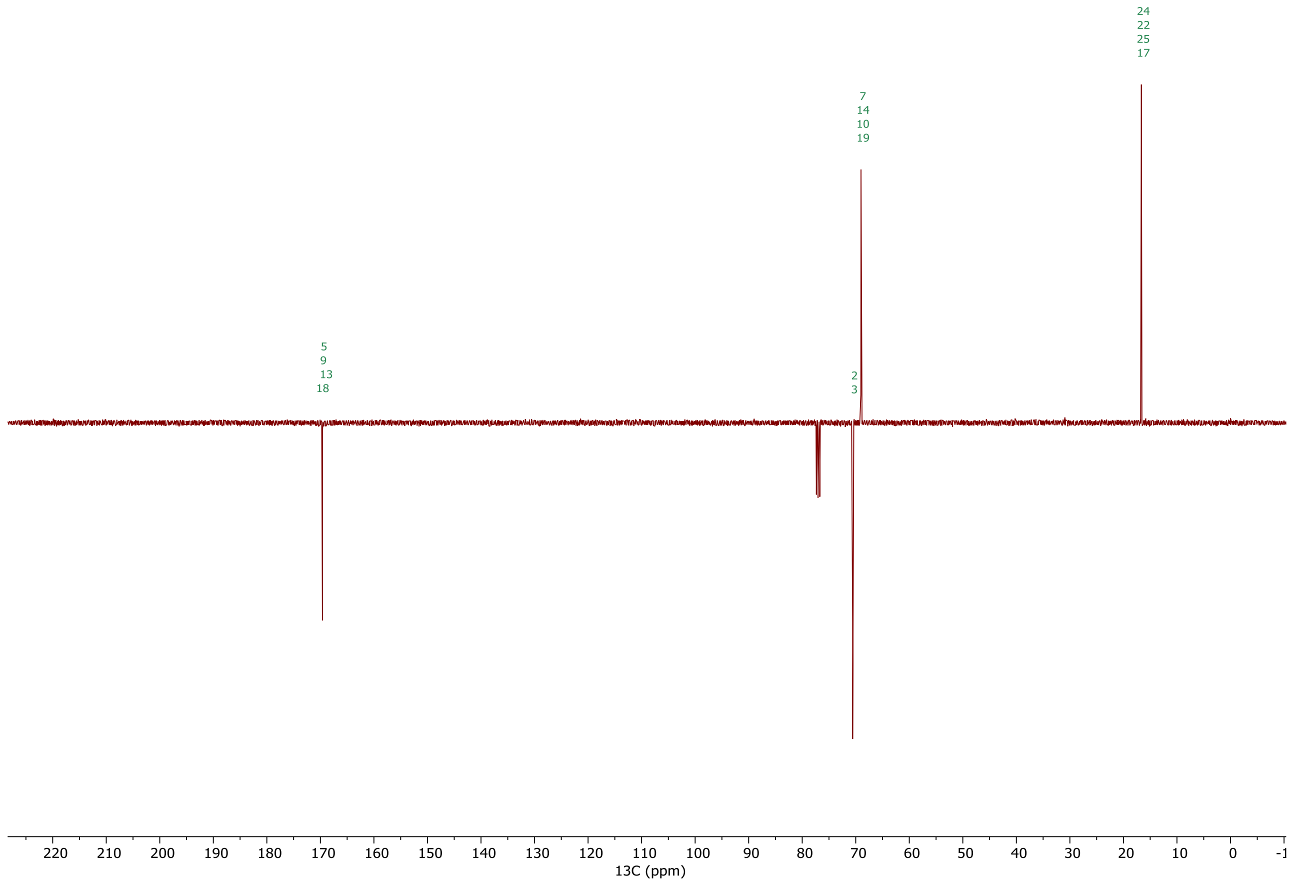
24
22
25
17
7
14
10
19
5
9
13
18
2
3
220 210 200 190 180 170 160 150 140 130 120 110 100 90 80 70 60 50 40 30 20 10 0
13C (ppm)

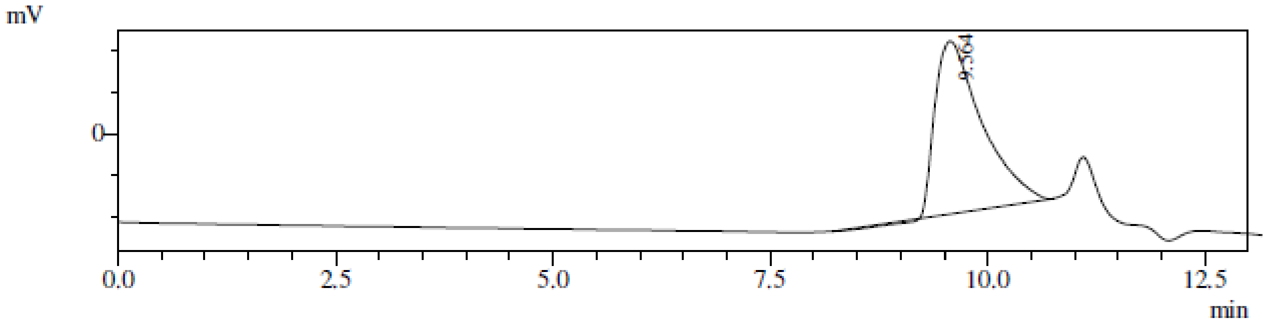
mV
0
9.564
0.0
2.5
5.0
7.5
10.0
12.5
min

Mw/Mn 1.11633

PEG$_{22}$-*b*-PLLA$_{22}$-*b*-PDLA$_{22}$ **4a**

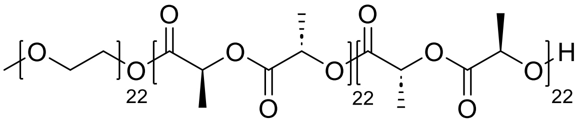

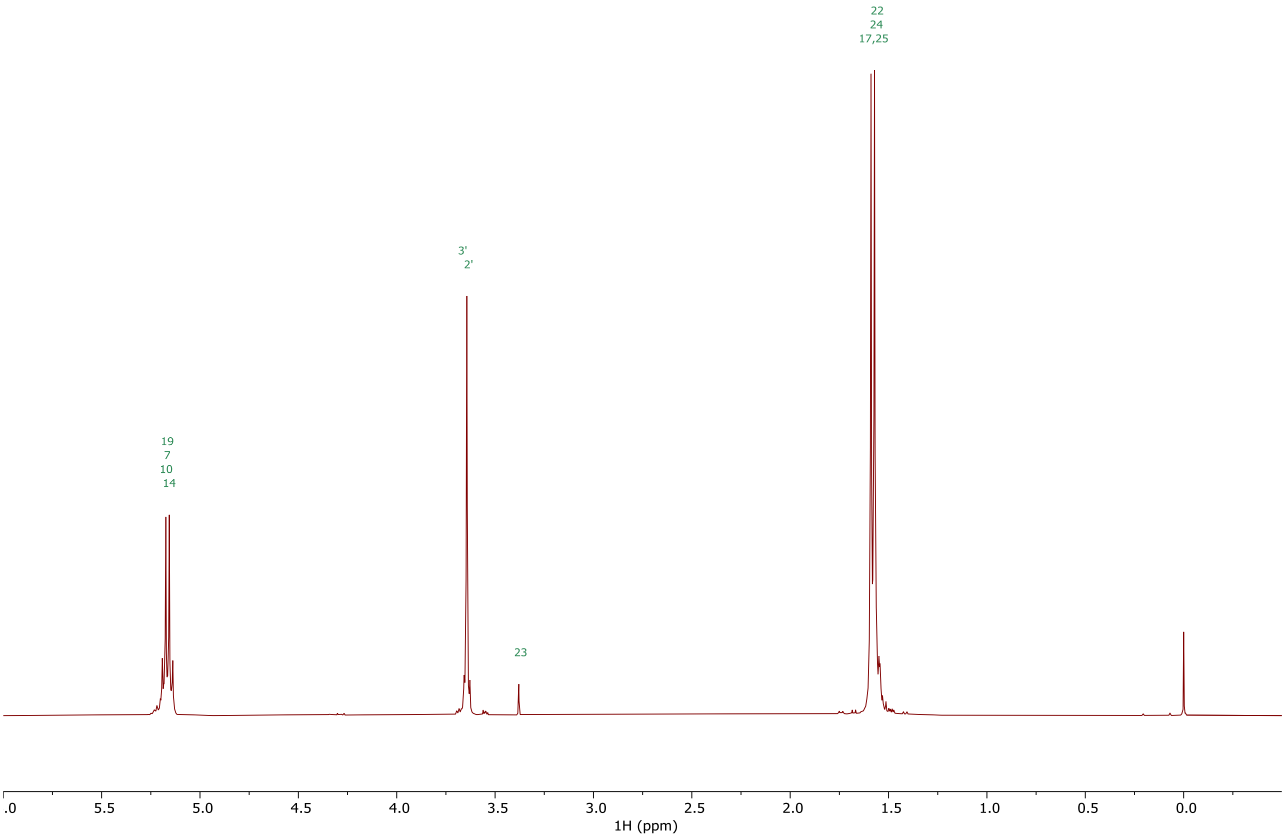

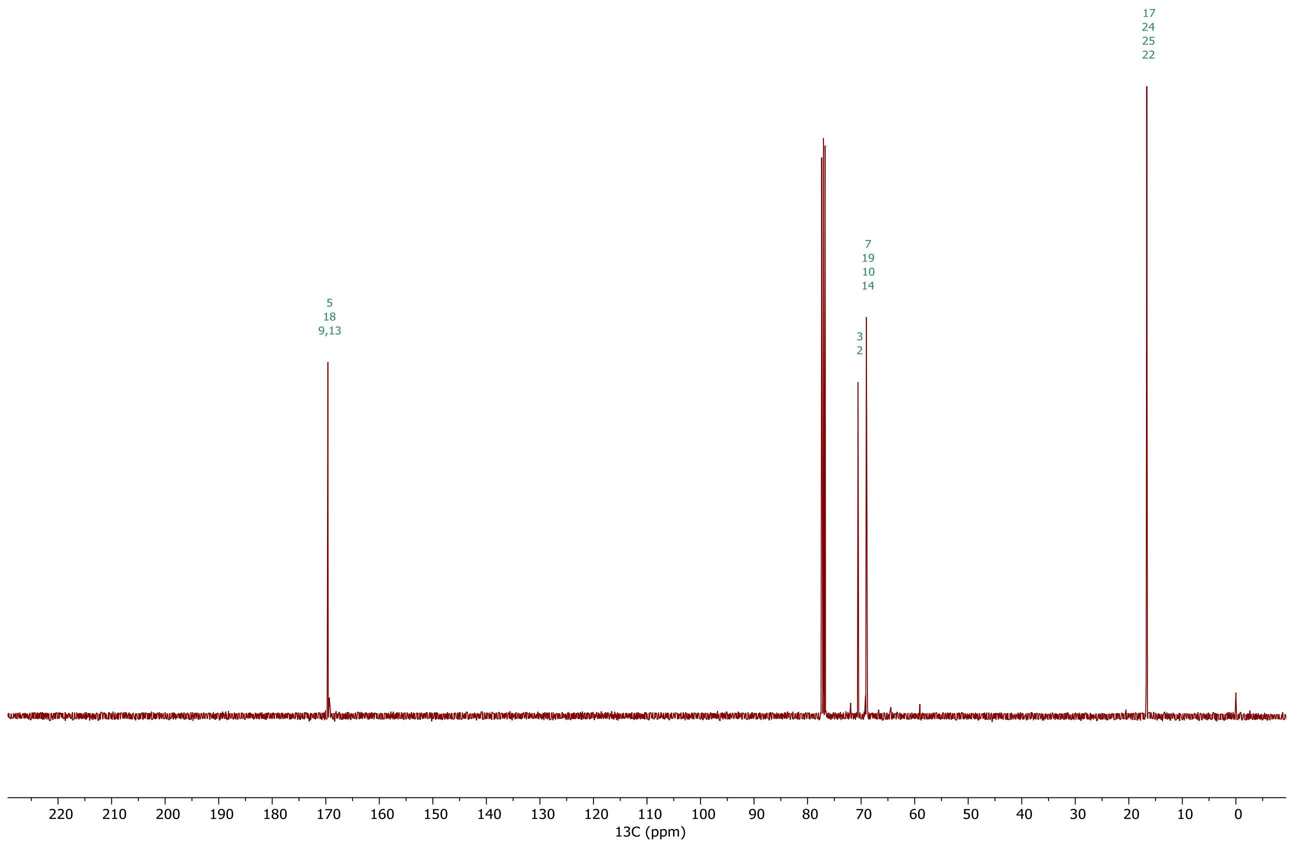

17
24
25
22
7
19
10
14
3
2
5
18
9,13
220 210 200 190 180 170 160 150 140 130 120 110 100 90 80 70 60 50 40 30 20 10 0
13C (ppm)

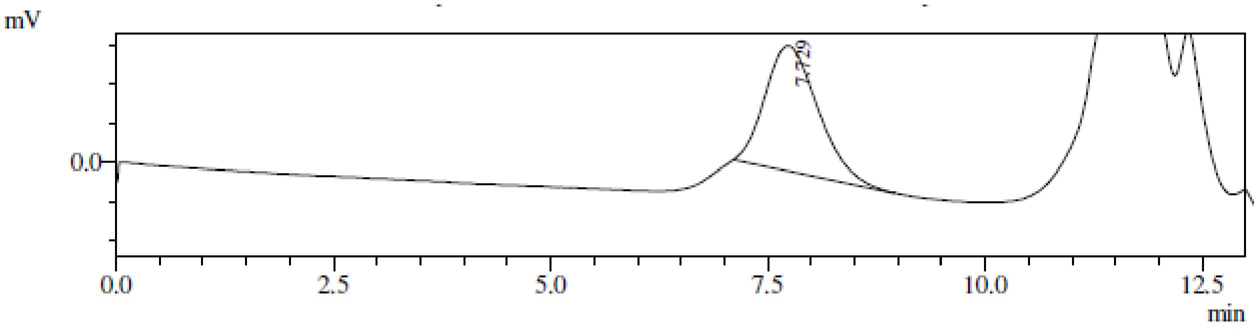

mV
0.0
7.729
0.0 2.5 5.0 7.5 10.0 12.5
min

Mw/Mn 1.08638

PEG$_{44}$-*b*-PLLA$_{22}$-*b*-PDLA$_{22}$ **4b**

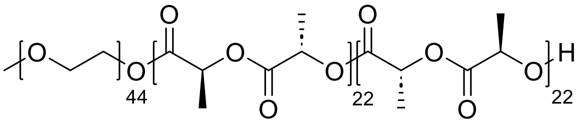

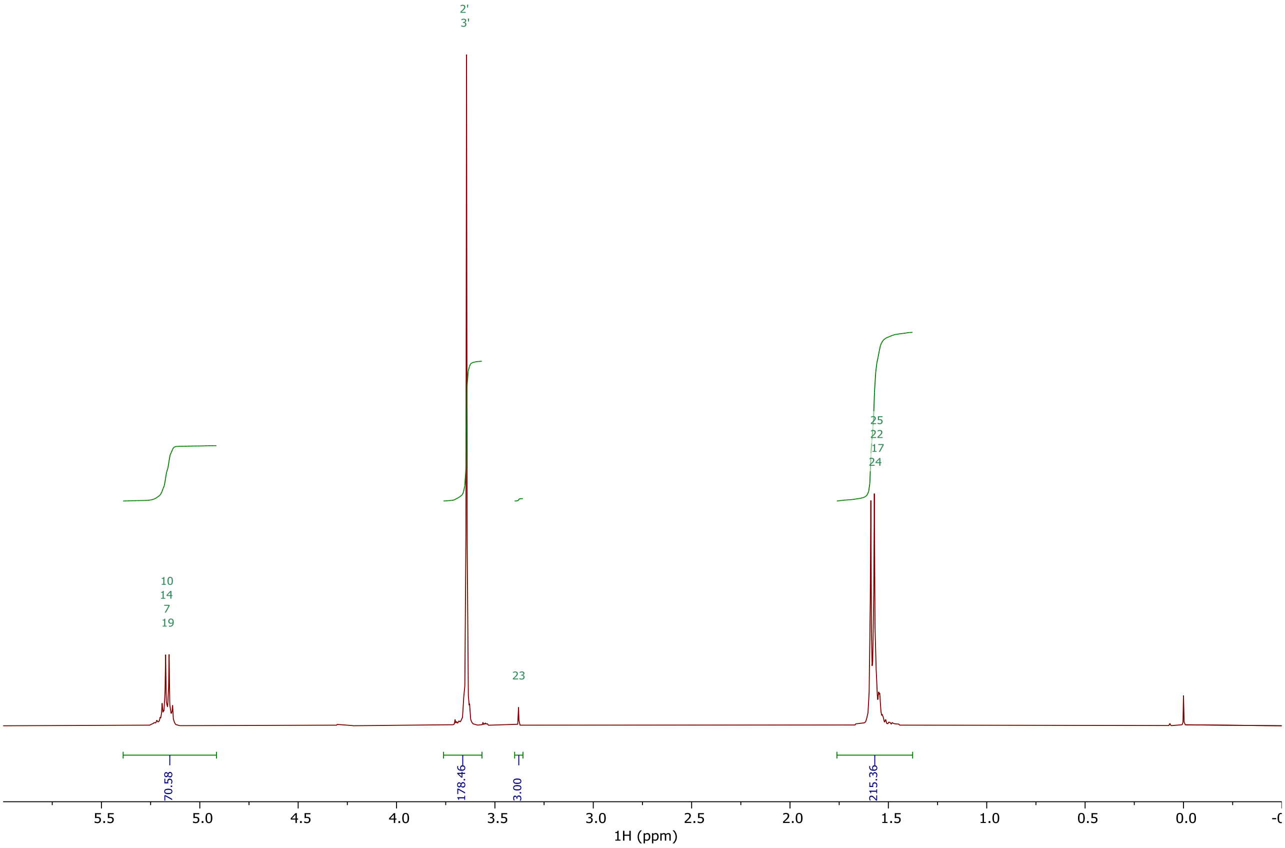

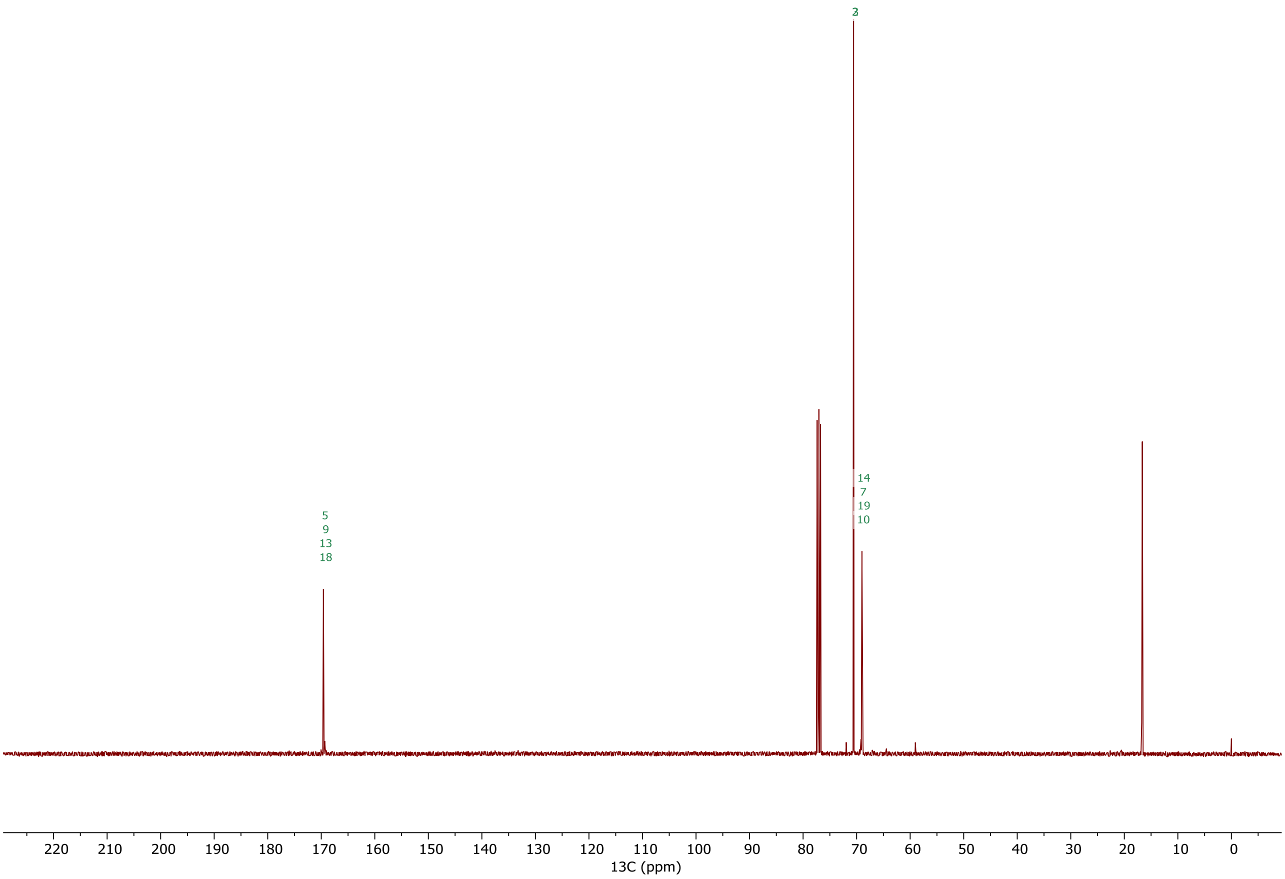
5
9
13
18
14
7
19
10
220 210 200 190 180 170 160 150 140 130 120 110 100 90 80 70 60 50 40 30 20 10 0
13C (ppm)

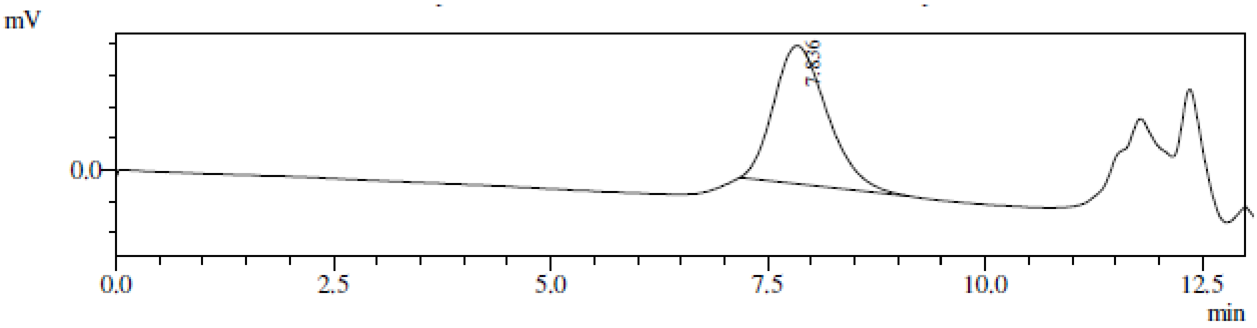
mV
0.0
0.0 2.5 5.0 7.5 10.0 12.5
min

Mw/Mn 1.09245

PEG$_{22}$-*b*-PLLA$_{45}$-*b*-PDLA$_{45}$ **4c**

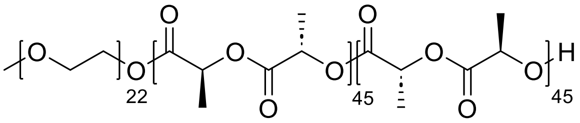

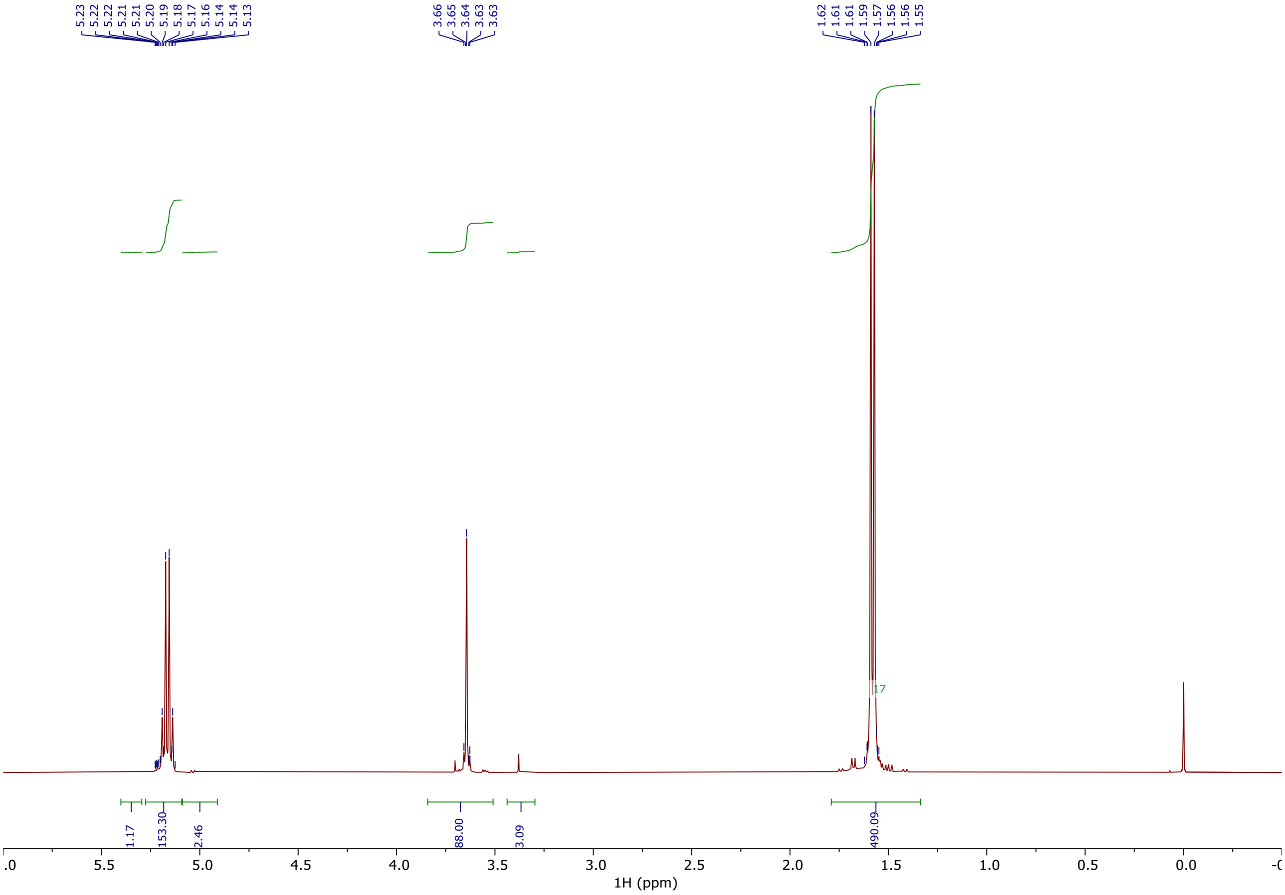

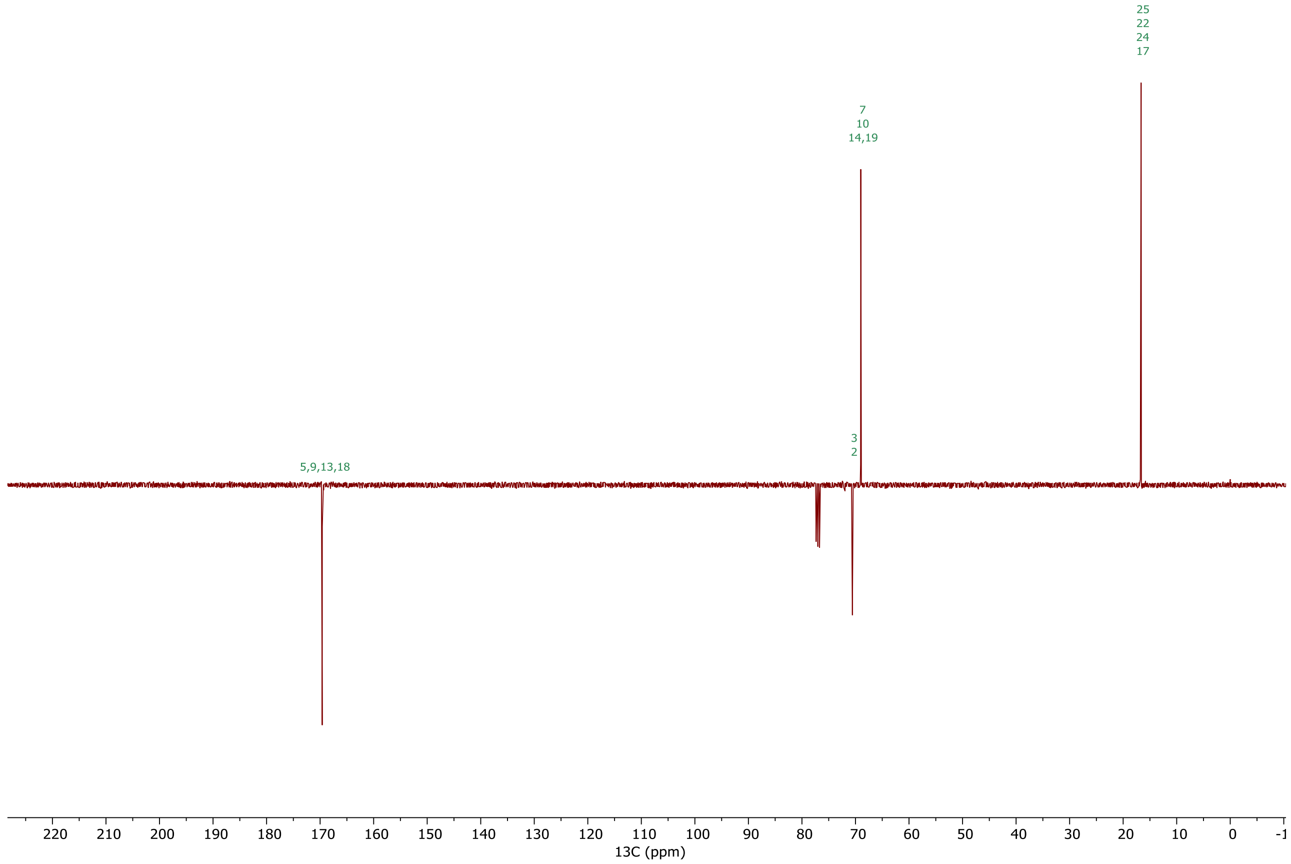
25
22
24
17
7
10
14,19
3
2
5,9,13,18
220 210 200 190 180 170 160 150 140 130 120 110 100 90 80 70 60 50 40 30 20 10 0
13C (ppm)

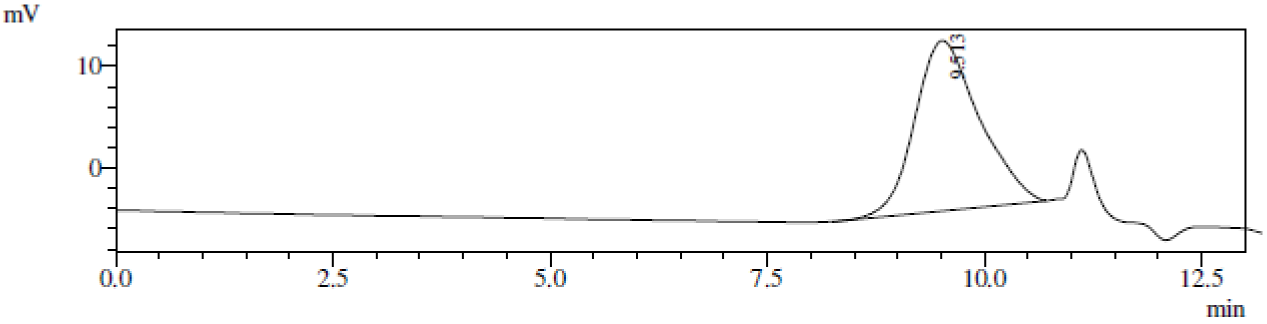
mV
10
0
9.513
0.0 2.5 5.0 7.5 10.0 12.5
min

Mw/Mn 1.19484

PEG$_{44}$-*b*-PLLA$_{45}$-*b*-PDLA$_{45}$ **4d**

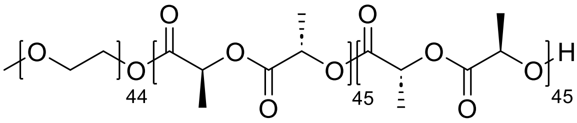

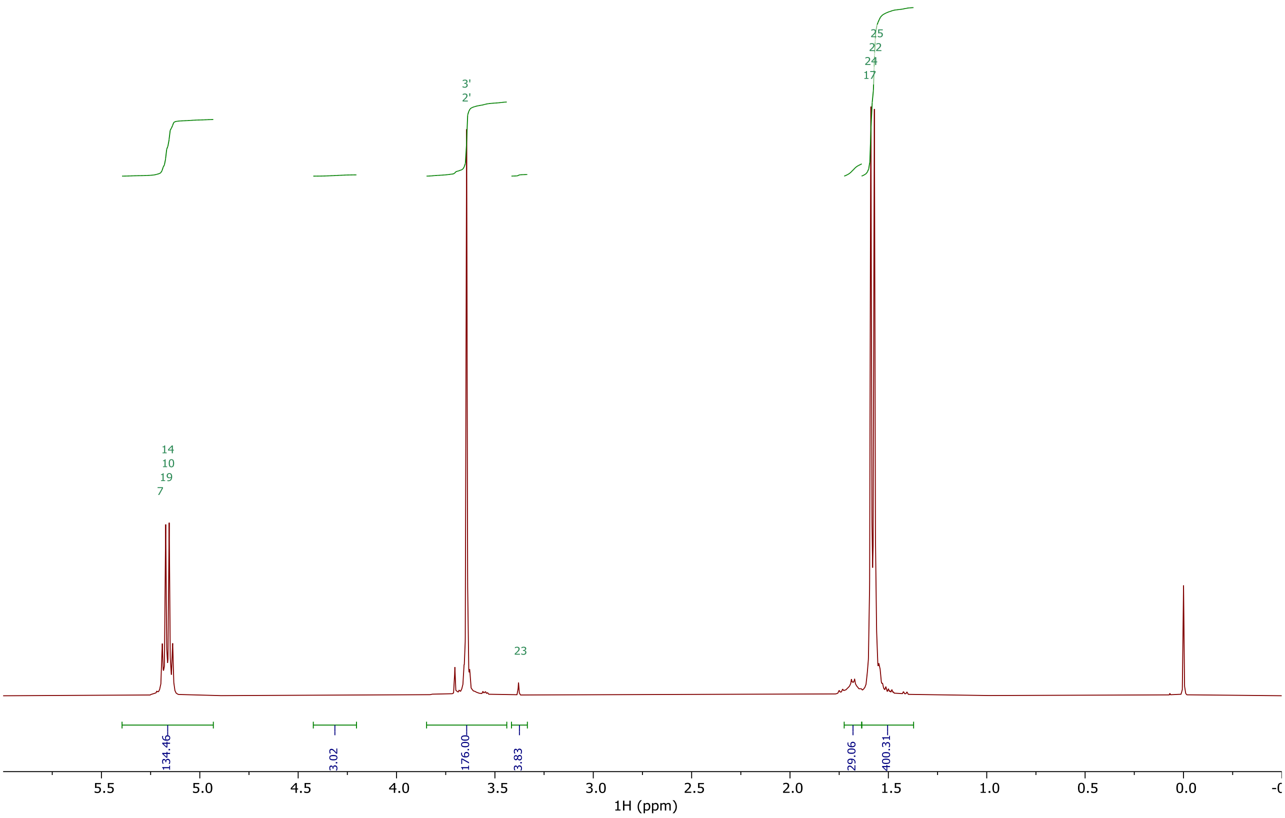

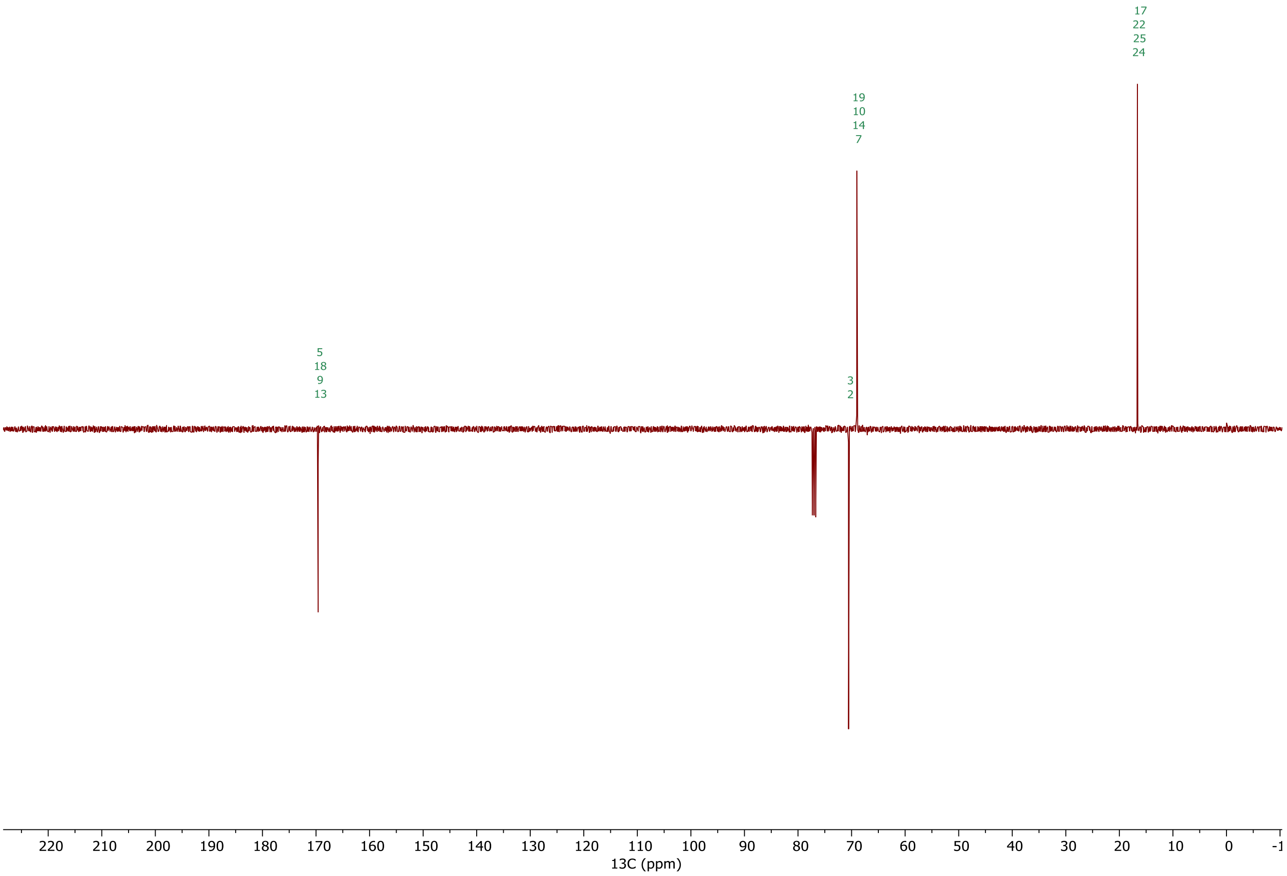
17
22
25
24
19
10
14
7
5
18
9
13
3
2
220 210 200 190 180 170 160 150 140 130 120 110 100 90 80 70 60 50 40 30 20 10 0 -1
13C (ppm)

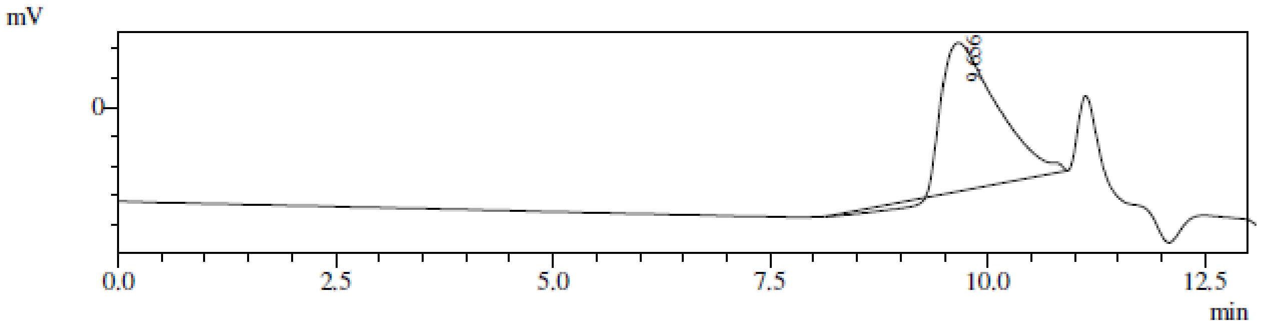
mV
0
9.656
0.0 2.5 5.0 7.5 10.0 12.5
min

Mw/Mn 1.15551

PEG$_{22}$-*b*-PLLA$_{45}$ **5a**

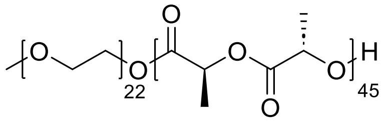

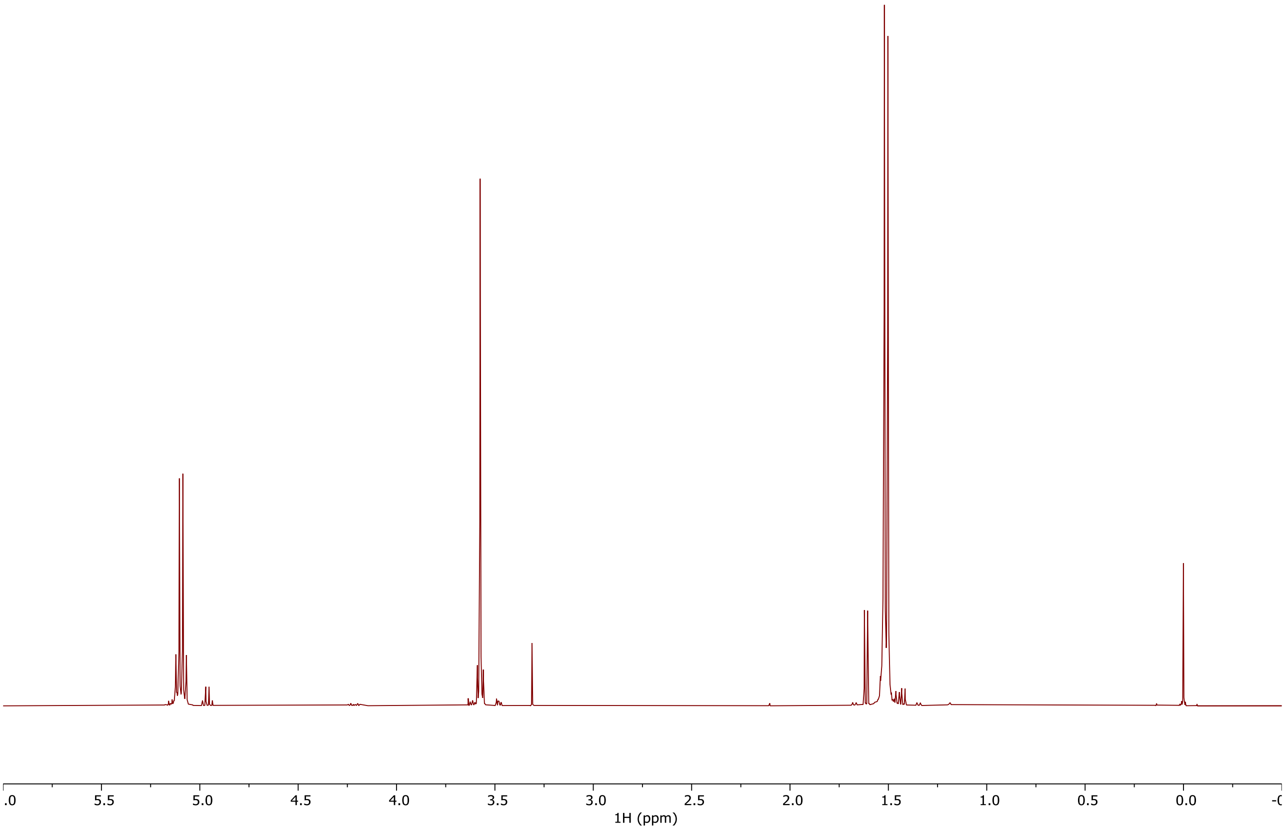

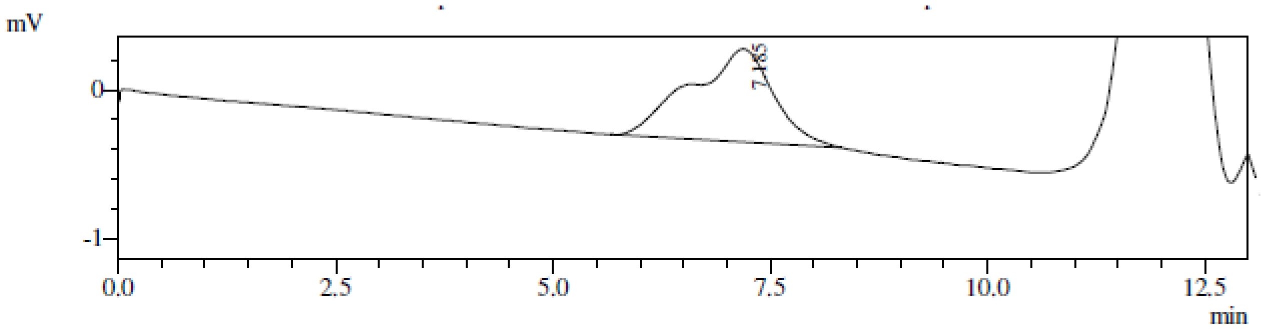

Mw/Mn 1.24405

PEG$_{22}$-*b*-PLLA$_{90}$ **5b**

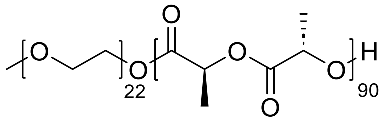

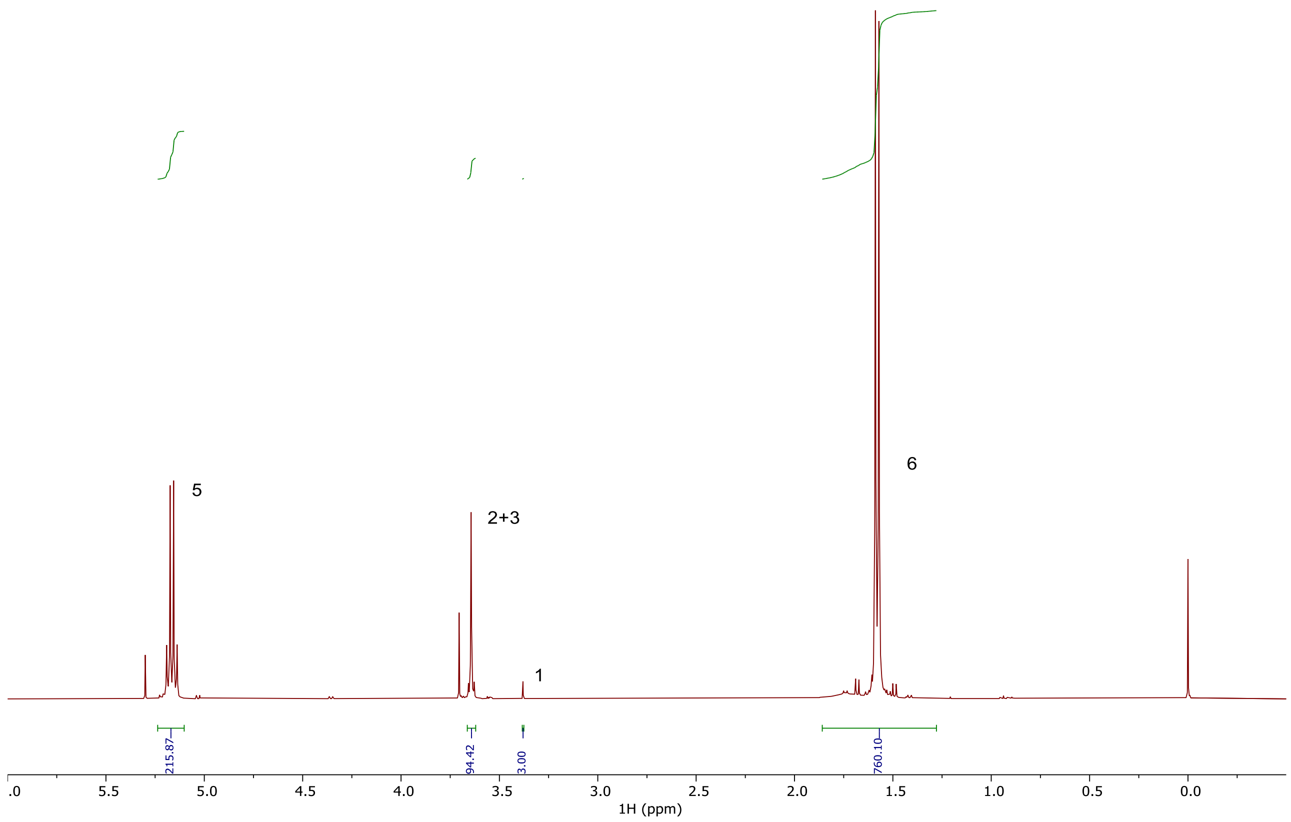

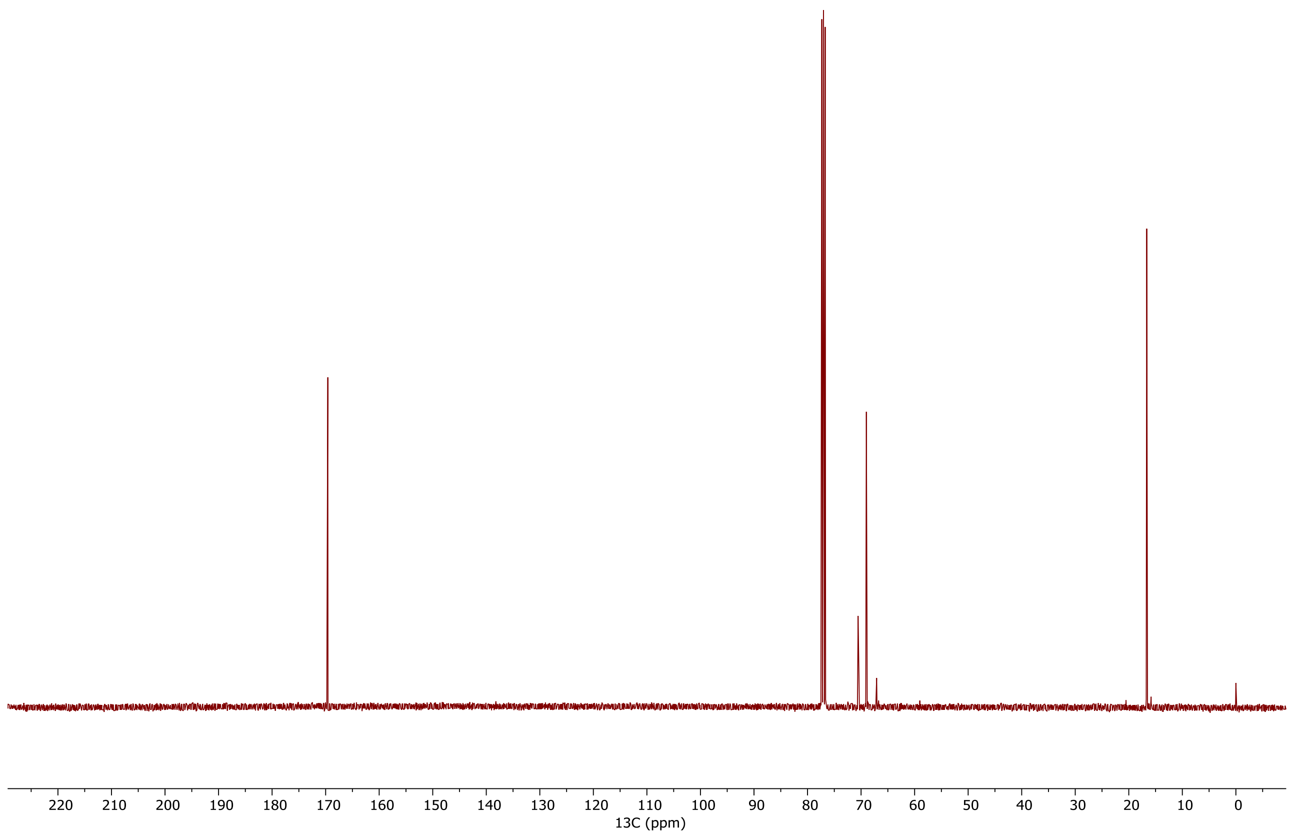

220
210
200
190
180
170
160
150
140
130
120
110
100
90
80
70
60
50
40
30
20
10
0
13C (ppm)

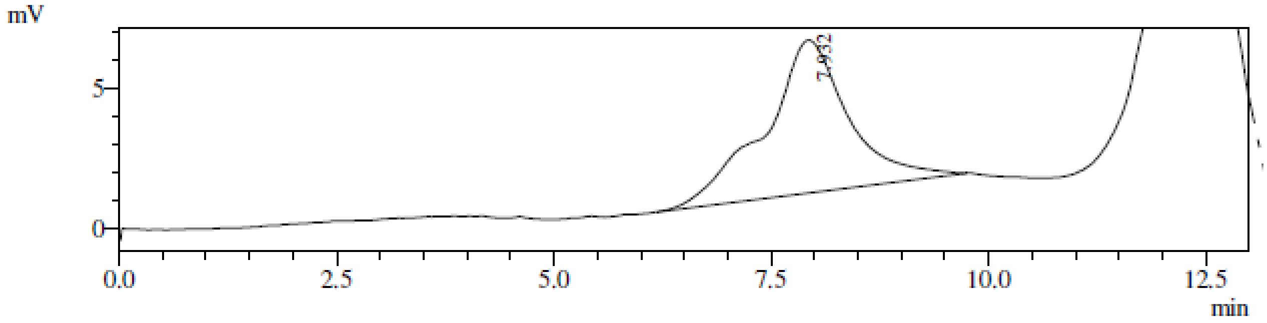

mV
5
0
7.932
0.0
2.5
5.0
7.5
10.0
12.5
min

Mw/Mn 1.35295

PEG$_{44}$-*b*-PLLA$_{90}$ **5c**

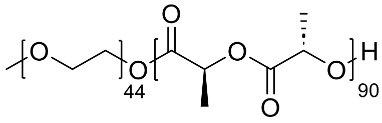

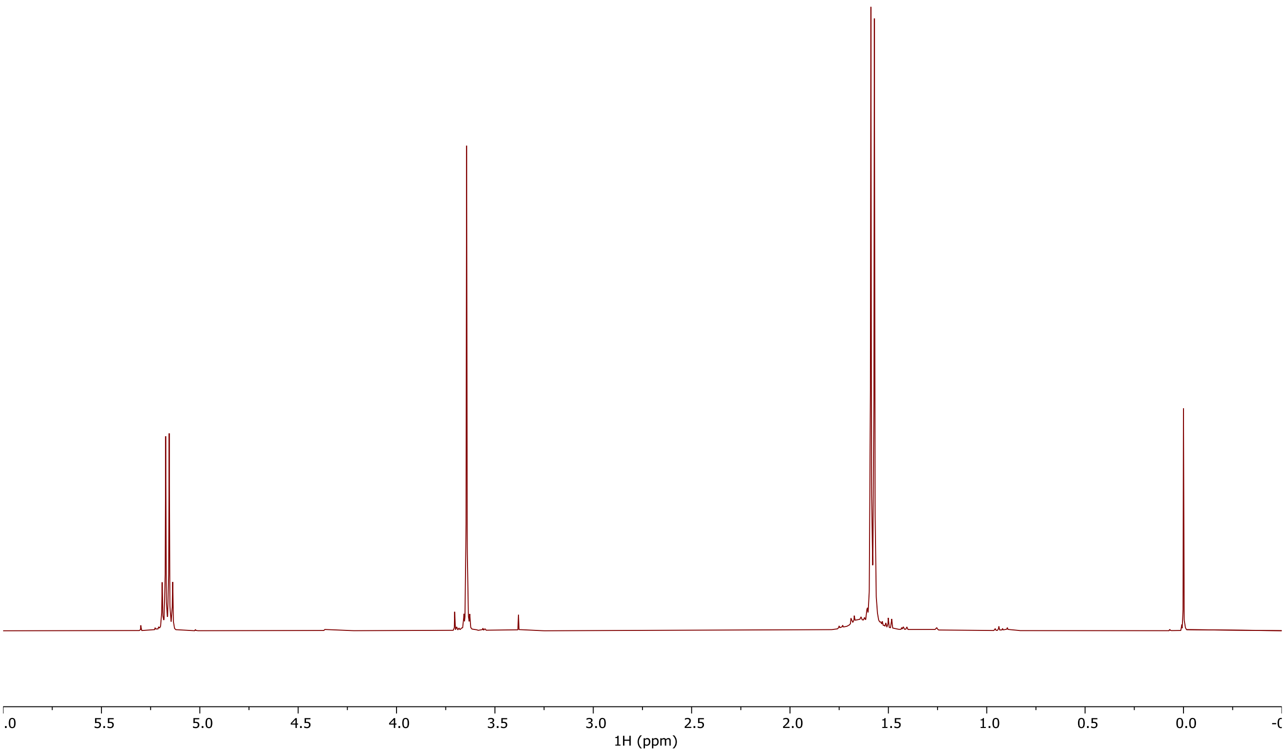

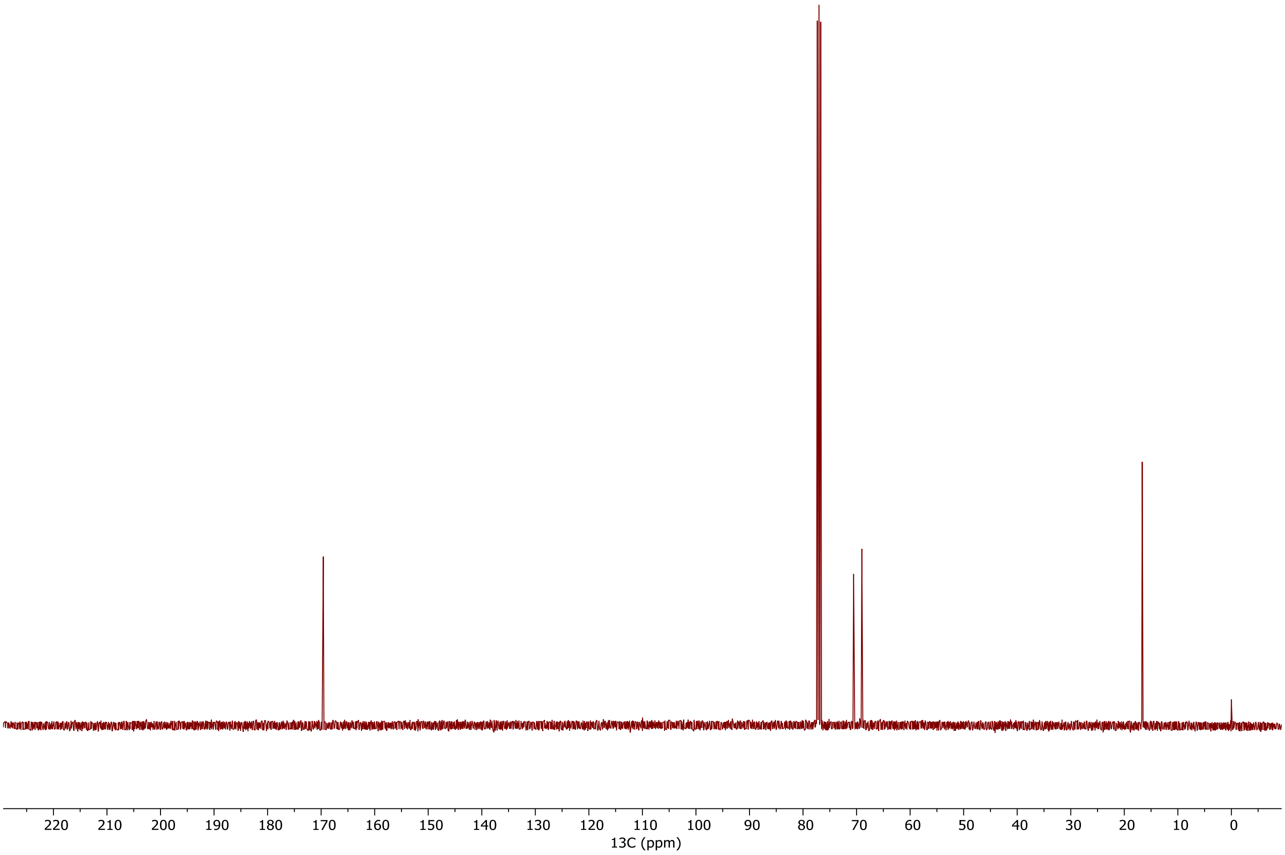
220 210 200 190 180 170 160 150 140 130 120 110 100 90 80 70 60 50 40 30 20 10 0
13C (ppm)

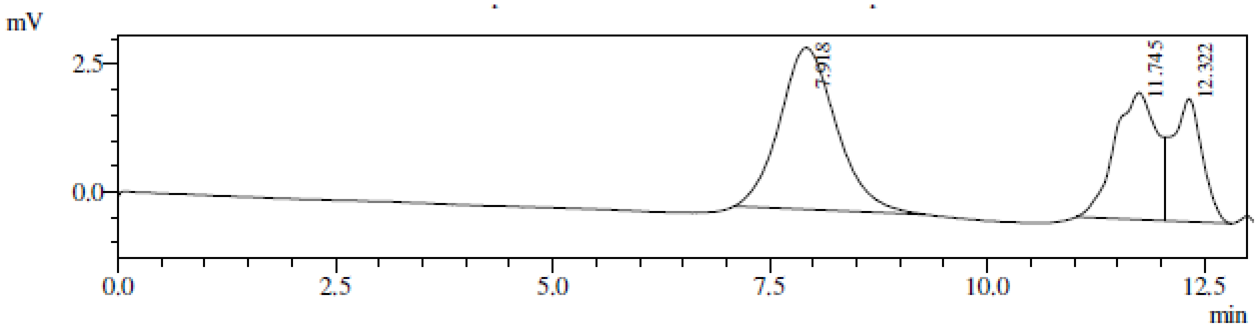
mV
2.5
0.0
7.918
11.745
12.322
0.0 2.5 5.0 7.5 10.0 12.5
min

Mw/Mn	1.11351

PEG$_{22}$-*b*-PDLA$_{45}$ **6a**

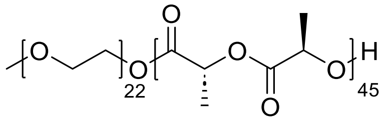

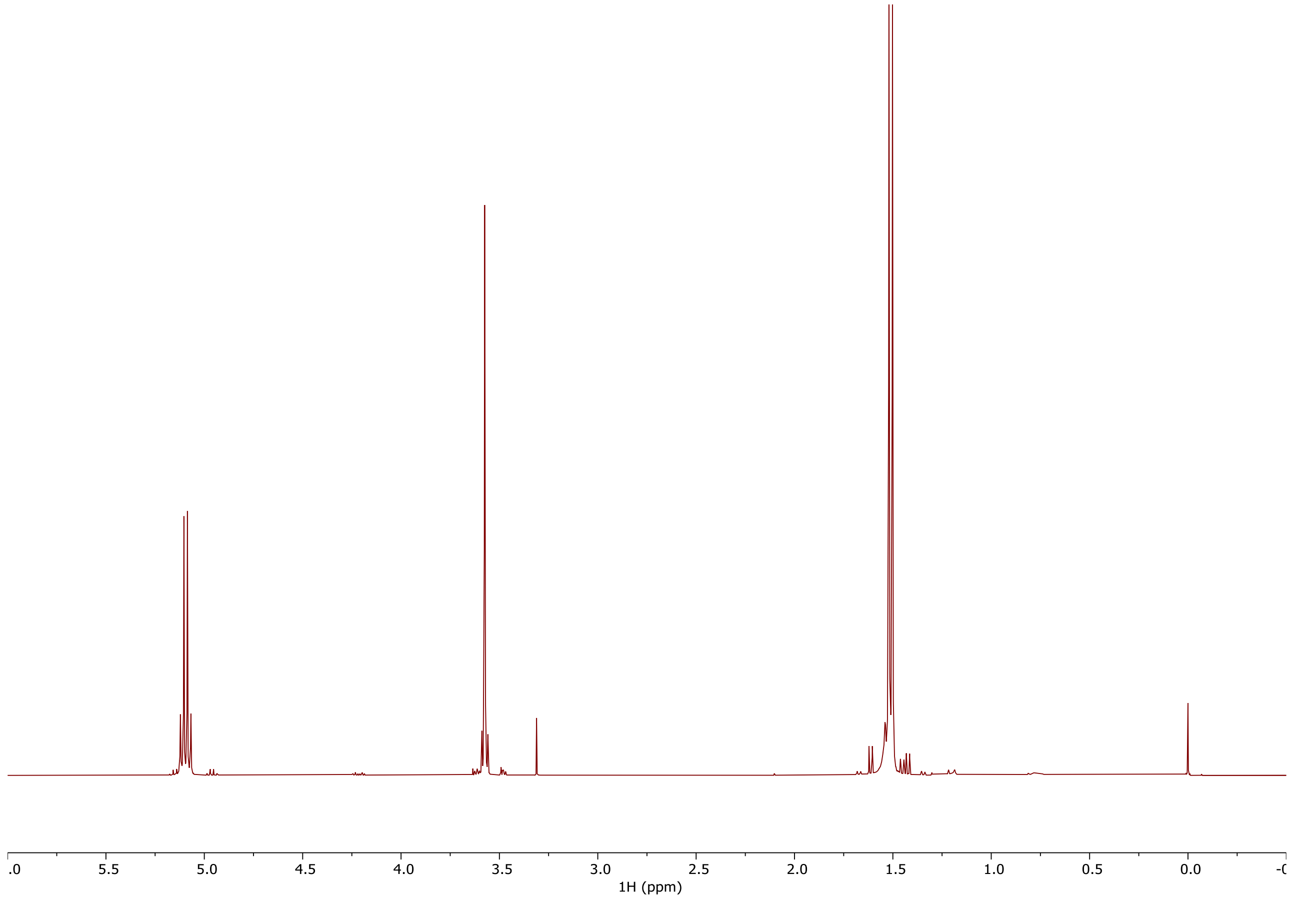

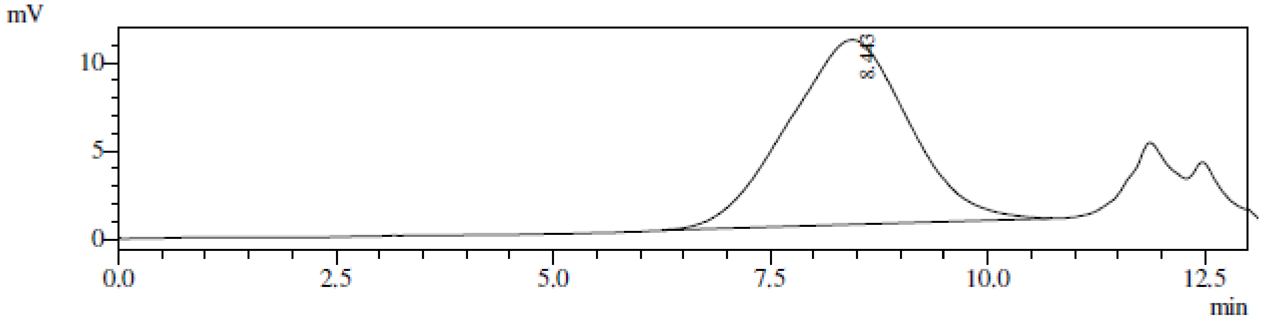

Mw/Mn	1.51635

PEG$_{22}$-*b*-PDLA$_{90}$ **6b**

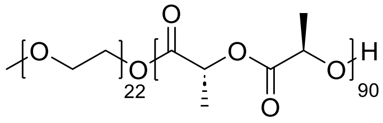

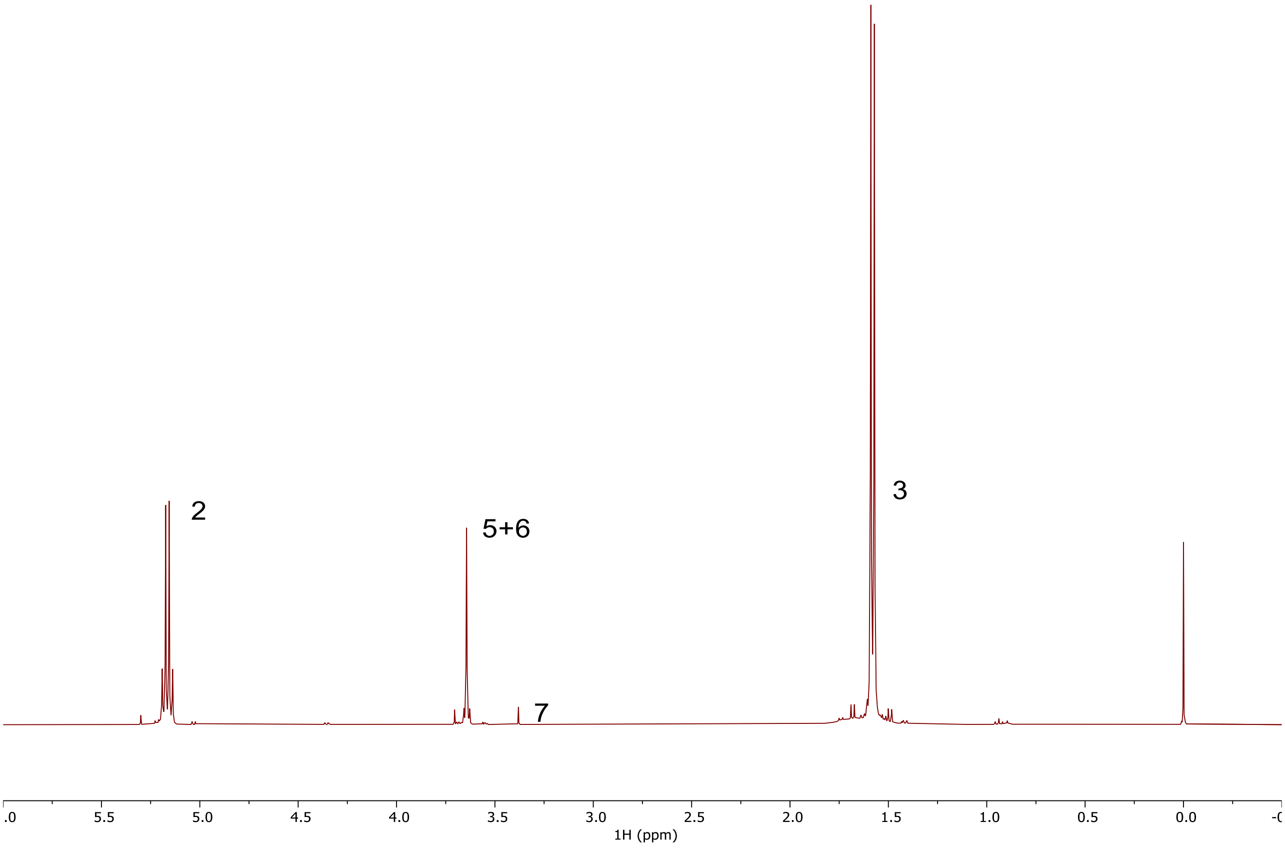

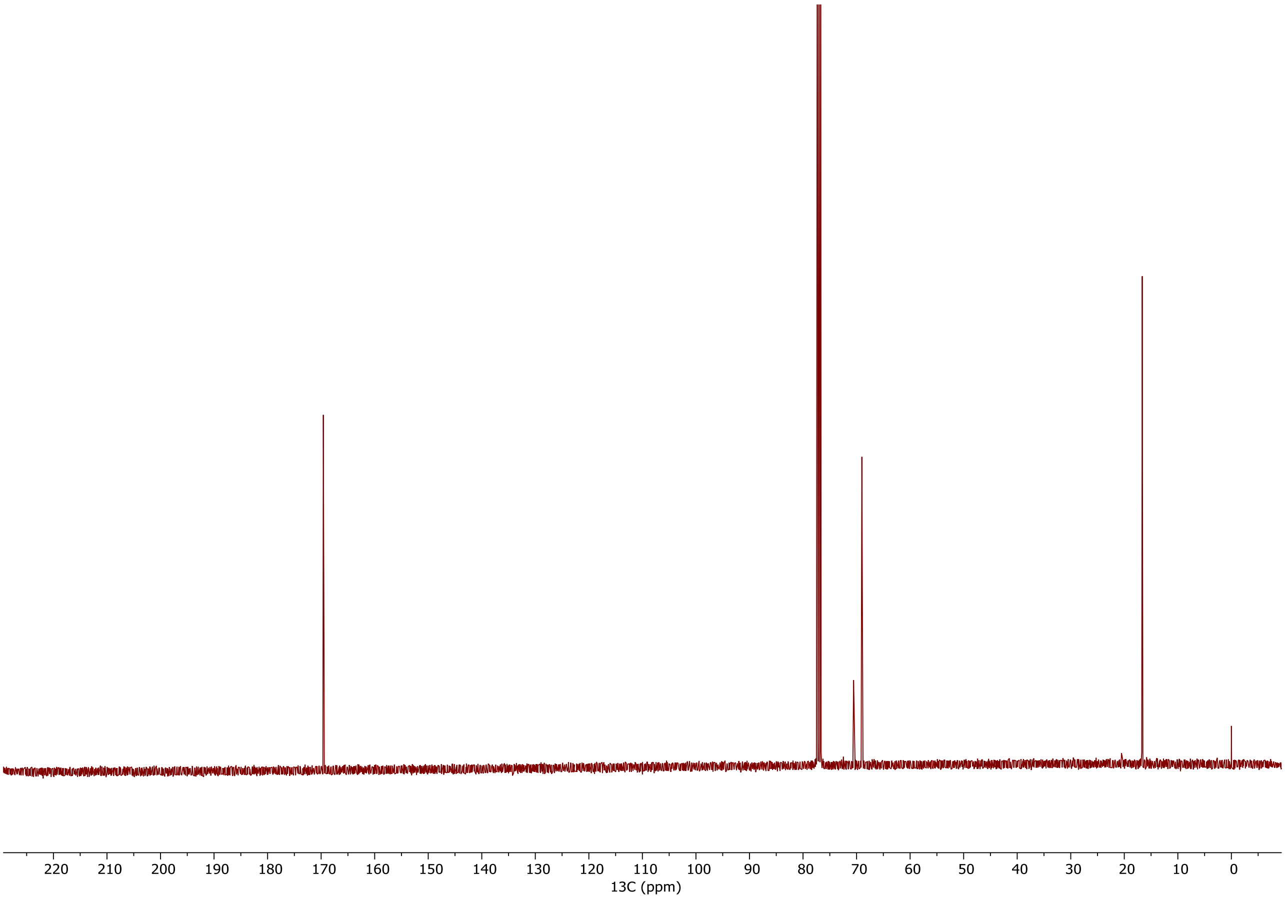
220
210
200
190
180
170
160
150
140
130
120
110
100
90
80
70
60
50
40
30
20
10
0
13C (ppm)

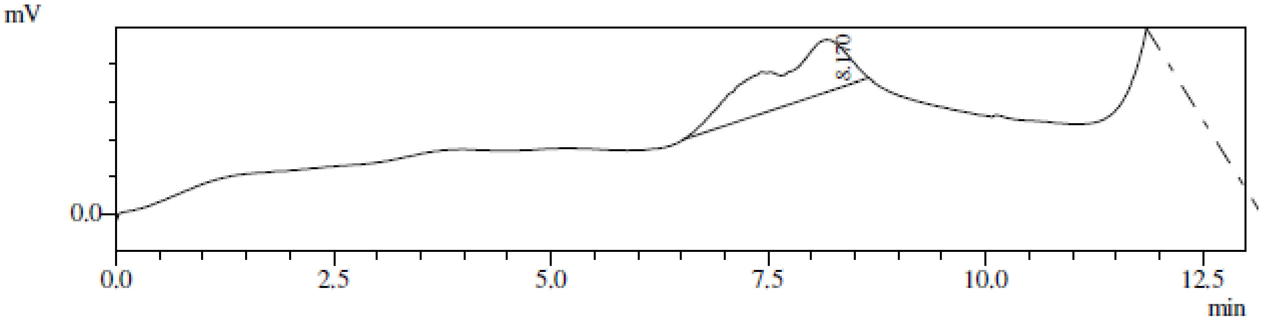
mV
0.0
8.170
0.0
2.5
5.0
7.5
10.0
12.5
min

Mw/Mn 1.25522

PEG$_{44}$-*b*-PDLA$_{90}$ **6c**

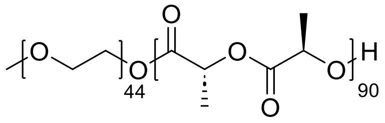

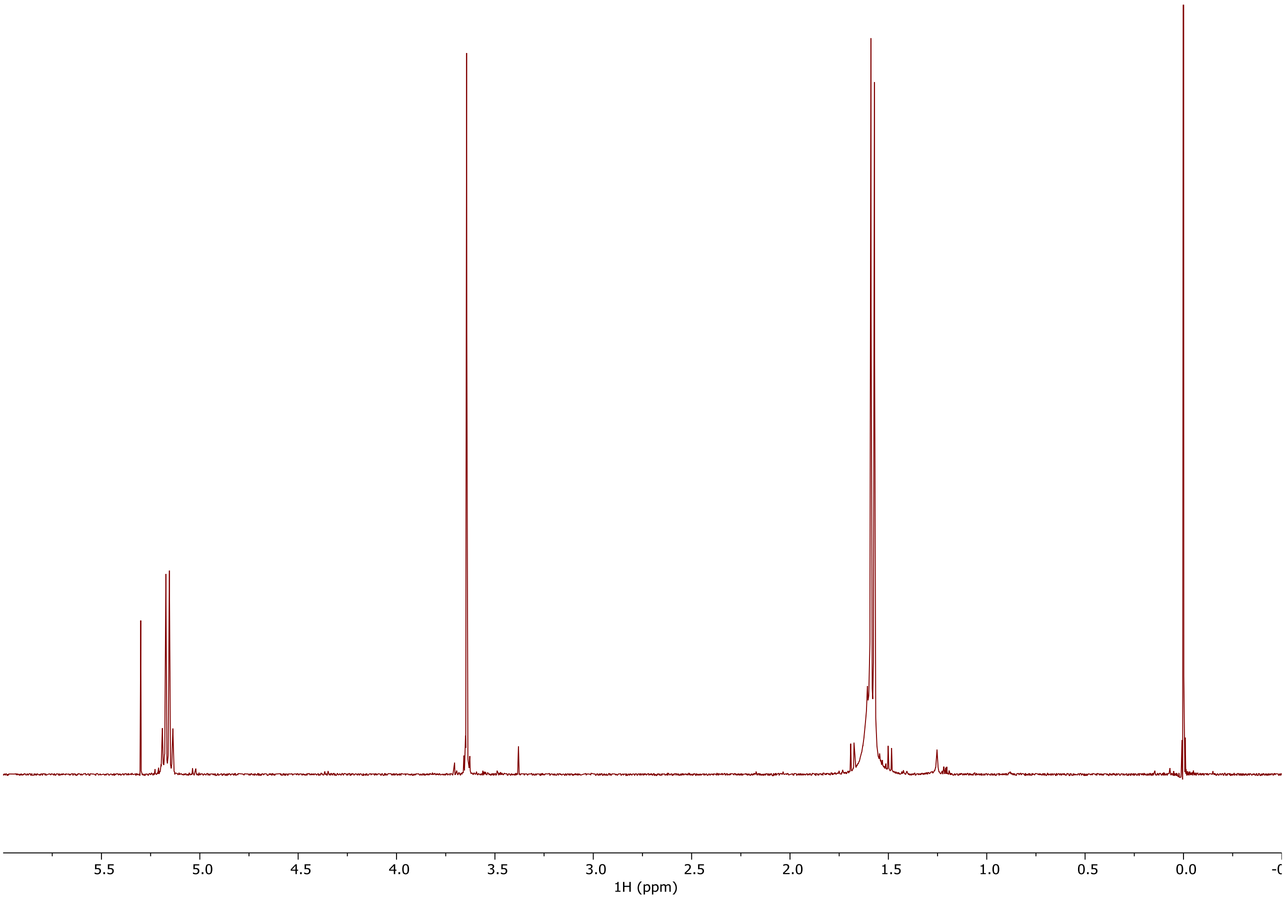

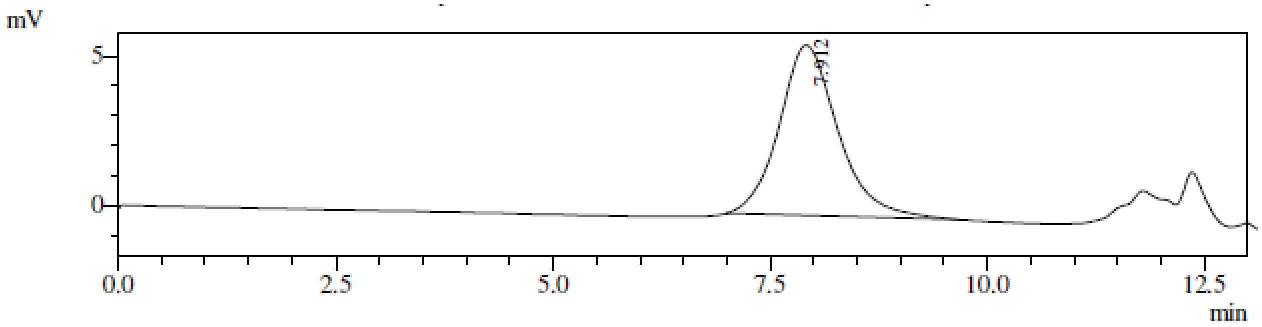

Mw/Mn 1.14349

5. References

[1] Schindelin, J.; Arganda-Carreras, I.; Frise, E.; Kaynig, V.; Longair, M.; Pietzsch, T.; Preibisch, S.; Rueden, C.; Saalfeld, S.; Schmid, B.; Tinevez, J.-Y.; White, D. J.; Hartenstein, V.; Eliceiri, K.; Tomancak, P.; Cardona, A., Fiji: an open-source platform for biological-image analysis. *Nat. Methods* **2012,** *9*, 676.
